# Extensive intraspecific gene order and gene structural variations in upland cotton cultivars

**DOI:** 10.1038/s41467-019-10820-x

**Published:** 2019-07-05

**Authors:** Zhaoen Yang, Xiaoyang Ge, Zuoren Yang, Wenqiang Qin, Gaofei Sun, Zhi Wang, Zhi Li, Ji Liu, Jie Wu, Ye Wang, Lili Lu, Peng Wang, Huijuan Mo, Xueyan Zhang, Fuguang Li

**Affiliations:** 1 Zhengzhou Research Base, State Key Laboratory of Cotton Biology, Zhengzhou University, Zhengzhou, 450001 China; 2 Institute of Cotton Research of the Chinese Academy of Agricultural Sciences, Anyang, 455000 China; 3 Anyang Institute of Technology, Anyang, 455000 China

**Keywords:** Structural variation, Polyploidy in plants, Agricultural genetics, Comparative genomics

## Abstract

Multiple cotton genomes (diploid and tetraploid) have been assembled. However, genomic variations between cultivars of allotetraploid upland cotton (*Gossypium hirsutum* L.), the most widely planted cotton species in the world, remain unexplored. Here, we use single-molecule long read and Hi-C sequencing technologies to assemble genomes of the two upland cotton cultivars TM-1 and zhongmiansuo24 (ZM24). Comparisons among TM-1 and ZM24 assemblies and the genomes of the diploid ancestors reveal a large amount of genetic variations. Among them, the top three longest structural variations are located on chromosome A08 of the tetraploid upland cotton, which account for ~30% total length of this chromosome. Haplotype analyses of the mapping population derived from these two cultivars and the germplasm panel show suppressed recombination rates in this region. This study provides additional genomic resources for the community, and the identified genetic variations, especially the reduced meiotic recombination on chromosome A08, will help future breeding.

## Abstract (web summary)

While multiple cotton genomes are available, genome wide variation comparison between allotetraploid upland cotton cultivars remain unexplored. Here, the authors assemble two upland cotton cultivars and reveal large scale structural variations on chromosome A08.

## Introduction

Upland cotton (*G. hirsutum*) is not only the main source of renewable textile fiber but also an excellent experimental system for studying polyploidization^1–3^. This allotetraploid cotton species is derived from a single polyploidization event that united the Old World A genome (resembling *G. arboreum*) and the New World D-genome (resembling *G. raimondii*) around 1∼2 million years ago (MYA)^4,5^. Draft assemblies for both A and D genomes have been reported^2,6–8^. To date, much of the work on the genomics of upland cotton has focused on the genetic standard TM-1^9^. Its draft genome assembly was released in 2015^10,11^ and the improved reference genome was released this year^12,13^. Altogether, these genomic resources have enabled a new era of studies to dissect the mechanistic basis of multiple economically and scientifically important traits.

The lower genetic diversity of cotton germplasm relative to many other crop species has made its genetic improvement particularly challenging^14–16^. One solution to this problem is to increase resolution of cotton breeding through identification of related genomic features. To approach this objective, we assemble genomes of two upland cotton cultivars: the genetic standard TM-1 and the biotechnologically important cultivar ZM24^17,18^. Unlike TM-1, ZM24 is easy for transformation^19,20^. Given a majority of the cotton acreage in many countries is now planted with transgenic cultivars (http://www.isaaa.org/resources/), sequencing ZM24 genome and exploring the underlying reasons leading to different transformation ability from TM-1 can further promote the application of biotechnology in cotton production.

In this study, we employ PacBio SMART long read^21^ and high throughput chromosome conformation capture (Hi-C) technologies^22^ to assemble genomes of TM-1 and ZM24. We anchor ~2286 and ~2295 Mb of the assemblies onto 26 pseudochromosomes of TM-1 and ZM24, respectively, which are estimated to cover more than 99% of the assemblies. Beyond a total of 907,682 SNPs, 99,329 InDels, and more than 100 Mb of presence–absence variation (PAVs; fragment length > 100 bp), we identify large inversions on A08 chromosome between ZM24 and TM-1 assemblies. Interestingly, haplotype grouping within this inverted region and more than 300,000 SNPs-based phylogenetic analysis have the same capability to divide a large collection of upland cotton germplasm into the same clusters. Genomic resources developed here can promote both basic plant biology research and applied cotton breeding.

## Results

### Genome sequencing and assembly

We produced ~205 and ~125 Gb long reads, respectively, for TM-1 (~89×) and ZM24 (~54×) using the Pacbio SMART platform. A total of 1823 and 3718 contigs were obtained for TM-1 and ZM24. After correction using the Illumina short reads, we generated a TM-1 genome of 2286 Mb with a contig N50 of 4760 Kb; the corresponding values for ZM24 were 2309 Mb and 1976 Kb (Table 1 and Supplementary Table 1).

**Table 1:** Global statistical comparison of TM-1 and ZM24 genomes

Category	TM-1 genome					ZM24 genome				
	Numbers	N50 (Kb)	Longest (Mb)	Size (Mb)	Percentage of assembly	Numbers	N50 (Kb)	Longest (Mb)	Size (Mb)	Percentage of assembly
Contigs	1283	4760	23	2286	100	3718	1976	14	2309	100
Anchored and oriented	699	4856	23	2226	97.4	1497	2151	14	2150	93.2
Gene annotated	73,624			228	10.0	73,707			243	10.5
Repeat sequence	NA^a^	NA	NA	1686	73.7	NA	NA	NA	1665	72.1

^a^ not applicable.

Hi-C libraries have been widely used to aid the assembly of contigs into chromosomes^8,23^, and we here generated >324 million and 476 million valid Hi-C interacting unique pairs for the TM-1 and ZM24 genomes, respectively (Supplementary Table 2). With the aid of Hi-C data, we anchored and oriented ~2.23 Gb of the assembly onto 26 pseudochromosomes of TM-1 (Supplementary Fig. 1), which represented 97.4% of the total assembly, including ~1.41 Gb (458 contigs) from the A_t_ subgenome and ~0.82 Gb (241 contigs) from the D_t_ subgenome. The quality of this assembly is markedly improved as compared to the previously published TM-1 genome draft assembly: the contig N50 value improved from 34.0 Kb^11^ to 4760 Kb; further, 1.9 Gb (79.2%) and 2.23 Gb (97.4%) of new sequence was oriented based on linkage maps in the TM-1 draft genome and our newly TM-1 assembly, respectively (Supplementary Figs. 3 and 4). Compared with the recently published upland cotton genome^12^, our sequences represent an improvement in contiguity (2.52-fold against the *G. hirsutum*, HAU) (Supplementary Table 3). The longer contigs from our study make the assembly more accurate. We compared the two TM-1 reference sequences (Supplementary Fig. 5) and found that except for the D08 chromosome, other chromosomes kept good collinearities with each other. A Hi-C heatmap indicated that our TM-1 was correctly assembled in the four inverted regions on D08 (Supplementary Fig. 5). We also compared our TM-1 D08 sequence with *G. raimondii*_D08^2^, TM-1_ZJU_D08^13^, Hai7124_ZJU_D08^13^, and TM-1_NAU_D08^11^, and found that our TM-1 D08 sequence showed strong collinearities with each of these assemblies (Supplementary Fig. 6).

We have 3500 more gene models in our gene prediction, and our genes models cover >95% (67,160 of 70,199) of prediction genes in the Wang et al.’s assemebly^12^. Using the same assembly strategy as we used for TM-1, 2.15 Gb (93.2%) of the assemblies from ZM24 were anchored and oriented onto 26 pseudochromosomes (Supplementary Fig. 2 and Supplementary Table 4). To evaluate our assemblies, we compared the two genomes to a previously published genetic map^24^, which revealed high consistency for each chromosome for both genomes (Supplementary Figs. 7–10). Further, the accuracy and completeness of both genome assemblies were confirmed by our finding that they each aligned perfectly with 58 completely sequenced bacterial artificial chromosome sequences (Supplementary Data 1 and 2). We also respectively mapped 78.4 and 46.2 million paired-end Illumina short reads to the TM-1 and ZM24 genomes; approximately 99% of the reads were properly mapped on each reference genome. Notably, more than 99.5% of the embryophyta genes were detectable in TM-1 and ZM24 using the BUSCO v3 dataset^25^, which were better than the recently published upland cotton reference (98.2%)^12^ (Supplementary Table 5).

Repetitive elements are widely distributed throughout most genomes and play important roles in genome divergence^26^. In general, the composition of different classes of repetitive elements in TM-1 was significantly similar to that of ZM24. Approximately 73.7 and 72.1% of the assembly sequences were annotated as repetitive sequences in the TM-1 and ZM24 assemblies, respectively, proportions lower than in *G. arboreum*^8^ but higher than in *G. raimondii*^2^. Among these, retrotransposons represented 62.8 and 61.5% of all sequences in TM-1 and ZM24 assemblies. Long terminal repeats (LTR) represented ~49.0% of all sequences of the TM-1 assembly, including *Gypsy*-type retrotransposons (41.7%) and *Copia*-type retrotransposons (7.4%), and the LTR composition of the ZM24 assembly was similar to TM-1 (Supplementary Table 6).

We next examined the transposable elements (TEs) expansion history of upland cotton genomes by estimating Kimura distance^27^ sequence divergenecy and found that TEs from *G. arboreum*, the TM-1 A_t_ subgenome, TM-1 D_t_ subgenome, ZM24 A_t_ subgenome, and ZM24 D_t_ subgenome are young and possibly very active (Supplementary Fig. 11). Furthermore, the LTRs insertion times were estimated using the EMBOSS package^28^, which respectively identified 33,908, 6933, 41,085, and 38,206 high-confidence full-length LTRs in the *G. arboreum* (A_2_), *G. raimondii* (D_5_), TM-1 and ZM24 genomes. The expansion of LTR retrotransposons in cotton occurred mainly within the past 2 MYA in the A_2_, TM-1 and ZM24 A_t_ subgenomes (Supplementary Fig. 12), and that in TM-1 and ZM24 D_t_ subgenome occurred mainly within the past 1 MYA. As compared to the D_t_ subgenome, a relatively lower percentage of young retrotransposons was observed in *G. raimondii*, a finding that is almost certainly a data artifact (Supplementary Fig. 11 and 12) because repetitive sequences collapsed when assembled from short reads, a situation similar to that reported for maize^29^. Based on TE divergence and LTR insertion times for the A genome and A_t_ subgenome (Supplementary Fig. 12), we conclude that most of the TEs expanded in the progenitor genomes and were then retained after allopolyploid formation.

### Global comparisons among four genomes

**Fig. 1:**
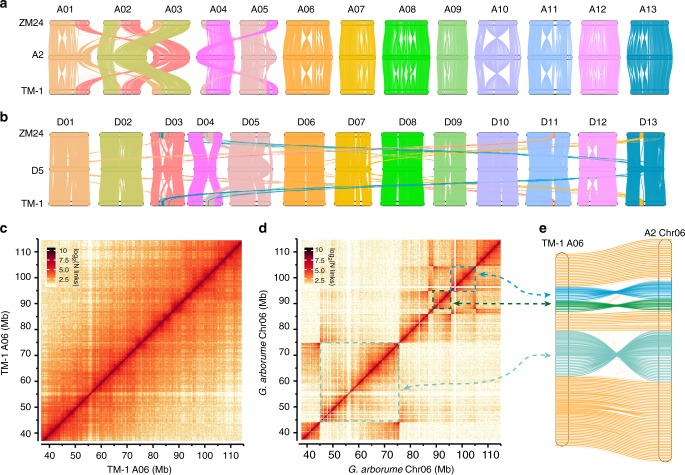
Comparison of the assembled genomes with their diploid progenitors genomes. **a** Whole genome comparison among the TM-1 A_t_ subgenome, the *G. arboreum* genome (A_2_), and the ZM24 A_t_ subgenome. **b** Whole genome comparison among the TM-1 D_t_ subgenome, *G. raimondii* genome (D_5_) and the ZM24 D_t_ subgenome. **c**, **d** TM-1 Hi-C data mapped to TM-1 A06 and *G. arboreum* Chr06. The dotted boxes indicate the inversions between A06 and Chr06. **e** Genome comparison of TM-1 A06 and *G. arboreum* Chr06. The double arrow dash line indicated the same inversions between the Hi-C map and Chromosomes. Source data of (**a**, **b**) are provided in a Source Data file

The combination of different genomes in allopolyploids is known to result in changes in genome organization, as for example in paleopolyploid maize^30^ and allopolyploid wheat^31^, Brassica^32^, and cotton^33^. Our generation of high-quality assembled genomes for upland cotton provides an opportunity to compare the A_t_ and D_t_ subgenomes with their respective ancestral genomes A_2_ (*G. arboreum*)^8^ and D_5_ (*G. raimondii*)^2^ to evaluate the possible effect of polyploidization on genomic rearrangement in cotton. The overall collinearities between the A_t_ subgenome and the A_2_ genome and between the D_t_ subgenome and the D_5_ genome are largely conserved (Fig. 1a, b and Supplementary Fig. 13). Approximately 75.3% of the TM-1 A_t_ subgenome matched in one-to-one syntenic blocks with 72.1% of the A_2_ genome. Similarly, we found approximately 78.1% of the TM-1 D_t_ subgenome matched in one-to-one syntenic blocks with ~85.6% of the D_5_ genome (Supplementary Table 7).

The nonsyntenic sequences included repetitive elements, species-specific low-copy sequences, and regions with structure variations. There were 13,819 rearrangements (translocations and inversions) between TM-1 A_t_ and A_2_; 7492 rearrangements (translocations and inversions) between TM-1 D_t_ and D_5_; and 2254 (translocations and inversions) between TM-1 and ZM24. These results clearly show that the number of interspecific rearrangements is much larger than the intraspecific rearrangements.

However, given that the total length of rearrangements between TM-1 A_t_/D_t_ and A_2_/D_5_ occupied ~620 Mb, which was ~12-fold of that between TM-1 and ZM24 (occupying 51.2 Mb), we managed this difference in the magnitude of the length of the rearranged sequences by manually combining the small aligned blocks into larger and more continuous rearrangement regions for the interspecific comparisons between TM-1 A_t_ and A_2_ (*G. arboreum*) as well as between TM-1 D_t_ with D_5_ (*G. raimondii*). A total of 39 (~324 Mb) inversions and 35 (~219 Mb) translocations were identified between the TM-1 A_t_ subgenome and the A_2_ genome compared with 15 (~48 Mb) inversions and 29 (~34 Mb) translocations between the TM-1 D_t_ subgenome and the D_5_ genome (Supplementary Data 3 and 4). Furthermore, there were at least 60 (~38.5 Mb) inversions and 1314 (~4.8 Mb) translocations identified between the intraspecific TM-1 A_t_ and ZM24 A_t_ subgenomes. However, the total lengths of the inversions and translocations between the TM-1 D_t_ and ZM24 D_t_ subgenomes were only ~4.7 and ~3.2 Mb, respectively (Supplementary Tables 8 and 9). Thus, the A-derived subgenomes have apparently been more active than D-derived subgenomes during evolution.

The rearrangements occurred across the 26 chromosomes, and most of these large-scale variations are shared by both A_t_ subgenomes and both D_t_ subgenomes in TM-1 and ZM24 (Fig. 1a), indicating that these structural rearrangements occurred after polyploidization but before the divergence of these two upland cotton accessions. Three large reciprocal translocations were identified via comparison of the A_t_ subgenome and the A_2_ genome: one has been reported in the A_t_ subgenome and involves A01 and A03^34^; the other two, between A02 and A03 and between A04 and A05, were confirmed in the A_t_ subgenomes by cytogenetic data^35^ and Hi-C (Fig. 1a and Supplementary Figs. 14 and 15). In addition, the inversions between upland cotton A06 and *G. arboreum* Chr06 were also confirmed by Hi-C (Fig. 1c–e, Supplementary Figs. 13–14). The only large-scale translocation from the *G. raimondii* (D_5_) genome was observed between D04 and D05. Note that both D04 and D05 from TM-1 and ZM24 retained conserved collinearity with *G. arboreum* (A_2_), indicating that this translocation is unique to *G. raimondii* (Fig. 1b, Supplementary Fig. 13).

### Genomic variations between TM-1 and ZM24

**Fig. 2:**
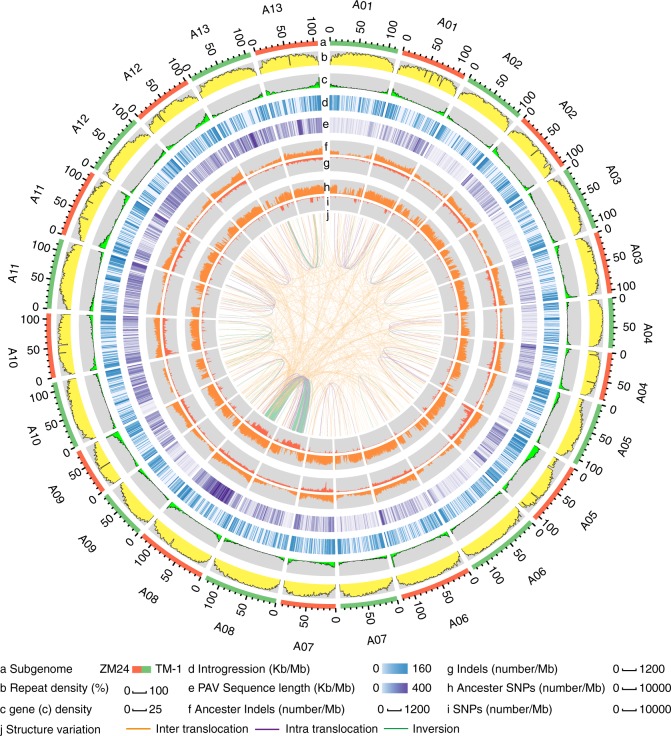
Genomic landscape between TM-1 and ZM24 genomes. **a** Subgenomes of TM-1 (green) and ZM24 (orange). **b**, **c** Transposable elements and gene density in 1 Mb sliding windows. **d** Introgression from chromosomes of the D_t_ subgenome to its counterpart chromosomes in the A_t_ subgenome in 1 Mb sliding windows. **e** Distribution of PAV sequences in 1 Mb sliding windows. **f** InDels between the TM-1 and ZM24 A_t_ subgenomes and *G. arboreum* (A_2_) in 1 Mb sliding windows. **g** InDels between TM-1 and ZM24 A_t_ subgenomes in 1 Mb sliding windows. **h** SNPs between A_t_ subgenomes and A_2_ in 1 Mb sliding windows. **i** SNPs between TM-1 and ZM24 A_t_ subgenomes in 1 Mb sliding windows. **j** Large-scale variations between the TM-1 and ZM24 subgenomes. Source data are provided in a Source Data file

When we aligned the ZM24 A_t_ subgenome with that of TM-1, 99.3% of the ZM24 genome sequence matched well in one-to-one syntenic blocks with 95.2% of the TM-1 genome sequence (Supplementary Tables 8 and 9). Similarly, 98.1% of the ZM24 D_t_ subgenome matched well in one-to-one syntenic blocks with 94.3% of the TM-1 D_t_ subgenome, suggesting that most regions of the genome are stable in upland cotton. There were 127 inversions, 234 intra-translocations, and 1893 inter-translocations between the TM-1 and ZM24 genomes that together occupied ~51.2 Mb (Supplementary Table 9). The top three longest large-scale structural variations (Fig. 2 and Supplementary Fig. 13) were from A08, and the total length of these regions accounted for ~30% of the total length of TM-1 A08 and fully ~71.8% of the total nonsyntenic sequence. TEs are impactful for generating genomic novelty because they drive chromosomal rearrangements. To examine whether TEs have been involved in structural variations, we analyzed the TEs composition around the breakpoints of the observed rearrangements and found that the TEs content especially the *Gypsy*-type LTRs in the rearrangement regions were higher than the average for the whole genome (for both the A_t_ and D_t_ subgenomes; Supplementary Tables 10 and 11).

We next detected PAVs and identified a total of 7953 of TM-1-specific genomic PAVs and 13,160 of ZM24-specific genomic PAVs. Most of the PAVs were shorter than 10 Kb. A total of 1101 and 2327 PAVs were identified that were longer than 10 Kb in TM-1 and ZM24, respectively. The PAV distributions across the chromosomes were uneven, for example ZM24 A08 (1847 PAVs, ~7.9 Mb) had significantly more PAVs than the other chromosomes (Fig. 2, Supplementary Fig. 16, Supplementary Table 12). Further, compared with TM-1 A08, ZM24 A08 has more PAVs, especially in the inversion regions, indicating that structural variation is associated with PAVs. The number of PAVs between TM-1 D_t_ subgenome (4875) and ZM24 D_t_ subgenome (4277) was similar (Supplementary Table 12), a result indicating that more extensive genetic variation has occurred in the A_t_ subgenome relative to the D_t_ subgenome. The longest ZM24-specific segment from a pseudochromosome was a ~46.7 Kb segment on D08 and a ~43.95 Kb segment on A08. The longest TM-1-specific segment on A13 was a ~44.6 Kb segment and was ~46.3 Kb on D08.

A total of 744 and 635 genes were identified as ZM24- or TM-1-specific genes (Supplementary Data 5 and 6). We aligned the genes located on pseudochromosomes with the *G. arboreum* and *G. raimondii* genomes and approximately 58 and 69% of the PAV genes from the TM-1 A_t_ subgenome and ZM24 A_t_ subgenome have likely orthologs in *G. arboreum*. Similarly, at least 73 and 61% of TM-1 and ZM24 D_t_ subgenome genes have likely orthologs in *G. raimondii*, indicating that a majority of these PAV genes have already existed in the ancestral genomes. The PAV genes without obvious orthologs in the diploid cottons have likely arisen since allotetraplod formation.

We identified a total of ~8.3 Mb of introgressions from the A_t_ subgenome to the D_t_ subgenome and ~7.8 Mb of introgressions from the D_t_ subgenome to the A_t_ subgenome (Fig. 2 and Supplementary Fig. 17). Similarly, a total of ~8.8 and ~7.0 Mb introgression segments were identified between the ZM24 A_t_ and D_t_ subgenomes. It is thus clear that there are more introgressions from the A_t_ subgenome to the D_t_ subgenome than from the D_t_ subgenome to the A_t_ subgenome.

We identified a total of 907,682 SNPs and 99,329 InDels in aligned syntenic blocks between TM-1 and ZM24, with an average of 0.44 SNPs and 0.048 InDel per Kb (Supplementary Table 9). Compared with the SNP densities across the whole genome (0.50 per Kb), the SNP density for A08 (2.03 per Kb) was significantly higher (Fig. 2). Almost all of the InDels were one base-pair in length (Fig. 2 and Supplementary Fig. 17). Like the SNPs, the InDel densities on A08 were also significantly higher than for other chromosomes (Fig. 2, Supplementary Fig. 18). Moreover, an insertion cluster hotspot was found on ZM24 A05 (0~7 Mb); it contained ~7600 InDels (~1.08 per Kb), a higher density than the average for the whole genome (0.049 per Kb) (Supplementary Fig. 19). Similarly, an InDel hotspot was found on A05 of TM-1 (Supplementary Fig. 18).

### Gene-order and structural variation between TM-1 and ZM24

To analyze gene order, a total of 71,794 orthologous pairs were identified between TM-1 and ZM24, among which 58,913 orthologous gene pairs were significantly conserved in the four subgenomes (Supplementary Table 13). A total of 5570 and 5400 orthologous gene pairs were only present in the A_t_ subgenomes and the D_t_ subgenomes, respectively. A total of 34,634 (~96.1%) orthologous pairs within the A_t_ subgenomes and 34,518 (~96.3%) orthologous gene pairs within the D_t_ subgenomes were anchored onto the 26 pseudochromosomes, and were used for gene-order and structure comparisons which revealed 34,243 and 34,156 that genes have, respectively, corresponding orthologs in their syntenic blocks of the A_t_ and D_t_ subgenomes. Furthermore, 391 and 362 orthologous gene pairs from the A_t_ or D_t_ subgenomes were identified in the nonsyntenic regions; these accounted for ~1.1 and ~1.0% of the total analyzed orthologous gene pairs for the A_t_ and D_t_ subgenomes, respectively. These genes were enriched in several metabolism pathways including Vitamin B6 metabolism and glycosaminoglycan degradation (Supplementary Figs. 20 and 21, Supplementary Data 7).

Among the syntenic genes, 23,963 orthologous gene pairs from A_t_ subgenomes displayed no amino acid changes between TM-1 and ZM24, and approximately 68 and 56% showed no variation in coding sequence (CDS) or gene bodies (CDS and intron region), respectively. Similarly, we found 24,144 (~71%) orthologous gene pairs without any such changes in amino acids in the D_t_ subgenome, among which ~69 and 57% showed no variation in CDS or gene bodies, respectively. We also identified 16,014 highly conserved orthologous gene pairs between TM-1 and ZM24 that had no variation in their entire genic regions. Furthermore, we identified 3465 and 3296 orthologous gene pairs with only missense mutations in their CDS /or non-frameshift InDels between the two A_t_ subgenomes or between the two D_t_ subgenomes (Table 2). These genes, together with genes lacking any amino acids changes, were classified as structurally conserved genes, as reported in maize^29^.

**Table 2:** Variations within genes between TM-1 and ZM24 genomes

Variation type	Syntenic orthologous gene pairs				Nonsyntenic orthologous gene pairs			
	At	Percent	Dt	Percent	At	Percent	Dt	Percent
Structurally conserved genes	27,428	80.10%	27,440	80.34%	222	56.78%	160	44.19%
Without amino acid substitutions	23,963	69.98%	24,144	70.69%	104	26.60%	60	16.57%
No DNA variation in CDS region	23,290	68.01%	23,463	68.69%	77	19.69%	45	12.40%
No DNA variation in CDS and intron region	19,130	55.87%	19,415	56.84%	41	10.49%	27	7.45%
No DNA variation in genic region ^a^	7712	22.52%	8302	24.31%	2	0.51%	10	2.76%
Same sense mutation	673	1.97%	681	1.99%	27	6.91%	15	4.14%
With amino acid changes	3465	10.12%	3296	9.65%	118	30.18%	100	27.55%
With missense mutation in CDS	2340	6.83%	2222	6.51%	97	24.81%	78	21.62%
With 3n InDel in CDS	1125	3.29%	1074	3.14%	21	5.37%	22	6.07%
Genes with large-effect mutations	2185	6.38%	2214	6.48%	48	12.28%	62	17.12%
With 3n ± 1 InDel in CDS	629	1.84%	550	1.61%	17	4.35%	19	5.24%
Start-codon mutation	921	2.69%	1127	3.30%	11	2.81%	21	5.80%
Stop-codon mutation	390	1.14%	327	0.96%	15	3.84%	15	4.14%
Splice-acceptor mutation	23	0.07%	20	0.06%	1	0.26%	0	0.00%
Splice-donor mutation	222	0.65%	190	0.56%	4	1.02%	7	1.93%
Genes with large structural variations	4630	13.52%	4502	13.18%	121	30.95%	140	38.67%
At least one CDS missing	4370	12.76%	4164	12.19%	90	23.02%	104	28.73%
Total	34,243^b^	100.00%	34,156^b^	100.00%	391^b^	100.00%	362^b^	100.00%

^a^Genic regions include 2 kb upstream and downstream of the gene body

^b^Only genes and their orthologs in the counterpart genome anchored in 26 chromosomes were included for the analysis

These structurally conserved genes accounted for ~80% of syntenic orthologous gene pairs. However, we found that ~6.4% of the syntenic orthologous gene pairs with large-effect mutations, including start-codon mutations, stop-codon mutations, splice-donor mutations, splice acceptor mutations, and frameshift mutations. Additionally, >13% of the syntenic orthologous gene pairs had large structural variations, with 93.4% of these having lost at least one exon (Table 2). This intra-cultivar structural variation is higher than that between two maize cultivars of the diploid species (Mo17 and B73), lending support to previous proposals about accelerated gene evolution in the wake of polyploidization events^36^.We also analyzed the structurally conserved genes in nonsyntenic regions between ZM24 and TM-1. We classified 382 orthologous gene pairs as structurally conserved genes (Table 2). However, ~12% of the A_t_ subgenome orthologous gene pairs and ~17% of the D_t_ subgenome orthologous gene pairs had large-effect mutations, and ~31% of the A_t_ subgenome orthologous gene pairs and ~39% of the D_t_ subgenome orthologous gene pairs had large structural variations. In summary, we identified a total of 13,902 orthologous gene pairs with either large-effect mutations or large structural variations. Approximately 10% of the annotated orthologous gene pairs had amino acid variation between TM-1 and ZM24; any biological significance of these variations will require further study.

We also checked the extent of gene amplification in the TM-1 and ZM24 genomes. In total, 6532 and 6938 genes were identified as singleton genes in TM-1 and ZM24, respectively (Supplementary Table 14). Most of the genes were derived from dispersed duplications (9640 from TM-1 and 10,812 from ZM24) or whole genome duplications (WGD) and segmental duplications (SD; 49,238 from TM-1 and 47,291 from ZM24). The other groups were proximal duplications or tandem duplications. It was notable that there was a much higher proportion of transcription factors than singleton genes among the whole genome duplication and segmental duplication sets, indicating for cotton that transcription factors have a tendency to be retained after WGD or SD, similar to previous reported findings for maize^29^.

Although establishment of a high-regeneration-frequency system of somatic embryogenesis (SE) has facilitated the generation of transgenic cotton, substantial challenges for cotton transformation remain, including genotype limitations and developmental asynochronization^19^. To probe for potential mechanisms underlying the relative ease-of-transformation of ZM24 over TM-1, we conducted mRNA-seq profiling experiments with plant materials sampled during SE: the differentially expressed genes (DEGs) were analyzed between callus (Day 20 of culturing) and embryogenic callus (Day 50 of culturing) for TM-1 and ZM24. It should be emphasized that the Day 50 TM-1 calli appeared green in color and had a hard texture; that is, these samples exhibited the expected inability to develop into embryogenic calli. Generally, this transcriptome analysis revealed that although there were a few hundred DEGs detected for the comparison between TM-1 and ZM24 calli (Day 20 of culturing), an order of magnitude more DEGs were detected in the comparison between the ZM24 embryogenic calli and the Day 50 TM-1 calli, results consistent with the enormous morphological differences between the sample types.

Given previously reported highly impactful genotype-specific transformation-efficiency-related roles for hormone metabolism genes in cotton^37^ and soybean^38^, we focused on DEGs with such annotations. Given its known auxin-mediated regulatory contribution to embryogenic callus development^37^, it was particular notable that transcripts from six loci for *LEC1*–*like* genes (Gh_D05G177600, Gh_A05G160900, Gh_A08G030500, Gh_D08G030600, Gh_D13G152700, Gh_A13G151400) were expressed at significantly higher levels in ZM24 than TM-1 for the Day 50 calli. Moreover we found, for ZM24 but not TM-1, that the well-characterized downstream target of *LEC1*, *GhPIN1* (Gh_A11G002000), was among the DEGs between the Day 20 and Day 50 calli. Two auxin biosynthesis genes *GhYUC10* (Gh_D08G133100, Gh_A08G079800) were also ZM24-specific DEGs in between Day 20 vs. Day 50 comparison, further implicating auxin accumulation and transport apparently via the *LEC1* mediated regulation of auxin biosynthesis (*GhYUC10*) and auxin polar transport (*GhPIN1*) in promoting of cotton transformation efficiency (Supplementary Fig. 22). This hypothesis could be tested by overexpressing *GhLEC1* genes in calli of low-transformation-efficiency cultivars and monitoring changes in transformation efficiency, with or without attendant chemical genetics experiments that directly alter auxin levels.

Unlike auxin, for which increased anabolic metabolism promotes embryogenic callus development, it has been shown that gibberellins (GA) catabolism promotes somatic embryogenesis^38^. It is thus consistent that we detected ZM24-specific increases in the levels of transcript from four loci encoding homologs of the known GA-catabolism-regulating transcription factor *GhAGL15* (Gh_A12G116000, Gh_D12G115800, Gh_D08G112500, Gh_A08G163400) in the Day 50 calli. Similar ZM24-specific increases were observed for transcripts of enzymes that catabolically inactivate GAs, including *GhGA2ox1* (Gh_A01G038100) and *GhGA2ox4* (Gh_A07G139000). It thus seems that the active GA content is lower in ZM24 calli than in the difficult to transform TM-1 cultivar at Day 50 (Supplementary Fig. 22). This hypothesis could be evaluated by overexpressing *GhAGL15* or *GhGA2ox* genes in the low-transformation-efficiency genotype and/or by manipulating GA levels directly via application or use of GA-biosynthesis-inhibitors. Highlighting the utility of comparative genomics amongst multiple cultivars from a given species, it was highly notable that we detected genetic polymorphisms between the TM-1 and ZM24 cultivars for many of these transformation-barrier related genes from hormone metabolism: for example SNPs in the coding region of *GhAGL15* (Gh_A12G116000 and Gh_A08G163400) and SNPs or InDels in the promoters of *GhAGL15* (Gh_A12G116000 and Gh_D08G112500) and *GhLEC1* (Gh_A13G151400).

### Large structural variations lead to isolated haplotypes

**Fig. 3:**
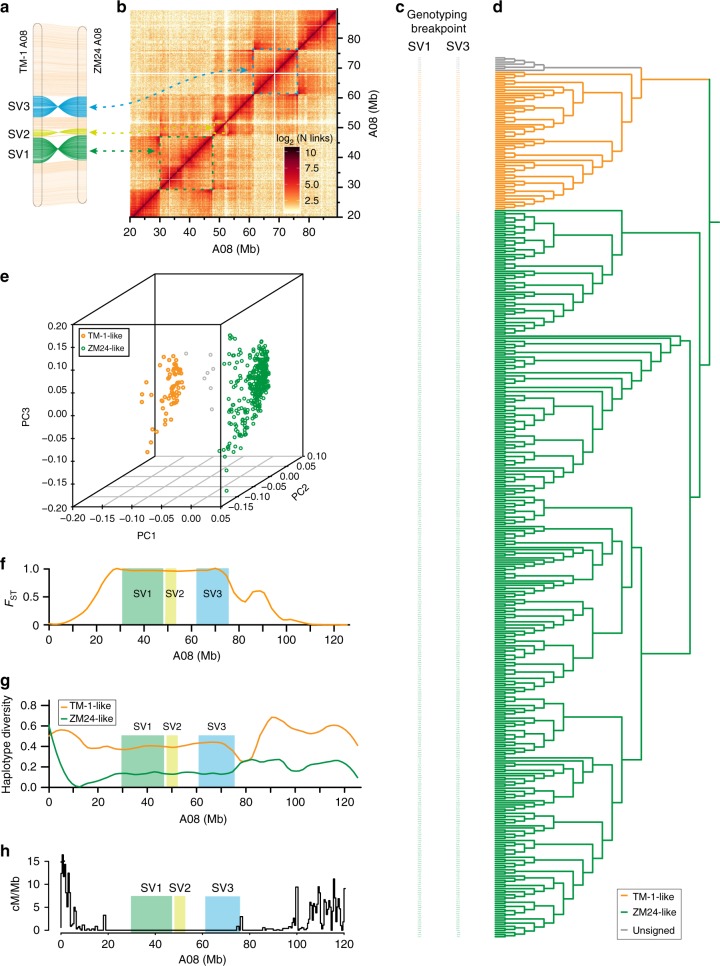
Genetic effect of inversions present on A08. **a** Large-scale inversions (SV1, SV2, and SV3) on A08 between TM-1 and ZM24. **b** ZM24 Hi-C data mapped to TM-1 A08. **c** Genotyping of breakpoints in SV1 and SV3. The 421 accessions colored by the A08 inversion allele (orange, TM-1-like allele; green, ZM24-like allele; grey, unsigned) as assessed by the breakpoints for SV1 and SV3. The accessions were ordered based on the haplotype clustering shown in (**d**). **d** Haplotype clustering based on 315,868 SNPs (MAF ≥ 0.05) genome-wide, revealing two distinct clusters (TM-like and ZM24-like groups), which identically matched the distributions of the two A08 inversion alleles. **e** Principal component analysis of 421 accessions. **f** Genetic differentiation (F_*ST*_) between the TM-1-like group and ZM24-like group. **g** Haplotype diversity for the TM-1-like and ZM24-like group. **h** Local impact of large inversions on meiotic recombination rate using a RIL population derived from a cross of TM-1 and ZM24. Black line, cM/Mb values across the chromosome; Colored boxes, location of inversions. Source data of (**e**–**h**) are provided in a Source Data file

Three large-scale inversions (larger than 4 Mb) were found on A08 in the tetraploid assemblies; these were confirmed by mapping the Hi-C data of one accession to the genome of the other and vice versa (Fig. 3a, Supplementary Figs. 23 and 24). Enlargements from the heatmaps revealed discontinuous signals for these inversions (in the region marked by the color boxes in Fig. 3b); we accurately identified the left and right breakpoints of SV1 and SV3 by comparison of genomic sequences, and these were further confirmed with the PacBio long read data, with PCR, and with Sanger-sequencing (Supplementary Figs. 25 and 26).

It is known that selection pressure on one of the alleles in a structurally variant region can significantly affect its allele frequency and haplotype diversities across entire inverted regions^39^. We therefore conducted a haplotype analysis of worldwide collection of 419 germplasm diversity accessions^40^ using whole genome resequencing data. Specifically, for each of the inversion breakpoints, we determined whether or not the mate-pair reads spanned the TM-1-like breakpoints. Notably, SV1 is genetically linked with SV3, most of the short reads simultaneously supported the co-occurrence SV1 and SV3 in a given upland cotton accession; this analysis identified 66 TM-1-like accessions (without SV1 or SV3) and 348 ZM24-like accessions that carried both of these large insertions. (Fig. 3c and Supplementary Figs. 27–30), and none of the accessions showed contradicting genotypes for these two inversions. We identified a total of 226 and 248 genes from ZM24 and TM-1 in the regions spanning SV1 and SV3, thusly indicating a serious gene loss in structural variation regions (Supplementary Data 8 and 9).

Excitingly, and strongly supporting the utility of these inversions for differentiating meaningful haplotypes among these germplasm diversity panel accessions, when we conducted a phylogenetic analysis (neighbor joining) based on a total of 315,868 SNPs from across the genome. The subclades identified in this analysis matched very well with the groupings based on the large-scale inversions on A08 (Fig. 3d). Moreover, principal component analysis (PCA) also strongly supported the classification from both the A08 haplotype and SNP-based phylogenetic analyses (Fig. 3e). Of considerable note, we found that TM-1-like genotype and ZM24-like genotype showed significant genetic differentiation (F_*ST*_), and this divergency tendency was also evident in regions neighboring the breakpoints (Fig. 3f, Supplementary Fig. 31). Haplotype diversities of ZM24-like genotype were greatly reduced in the inverted regions, and, again the effect was evident in regions neighboring the breakpoints (Fig. 3g and Supplementary Fig. 32).

Next, consider the fact that meiotic recombination is known to be dramatically suppressed in inverted genome regions; moreover, consider that suppression is known to have a minimizing effect on genetic exchange between two inverted alleles^39^. Despite the known strong genetic impacts of such regions, we are unaware of any reports about large-scale analysis of such inversions in upland cotton, likely owing to the lack of genome assemblies of sufficient quality to facilitate the identification of breakpoints. To explore these ideas experimentally, we generated a population of 181 RILs by crossing TM-1 and ZM24, which were used to calculate recombination frequencies. When we overlapped the SV1 and SV3 regions with meiotic recombination frequency data between TM-1 and ZM24, we observed strong associations between suppressed recombination rates and the loci contained within the inversion regions; this suppressed recombination tendency was also evident in regions neighboring the breakpoints (Fig. 3h). Polymorphisms that are located outside inverted regions but are near chromosomal breakpoints tend to have decreased genetic exchange relative to the whole genome, a situation similar to polymorphisms found within inversions^41^.

To further study the relationship between inversion and genetic diversity, we counted the SNPs on A08 within both the ZM24-like and TM-1-like genotypes and found that the SNPs densities significantly decreased within the inverted regions. Moreover, we found that a substantial portion of uniquely occurring (or private) SNPs were observed within these regions for each of the groups. The distribution of private vs. common SNPs in these regions between the groups strongly indicates that inversions represent areas of restricted meiotic recombination, which has ultimately led to an overall decrease in nucleotide diversity (π) in the ZM24-like and TM-1-like groups (Supplementary Tables 15 and 16).

We also conducted an analysis of nucleotide diversity (*π*) with a sliding window (1 Mb, 10 Kb step size), which also revealed that TM-1-like genotype and ZM24-like genotype showed significantly reduced genetic diversity within the inversion regions. This tendency for reduced nucleotide diversity was also evident in regions neighboring the breakpoints (Fig. 3h, Supplementary Fig. 33). Viewed together, our recombination frequency and haplotype results collectively strongly suggest that the suppression of the recombination (and thus genetic exchange) resulting from the inversion regions on A08 have, over time, separaed the upland cotton population into two distinct groups. Consequently, there is a need to re-evaluate our understanding about how upland cotton divergence has occurred and how this process has specifically influenced the extant nucleotide and trait diversity present in the upland cotton population.

## Discussion

We here report genome assemblies for two centrally important varieties of upland cotton (the genetic standard TM-1 and the biotechnologically important ZM24). These assemblies are based on long read sequencing technology and are thus more complete and enabling for a new era of cotton basic research and improvement efforts. Scientifically, the core insight revealed by these assemblies is the presence of large-scale structural variations on the 8th chromosome of the A_t_ subgenome of this allotetraploid species which can, as a single pair of alleles, accurately differentiate two major haplotypes among worldwide upland cotton accessions.

Polyploidy is a widespread phenomenon in angiosperms. More than 70% of flowering plants have undergone polyploidization at some point in their evolutionary history^32^. Allopolyploid genomes are derived from combinations of genomes from different species, and a critically important period in this process is immediately after allopolyploid formation, when the two distinct genomes are first brought into contact, thereby requiring a diverse array of genic, genomic, and physiological accommodations^32^. Some genomic changes arise immediately with the onset of polyploidy, whereas others have occurred over several generations. Extensive molecular evidence indicates that polyploid genomes show dynamic and pervasive changes in their DNA sequences and gene expression profiles, probably as a response of inter-genomic interactions during the so-called genomic shock (i.e. the release of genome-wide constraints on gene expression and sequence organization)^42^.

Liu et al. used an AFLP analysis of over 22,000 fragments from eight synthetic hexaploids and one tetraploid progeny cotton and found additive patterns for nearly all AFLP fragments examined^33^. However, the resolution of AFLP analysis was not enough to detect large-scale structural variations. In the present study, our reference-grade genome assemblies allowed us to perform direct comparisons between allotetraploid upland cotton and their likely ancestors. Many of those changes in cotton are likely caused by reciprocal translocations, non-reciprocal exchanges, and inversions (Fig. 1a, b). And most of the rearrangements between allopolyploid cotton and their diploid ancestors are shared by both TM-1 and ZM24, indicating that these variations occurred after genome doubling and pre-divergence of the two cultivars. More rearrangements were observed in the A_t_ subgenome than those in the D_t_ subgenome, indicating that the larger A_t_ subgenome has been acquiring inversions and translocation more quickly than the smaller D_t_ subgenome. A similar situation was also observed through comparison of rice, sorghum, and *Ae. Tauschii*: the numbers of rearrangements mirrored the genome sizes^43^.

The recent resequencing of a core collection of upland cotton accessions revealed large genomic regions that feature a very low extent of genetic polymorphisms^40^. However, owing to the short-read sequencing methods used to generate the assemblies to which the accession sequence data was mapped, the data resource generated in that study did not facilitate the identification of large rearranged regions, and was not able to detect the very influential SV1 and SV3 inversions that we identified in our intra-species analyses of the higher resolution assemblies of the TM-1 and ZM24 cotton varieties.

Chromosomal rearrangements such as inversions and translocations have long been thought to play a critical role in adaptation and speciation^43^. However, the ecological traits linked to chromosome inversions have been poorly studied^43,44^. Lowry et al.^44^ discovered that a chromosomal inversion polymorphism is geographically widespread, and their results demonstrated the contribution of an inversion to adaptation, an annual/perennial life-history shift, and multiple reproductive isolating barriers. Ayala et al.^45^ studied the roles of chromosome inversions in the adaptation of the four major African malaria mosquitoes to local environments in Africa and found that most inversions are actively involved in processes of local adaptation. They also found that some inversions exhibited similar geographical patterns and ecological requirements among the four mosquito species, providing evidence for parallel evolution. Fransz et al.^46^ studied the roles of *Arabidopsis* paracentric inversion in fecundity under drought and noted a strong association. Further, they found linkage disequilibrium between the inverted region and the early flowering *Col-FRIGIDA* allele, which may have contributed to the maintenance and/or spread of the inversion in the population. In Arabidopsis, an inversion on Chr04 generated the Col-0 ecotype, and newly Col-0-like alleles have become widely distributed in the *Arabidopsis* population^39^. We found that the ZM24-like allele of the inversions on A08 was more widely distributed among the accessions of a large germplasm diversity panel, which may indicate that the ZM24-like group carries selective advantages that somehow enable a stronger capacity to adapt to a larger cultivars of ecological environments; however, given that there are only a very small number of accessions with TM-1-like alleles for A08 for a given geographical region, it is difficult to predict which traits might influence this capacity.

Using PacBio long read sequencing technology, the total assembly of TM-1 was 2286 Mb, which is slight longer than the recently published upland cotton genome (2282 Mb). Compared with the recently published TM-1 genome, the sequence contiguities of our assemblies were higher; note for example that our TM-1 contig N50 increased to 4760 Kb (1891 Kb of TM-1_HAU)^12^. More than 99.4% of the TM-1 and ZM24 sequences were anchored onto the 26 pseudochromomses, a value slightly higher than that of TM-1_HAU (98.94%). Using Hi-C technology, 97.5% TM-1 and 93.7% of the ZM24 anchoring sequence were oriented onto the 26 pseudochromomses, respectively, among which our TM-1 anchoring ratio was slightly higher than that of the recently published genome (TM-1_HAU, 96.16%), indicating that all three assemblies are nearly complete. Genomic analysis showed that the two TM-1 assemblies share high collinearity across all chromosomes except for D08 chromosome, suggesting that most of the contigs or scaffolds are properly anchored and oriented. Hi-C heatmaps further supported that our assemblies were correct for D08 (Supplementary Fig. 5), highlighting that Hi-C is a powerful tool technique for the identification of large-scale variations in genomes. Further, when we compared our TM-1 D08 sequence with *G. raimondii*_D08, TM-1_ZJU_D08, Hai7124_ZJU_D08, and TM-1_NAU_D08, we found that our TM-1 D08 sequence showed strong collinearities with each of these assemblies, again supporting the quality of our assembly.

Approximately 99% of the TM-1_ICR and ZM24 D_t_ subgenomes are located in one-to-one blocks with TM-1_HAU D_t_, and ~ 98% of TM-1_ICR and ZM24 A_t_ are located in one-to-one blocks with TM-1_HAU A_t,_ thus mutually verifying the high qualities of all of the assemblies. Large-scale inversions between TM-1 and *G. barbadense* have been reported for 14 chromosomes, including A02, A03, A06, A08, A09, A11, A12, D03, D04, D05, D06, D07, D08, and D12^12^, and we also observed these inversions. We also discovered large-scale inversions between TM-1 and *G.barbadense* and between TM-1 and *G. arboreum* within the same regions on A06; so no obvious inversions between *G. barbadense* and *G. arboreum* were observed in our data, supporting that these A06 inversions are unique to upland cotton and occurred after polyploidization. We also observed large-scale inversions between TM-1 and *G. arboreum* in A07, A10, A11, and A13; however, no large-scale inversions were found within these chromosomes between TM-1 and *G. barbadesne*, indicating that these inversions apparently occurred in the ancestor of upland cotton and *G. barbadense*. Similarly, we found no large-scale translocations between TM-1 and *G. barbadense*, indicating that the translocations between the A_t_ subgenome and the A_2_ genome occurred in the ancestor of upland cotton and *G. barbadense*.

Note that our assemblies include about 3500 more gene models, and our assemblies covering ~96% (67,160 of 70,199) of the predicted genes from the recently published genome. BUSCO evaluation showed that more embryophyta genes were found in both of our assemblies than in the recently published one^12^, suggesting that our assemblies are more complete. Most of the variations between *G. hirsutum* and *G. barbadense* are shared by three upland cotton references, again indicating that all three references are of high quality. Further, the anchoring ratio and contiguity of *G. barbadense* is high, indicating that *G. barbadense* has a high assembly quality. In sum, the new upland cotton assemblies generated in our study represent truly substantial improvements in quality and resolution compared to previously available assemblies. Further, our comparisons of current genome assemblies with previous draft genomes and with the genomes of the diploid progenitors of upland cotton show the improved assembly quality. These comprehensive assemblies facilitated discoveries about how inversions on A08 have apparently affected the propensity for meiotic recombination and thus lowered the genetic diversity in inversion regions in cotton populations. Given the low genetic diversity in cotton relative to many other crop species and the attendant challenges this brings cotton breeders, the genomics resources developed in our study should substantially support the basic and applied efforts of the worldwide cotton research community.

## Methods

### Plant materials

*G. hirsutum* L. acc. TM-1 and ZM24 were selected for sequencing because of their important positions in the fields of cotton breeding and genetic research. The plants were grown in a greenhouse for 30 days, and the young leaves from a single plant were harvested and frozen immediately in liquid nitrogen for extraction of genomic DNA. Note that we also conducted a cross between ZM24 (maternal parent) and TM-1 (paternal parent) and used the single-seed descendant method to generate RILs (Supplementary Fig. 34). A total of 181 RILs were used for genetic map construction in this study.

### PacBio sequencing

An improved CTAB method was used to extract the genomic DNA of TM-1 and ZM24. The modified CTAB extraction buffer included 0.1 M Tris-HCl, 0.02 M EDTA, 1.4 M NaCl, 3% (w/v) CTAB and 5% (w/v) PVP40. Beta-mercaptoethanol was added to the CTAB extraction buffer to ensure DNA integrity and quality. Gnomic DNA was sheared by a g-TUBE device (Covaris) using the 20 Kb setting. Sheared DNA was purified and concentrated with AmpureXP beads (Agencourt) and further used for Single-Molecule Real Time (SMRT) bell preparation according to manufacturer’s protocol (Pacific Biosciences; 20-Kb template preparation using BluePippin size selection (Sagescience). Size-selected and isolated SMRT bell fractions were purified using AmpureXP beads (Beckman Coulter, Inc.) and finally these purified SMRT bells were used for primer (V3) and polymerase (2.0) binding according to manufacturer’s binding calculator (Pacific Biosciences). Single-molecule sequencing was done on a PacBio Sequel system and yielded a total of 19,951,021 and 14,126,960 filtered subreads with average lengths of 10,248 bp and 8799 bp for TM-1 and ZM24, respectively. Finally, only PacBio subreads equal or longer than 500 bp were used for generating the two genome assemblies.

Total RNA from multiple tissues (leaf, flower, callus, ovule, cotton-fiber, torpedo-shape embryo, and cotyledonary embryo) was isolated using Trizol reagent (Invitrogen) followed by treatment with RNase-free DNase I (Promega, USA) according to the manufacturers’ protocols. The quantity and quality of RNA was checked using a Nanodrop ND-1000 spectrophotometer (NanoDrop Technologies, USA) and an Agilent 2100 Bioanalyzer. cDNA was synthesized using SMARTer PCR cDNA Synthesis Kits, optimized for preparing full-length cDNA (Takara, Japan). Size fractionation and selection (1–2, 2–3, and >3 Kb) were performed using the BluePippin™ Size Selection System (Sage Science, USA). The SMRT bell libraries were constructed with Pacific Biosciences DNA Template Prep Kits v.2.0. SMRT sequencing was then performed on the Pacific Bioscience Sequel platform using the provided protocol.

### Illumina sequencing

We constructed libraries with a 270 bp insert fragment for TM-1 and ZM24 following the manufacturer’s protocols (Illumina): (i) genomic DNA was fragmented by nebulization with compressed nitrogen gas; (ii) DNA ends were polished, and an adenine was added to the ends of the fragments; (iii) DNA adaptors (Illumina) with a single T overhang at the 3′ end were ligated to the DNA fragments above; (iv) the ligation products were run on 2% agarose gels, and the bands corresponding to each insert size were excised. These libraries were sequenced on a HiSeq 2500 system with a PE150 strategy following the manufacturer’s instructions (Illumina). For the raw Illumina reads, sequencing adaptors were removed; contaminated reads (mitochondrial, bacterial, and viral sequences, etc.) were screened by alignment to NCBI-NR database using BWA v0.7.13^47^ with default parameters; FastUniq^48^ v1.12 was used to remove the duplicated read pairs; the low-quality reads were filtered satisfying the following conditions: (1) reads with ≥10% unidentified nucleotides (N), (2) reads with >10 nucleotides aligned to the adapter, allowing ≤10% mismatches, (3) reads with >50% bases having Phred quality <5. Finally, we generated a total of 69.95 Gb (~29.64×) and 117.54 Gb (~49.63×) clean Illumina reads for TM-1 and ZM24, respectively. For the RIL population, a total of 765.04 Gb clean reads were obtained, of which the parents and RILs individuals occupied 153.94 and 611.1 Gb, respectively. Average sequencing depths of sequenced loci per parent and per progeny were 33.32- and 1.46-fold, respectively.

### De novo assembly

The assembly was conducted using a Canu-based pipeline^49^ with the following procedures: longer seed reads were selected with the settings genomeSize = 600000000 and corOutCoverage = 35; raw reads overlapping was detected through a high sensitive overlapper MHAP (mhap-2.1.2, option corMhapSensitivity = low/normal/high), and an error correction was performed through Falcon sense method (option correctedErrorRate = 0.025); error-corrected reads were trimmed of unsupported bases and hairpin adapters in order to reach their longest supporting range with the default parameters, and then the draft assemblies of ZM24 and TM-1 were generated using the top 80% longest trimmed reads. The raw Illumina reads were filtered as mentioned in the Illumina sequencing section. Finally, the clean reads from the same sequencing individuals were integrated to correct the SNPs and InDels in the draft assemblies using Pilon v1.22^50^ with the parameters -mindepth 10 –changes –threads 4 –fix bases.

The completeness of the two assemblies were evaluated by mapping 1440 Benchmarking Universal Single-Copy Orthologs to the genomes using BUSCO v3.0.2b^25^, which showed that 1420 (98.61%) and 1419 (98.54%) complete BUSCOs and 5 (0.35%) and 7 (0.74%) fragmented BUSCOs in the TM-1 and ZM24 assemblies, respectively.

### Hi-C sequencing data

According to the Hi-C procedure, nuclear DNA from leaves of TM-1 and ZM24 individuals were cross-linked and then cut with a restriction enzyme, leaving pairs of distally located but physically interacting DNA molecules. The sticky ends of these digested fragments were biotinylated and then ligated to each other to form chimeric circles. Biotinylated circles-chimeras of the physically associated DNA molecules from the original cross-linking were enriched, sheared, and sequenced. After reads filtering, we obtained 476.35 million and 324.20 million valid interaction pairs for the chromosome-level assembly of ZM24 and TM-1, respectively. The contigs within the assemblies were separately broken into fragments with a length of 50 Kb and were then clustered using LACHESIS software^51^ using valid interaction read pairs; finally, 699 and 1497 contigs with total lengths of 2.23 and 2.15 Gb were anchored and oriented to their respective 26 chromosome-level groups of TM-1 and ZM24. In addition, a published genetic map with 4049 bins between *G. hirsutum* and *G. barbadense* was aligned to both genomes after Hi-C directed assembly (Supplementary Fig. 7–10)^24^.

### Repeated sequence prediction

The repeat components in the two assemblies were first estimated by building a de novo repeat library employing the programs LTR-FINDER^52^, MITE-Hunter^53^, RepeatScout v1.0.5^54^ and PILER-DF^55^, and the output results were merged together and classified using PASTEClassifier v1.0^56^. This de novo constructed database, together with the Repbase database v20.01, was both used to create the final repeat library. Repeat sequences in TM-1 and ZM24 were finally identified and classified using RepeatMasker program v4.0.6^57^. The LTR family classification criterion was defined by which 5′LTR sequences of the same family would share at least 80% identity over at least 80% of their length. The repeated sequences occupy 73.68% (1.69 Gb) of the TM-1 assembly and 72.14% (1.67 Gb) of ZM24 assembly, respectively, of which *Gypsy* retrotransposons account for more than 40% in both assemblies. The expansion history of TEs was estimated by computing the divergence of the TEs copies in their respective assembly with the corresponding consensus sequence in the repeat library using Kimura distance^27^; we then calculated the percentage of TEs at different divergence levels. All intact LTRs and solo LTRs were used to calculate the insert time, with the formula time = K/2r (where K is the distance between all alignment pairs and r is the rate of nucleotide substitution). The r value^8^ was set to 7 × 10^–9^, and K was calculated with the distmat program, implemented in the EMBOSS^28^ package with the Kimura two-parameter model.

### Protein-coding gene prediction

We used de novo, protein homology, and Iso-Seq^58^ approaches for the protein-coding genes prediction. In detail, Genscan v1.0^59^, Augustus v2.5.5^60^, GlimmerHMM v3.0.1^61^, GeneID v1.3^62^ and SNAP^62^ were used for de novo gene prediction; GeMoMa v1.4.2^63^ was used to align the homologous peptides from *Arabidopsis thaliana*, *Oryza sativa* L. ssp. japonica, *G. hirsutum* (NAU), and *G. raimondii* (JGI) to our assemblies; the consensus isoforms derived from PacBio long cDNA reads were aligned to the repeat-masked assemblies using BLAT^64^, and subsequently the gene structures of BLAT alignment results were modeled using PASA^65^. Finally, these consensus gene models were generated by integrating the de novo predictions, protein alignments and transcripts data using EVidenceModeler. In total, 73,624 and 73,707 genes were predicted for TM-1 and ZM24, respectively. Annotations of the predicted genes were performed by blasting their sequences against a series of nucleotide and protein sequence databases, including COG, KEGG^66^, NCBI-NR (version May 2013) and Swiss-Prot^67^, using an *e* value cutoff of 1e-5. Gene ontology (GO) terms for each gene were assigned using Blast2GO based on NCBI databases.

### Paralog analysis for TM-1 and ZM24

The all-against-all BLASTP^68^ method (*e* value < 1e-5) was used to detect paralogous genes in TM-1 and ZM24. Homologous blocks were then detected using MCScanX v1.1^69^, requiring at least five collinear gene pairs within one block and fewer than 25 intervening genes.

### Identification of chromosomal structural variations

We used MUMmer v3.1^70^ (http://mummer.sourceforge.net/) to identify the SNPs and InDel between A_t_/D_t_ subgenome of TM-1 and ZM24 and their respective diploid ancestor *G. arboreum* (A_2_) and *G. raimondii* (D_5_), as well as between A_t_/D_t_ subgenome of TM-1 and their respective counterpart subgenomes of ZM24 using the following procedures: (1) each query genome was aligned with the corresponding reference genome using the nucmer utility under the parameters –mum. (2) the delta-filter utility was used to filter mapping noise and determine the one-to-one alignment blocks with parameters −1 -r -q, and only those SNPs and single-base InDel satisfying where the parameter Buffering was set to 20 were retained. (3) the show-diff utility was used to obtain the GAP type (gap between two mutually consistent alignments) for calling the InDels with length of longer than one base but shorter than 100 bp.

The detection of inversions and translocations was completed by filtering the nucmer outputs using delta-filter utility through two sets of parameters settings: -i 90 -1 -r -q and -i 90 -g -r –q, respectively, where -1 was used to obtain one-to-one alignment blocks allowing for rearrangements and -g was used to obtain co-linear region which is the global alignment but not allowing rearrangements. The alignment blocks with those translocated regions (the translocated regions involving the global alignments under the -g parameter) excluded were deemed to be allelic regions. The non-allelic regions were obtained by subtracting the allelic regions from the alignment blocks produced under the -1 parameter setting. These non-allelic regions were finally defined as inversions or translocations depending on their locations and orientations adjacent to their neighboring blocks. The output of nucmer was filtered by delta-filtering using a minimum identity of 90% for *G. arboreum* and 95% for *G. hirsutum*. This defined the allelic regions, and thereby defined the syntenic backbone of the chromosomes. Homology blocks that didn’t belong to these allelic regions, were defined as inversions or translacotions depending on their location and orientation to their neighboring blocks. We found that there were 13,819 rearrangements (translocations and inversions) between TM-1 A_t_ and A_2_, 7492 rearrangements (translocations and inversions) between TM-1 D_t_ and D_5_. Finally, neighboring blocks, which were assigned to the same non-allelic region, were joined into one rearranged region, so we found a total of 64 translocations and 54 inversions for the interspecific comparisons.

The show-diff utility was used to select for unaligned regions, which are categorized as GAP (gap between two mutually consistent alignments), DUP (inserted duplication), BRK (other inserted sequence), JMP (rearrangement), INV (rearrangement with inversion), and SEQ (rearrangement with another sequence). These unaligned regions were retained as potential unique presence regions in TM-1 or ZM24, respectively, and then were aligned to their respective genome using blastn (*e* value < 1e-5). The final unique presence regions in TM-1 or ZM24 were obtained by filtering those regions with coverage >50% and identity >90%. The border sequences of left and right breakpoints of SV1 and SV3 were extracted from both TM-1 and ZM24. Primers for cloning the fragments containing each breakpoint are shown in Supplementary Table 17. The PCR products were evaluated with Sanger sequencing.

### Introgression analysis

The A_t_ subgenome and D_t_ subgenome of TM-1 and ZM24 was separately divided into continuous bins with each bin having a length of 5 Kb, and these bins were then blasted against both *G. arboreum* and *G. raimondii* with the parameters: -max_target_seqs 1 -*e* value 1e-05 -perc_identity 90 -word_size 50. Only those bins covering 70% of target regions in length were retained for further analysis. If a bin has a ≥1.5-fold coverage ratio of A_t_-D_5_/A_t_-A_2_ or D_t_-A_2_/D_t_-D_5_ with its targets, then introgression is considered to have occurred in this bin.

### Orthologous genes and structural variations

The one-to-one orthologous genes of A_t_/D_t_ subgenomes in TM-1 and ZM24 were respectively identified using Inparanoid v4.1^71^. In some cases, for one ortholog of A_t_/D_t_ subgenome in TM-1, no matching counterpart was not detected in ZM24, then the GeMoMa software was used to verify its absence for the sake of avoiding the prediction error, and *vice versa*. In total, we obtained 36,040 orthologous gene pairs for A_t_ subgenome and 35,836 D_t_ subgenome, respectively, of which 34,634 and 34,518 could be anchored to pseudochromosomes. Subsequently, we analyzed the genetic variations in each of the orthologous gene pairs according to the following procedures: (1) the longest transcript was select candidate within each gene loci; (2) the 2 Kb upstream and downstream of gene coding regions were extracted and aligned to their counterpart genomic regions with BWA; (3) the orthologous genes with an equal number of exons, but allowing the existence of nonsynonymous variation and deletion of some codons, were defined as structurally conserved genes; (4) orthologous genes with equal numbers of exons but with changeable length, or with alteration of splice sites, or with frameshift mutations, were defined as large-effect variations; (5) the remaining orthologous genes were considered as genes with large structural variations. Furthermore, these orthologous genes carrying large structural variations were categorized into syntenic orthologs (orthologous gene pairs simultaneously locate on a pair of syntenic block) and nonsyntenic orthologs (orthologous gene pair locate on nonsyntenic blocks and only one ortholog of orthologous gene pair locate on a pair of syntenic block).

### SNPs and InDels in a cotton germplasm diversity panel

All reads were processed for quality control and filtered using Seqtk (https://github.com/lh3/seqtk). Then, the high-quality paired-end reads were mapped onto the TM-1 and ZM24 using the BWA. The mapping results were processed by sorting and duplicate mark using functions implemented in SAMtools^72^ and Picard (http://broadinstitute.github.io/picard/). Realigner Target Creator and InDel-Realigner in the Genome Analysis Toolkit 4 (GATK4)^73^ were used to realign InDels, and Unified Genotyper was used to identify SNP across the 421 accessions using the default parameters (note that the sequence data for these accessions included previously published information and the new sequences for TM-1 and ZM24). Data filtration was performed following the best practices workflow developed by the GATK team. SNPs with minor allele frequency (MAF) < 5% were excluded from the genotype datasets of all the accessions. For each accession, sequencing coverage and depth were calculated using the DepthOfCoverage module in GATK. Functional annotation of variants was completed using SnpEff v4.3T^74^.

### Population differentiation and haplotype diversity analysis

A phylogenetic tree for 421 cotton accessions was constructed based on 315,868 SNPs (MAF ≥ 0.05, Heterozygosity ≤ 10% and SNP max-missing rate ≤ 10%) using a neighbor-joining method with 1000 bootstraps in FastTree^75^. Principal component analysis (PCA) was performed using the EIGENSOFT/SmartPCA algorithm^76^. The population differentiation (F_*ST*_) analysis based on 1000 Kb sliding windows in 100 Kb steps was conducted using the PopGen package ((http://cran.r-project.org/web/packages/popgen/index.html)) in BioPerl. The genetic diversity (π) analysis based on 1000 Kb sliding windows (10 Kb step size) was conducted using vcftools (version 0.1.12b). Haplotype diversity was calculated using all SNPs with a minor allele frequency of at least 5%. Haplotypes, defined previously as clusters of identical genotypes in a 10 Kb window^39^, were thusly analyzed (mean haplotype diversity was evaluated in a 1000 Kb sliding window with a 100 Kb step size)^77^. We compared the SNP locations of the ZM24-like groups with those from TM-1 like group. If the SNP co-occurred in both groups, it was deemed as common SNP. If the SNP only existed in one of the groups, it was deemed as private SNP. We randomly selected an equal number of accessions for the TM-1-like and ZM24-like groups and analyzed the common and private SNPs.

### Genotyping of inversion breakpoints

To characterize inversion alleles throughout a globally representative diversity panel for upland cotton, we used publically available short-read data^40^. A total of 419 accessions met our criteria for sufficient sequencing quality. Short reads were aligned with the newly assembled TM-1 using BWA. For each of the inversion breakpoints, we made determined whether or not the mate-pair reads spanned the TM-1-like breakpoints. This generated two clearly separated groups of mate-pair reads, including TM-1-like and ZM24-like supported groups, which allowed us to characterize each of the accessions for inversion alleles using the confident assignments of the TM-1 and ZM24 haplotypes, respectively. For controls in this analysis, we included whole genome sequencing short-read data for TM-1 and ZM24, which were assigned to their respective inversion alleles.

### Genetic map construction

The sequencing data for the RILs were aligned to the ZM24 genome using SOAP^78^. In total, 786,241 high-quality SNP markers (at least 4× coverage) were identified and were then combined into 4482 bins to construct a genetic map using HighMap software^79^. The final map spanned 3,370.91 cM across 26 linkage groups, with a mean marker density of 0.75 cM per bin. The cumulative distances (in centimorgans) were calculated within each 1 Mb sliding window (with a 0.5 Mb step between each window) along the A08 chromosome in ZM24.

### Reporting summary

Further information on research design is available in the Nature Research Reporting Summary linked to this article.

## Supplementary information

Supplementary information

Peer Review

Reporting Summary

Description of Additional Supplementary Files

Supplementary Data 1

Supplementary Data 2

Supplementary Data 3

Supplementary Data 4

Supplementary Data 5

Supplementary Data 6

Supplementary Data 7

Supplementary Data 8

Supplementary Data 9

Source Data

## Data Availability

Data generated in the present study have been deposited with NCBI as study PRJNA503326. Raw data (PacBio, Hi-C, and Isoform seq) have been deposited in the Sequence Read Archive (SRA) under study accession number SRX5002527 -SRX5002543. The RNA-seq data of TM-1 and ZM24 calli samples are available under the SRA accession numbers SRX5438542 -SRX5438553. The raw data for the RILs are available under SRA accession numbers SRX5087375 -SRX5087519. Genome assembly and annotation data have been deposited at Dryad Digital Repository [https://datadryad.org/resource/doi:10.5061/dryad.tg557hc]. Genome assemblies and genes annotations, as well as gff files for gene models can also be downloaded from [ftp://bioinfo.ayit.edu.cn/downloads/, https://github.com/gitmalm/Genome-data-of-Gossypium-hirsutum/]. Data supporting the findings of this work are available within the paper and its Supplementary Information files. A reporting summary for this Article is available as a Supplementary Information file. The datasets generated and analyzed during the current study are available from the corresponding author on reasonable request. The source data underlying Fig. 1a, b, Fig. 2, and Fig. 3e–h, as well as Supplementary Figs. 11, 12, 16, 20, 21, 22h, 26d, and 31–33 are provided in a Source Data file.
