# Supplementary material for: Extensive intraspecific gene order and gene structural variations in upland cotton cultivars
Source: Nat Commun. 2019 Jul 5;10:2989. doi: 10.1038/s41467-019-10820-x (PMC6611876; doi:10.1038/s41467-019-10820-x)
Supplement: Supplementary file 1 — Supplementary information [file 41467_2019_10820_MOESM1_ESM.pdf]

Extensive intraspecific gene order and gene structural variations in upland cotton cultivars

Yang et.al

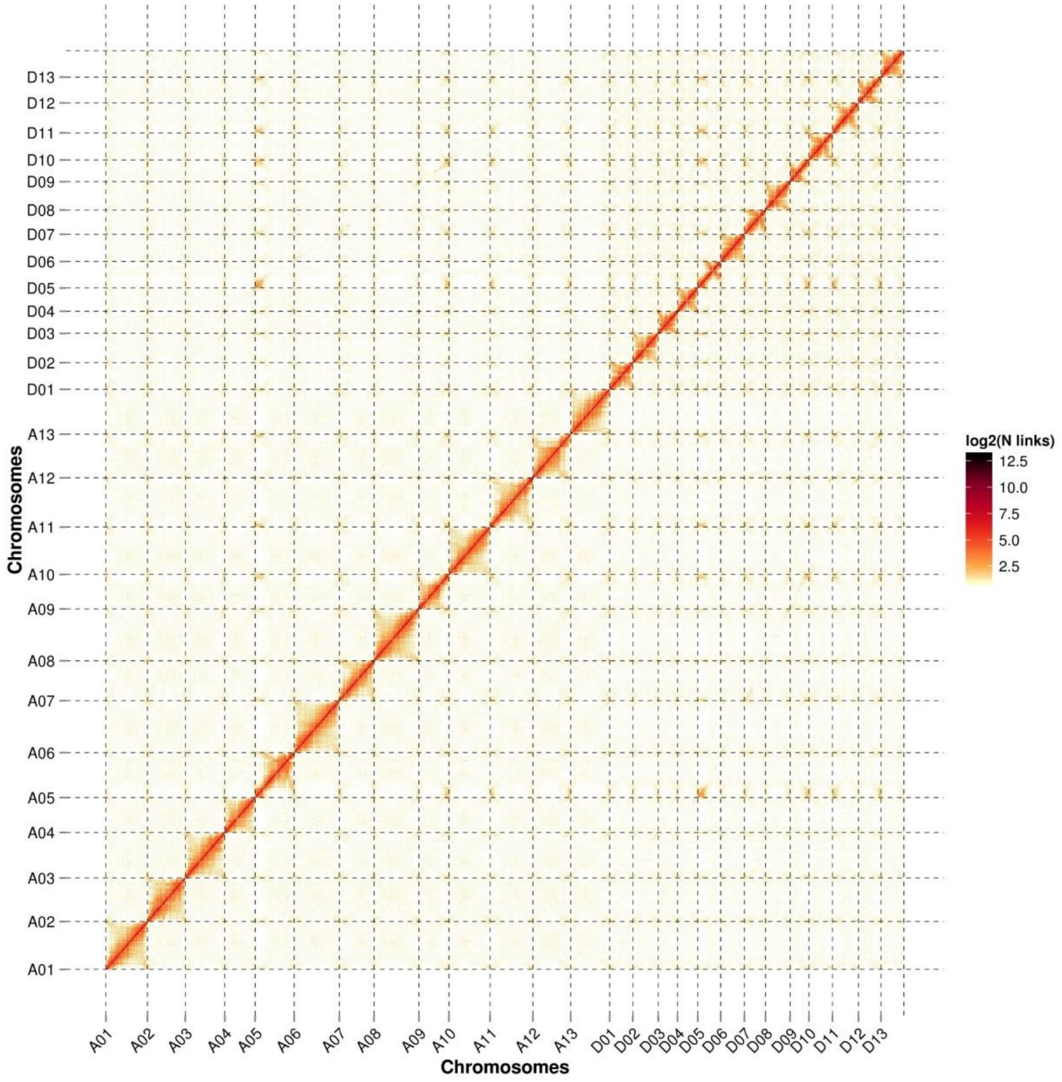

Supplementary Figure 1. TM-1 Hi-C contact data mapped on the TM-1 genome. Strong signals were observed on diagonal region, indicating that the contigs were accurately oriented on the pseudochromosomes.

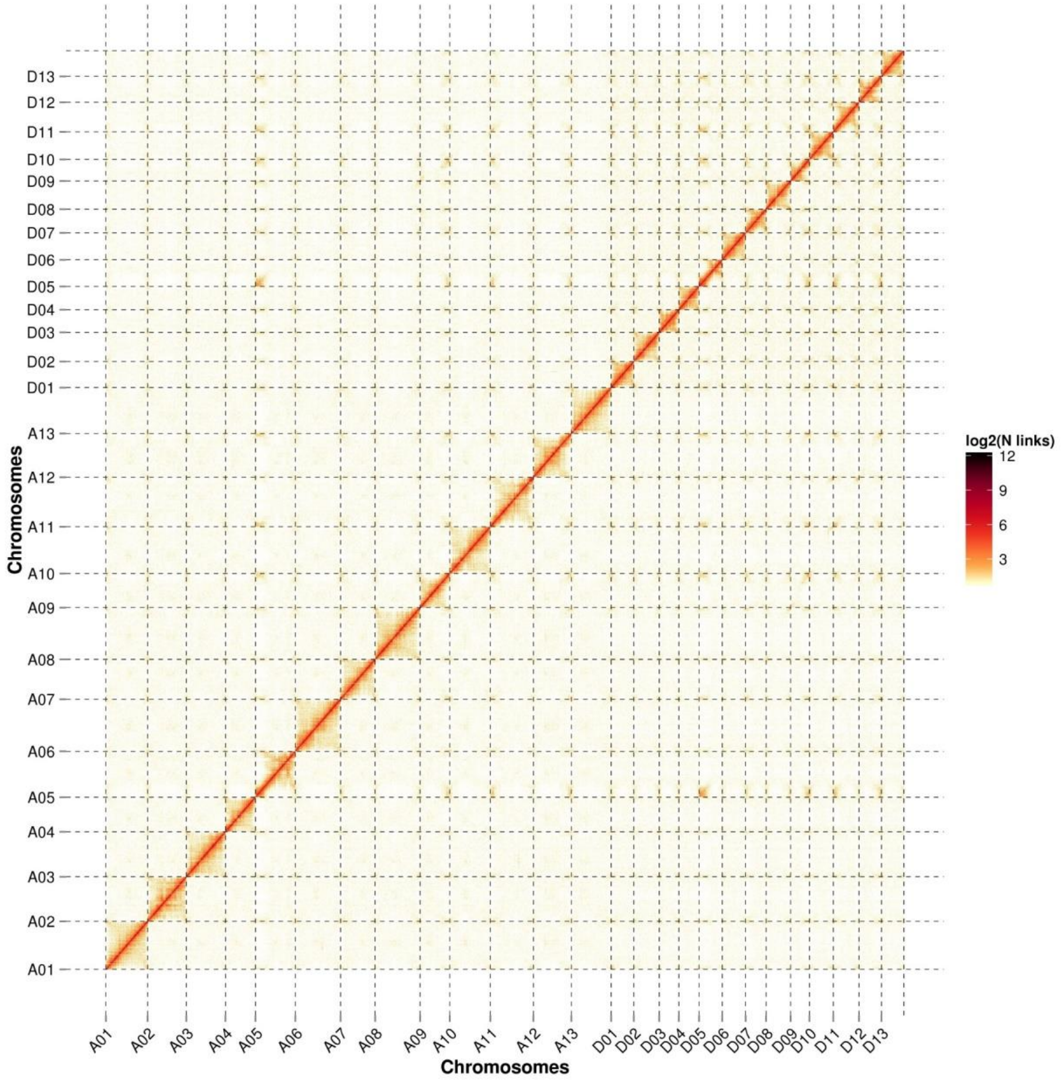

Supplementary Figure 2. ZM24 Hi-C contact data mapped on the ZM24 genome. Strong signals were observed on diagonal region, indicating that the contigs were accurately oriented on the pseudochromosomes.

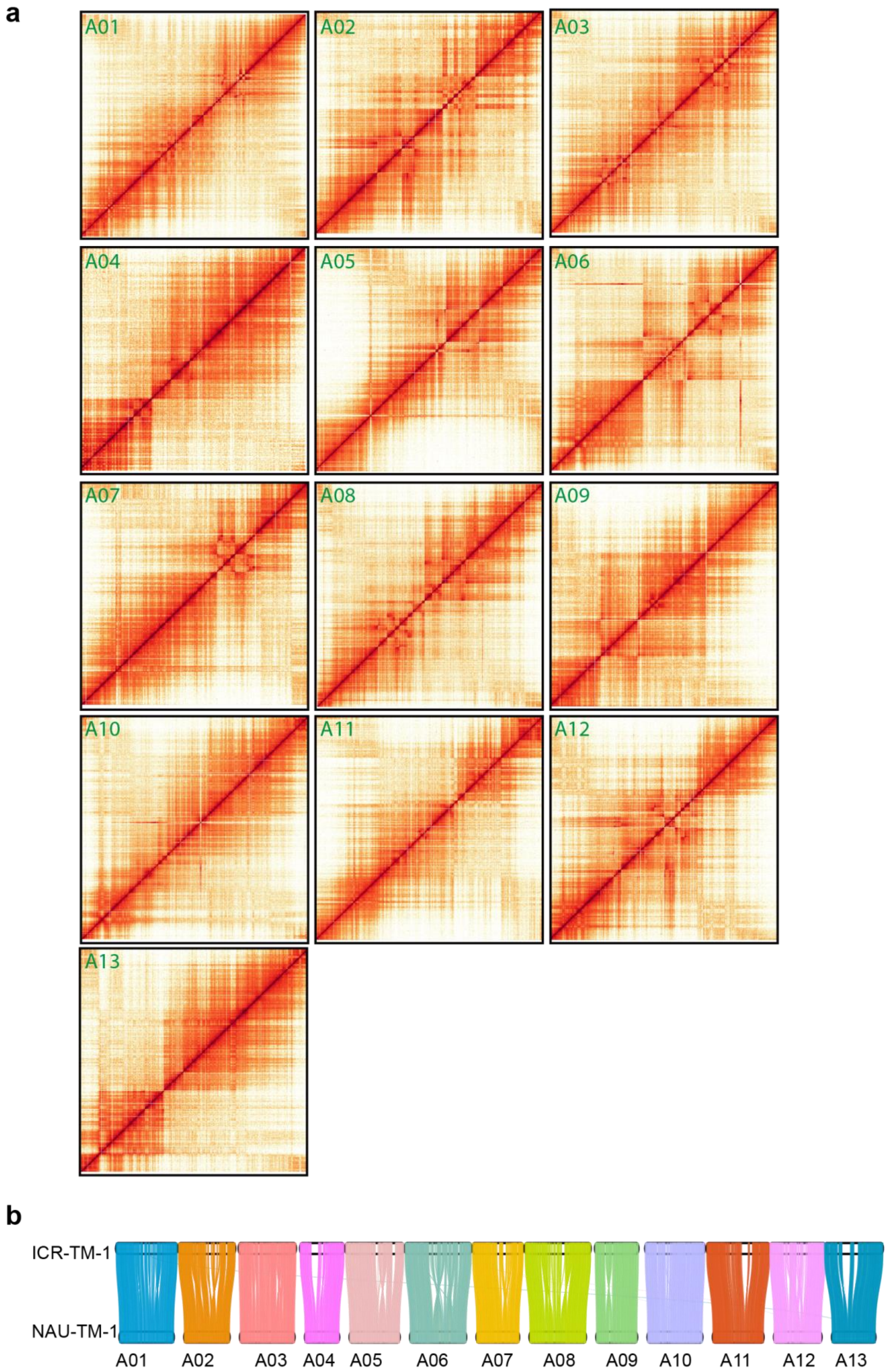

Supplementary Figure 3. Genomic comparison between TM-1 A_t subgenome in this study with the previously published sequence (NAU). a, The TM-1 Hi-C data mapping against the A_t subgenome of TM-1 (NAU). The discrete interaction shows the likely incorrect assembly regions in the previously published TM-1 draft genome (NAU)[1]. The dark red dots represent high probability of interaction and

the light yellow dots represent low probability of interaction. b, Genome alignment of TM-1 in this study with the previously published sequence (NAU). The upper panel represents the A_t subgenome of TM-1 in this study and the lower panel represents the A_t subgenome of the previous genome (NAU). Those regions which are not covered by colored lines in each chromosome indicate the missing assemblies in the previously published genome (NAU).

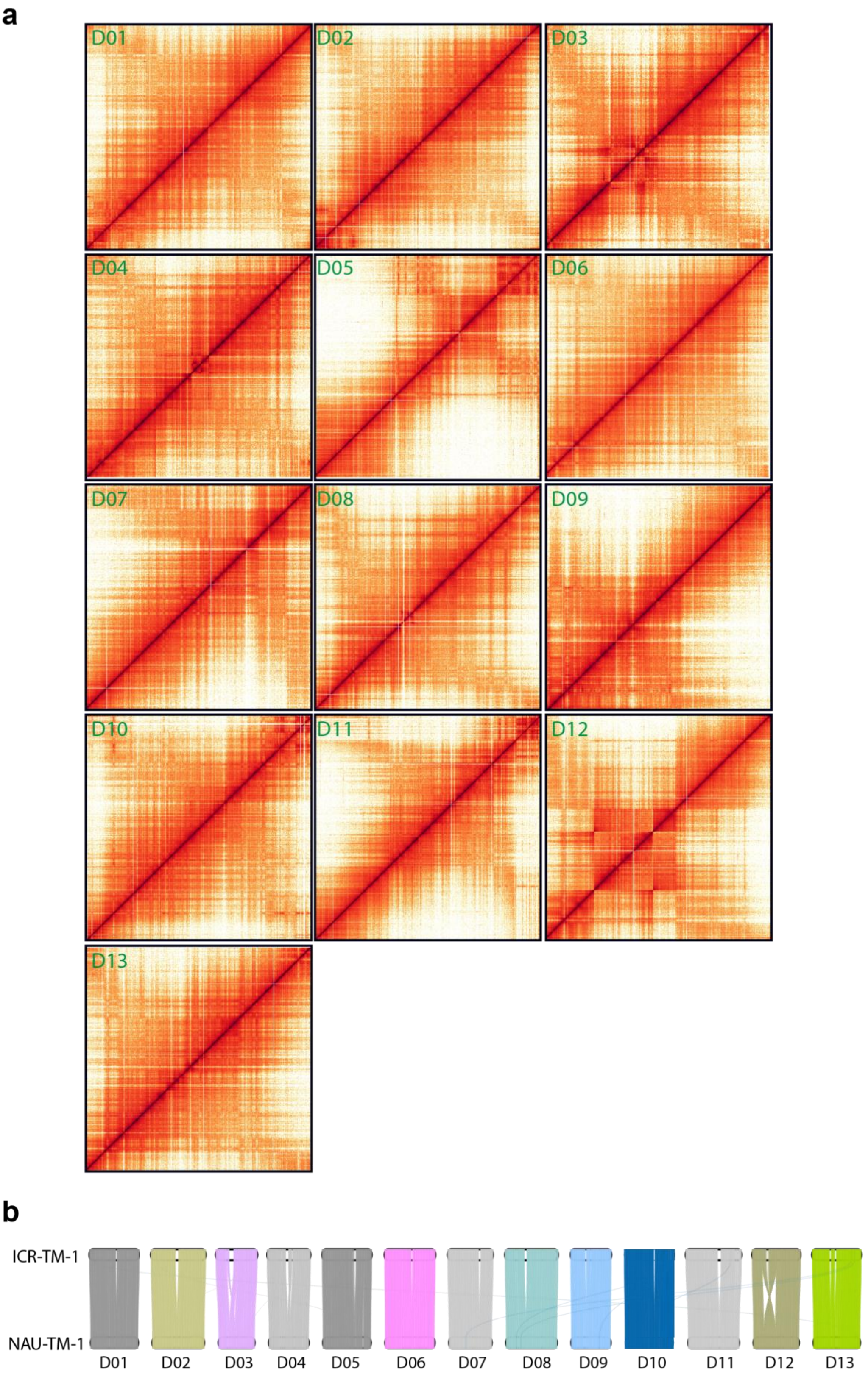

Supplementary Figure 4. Genomic comparison between the TM-1 Dt subgenome in this study with the previously published sequence (NAU). a, The TM-1 Hi-C data mapping against the D_t subgenome of TM-1 (NAU). The discrete interaction shows the likely incorrect assembly regions in the previously published TM-1 draft genome (NAU). The dark red dots represent a high probability of interaction and

light yellow represent a low probability of interaction. b, Genome alignment of TM-1 in this study with the previously published sequence (NAU). The upper panel represents the D_t subgenome of TM-1 in this study and the lower panel represents the D_t subgenome of the previous genome (NAU). Those regions which are not covered by colored lines in each chromosome indicate the missing assemblies in the previously published genome (NAU).

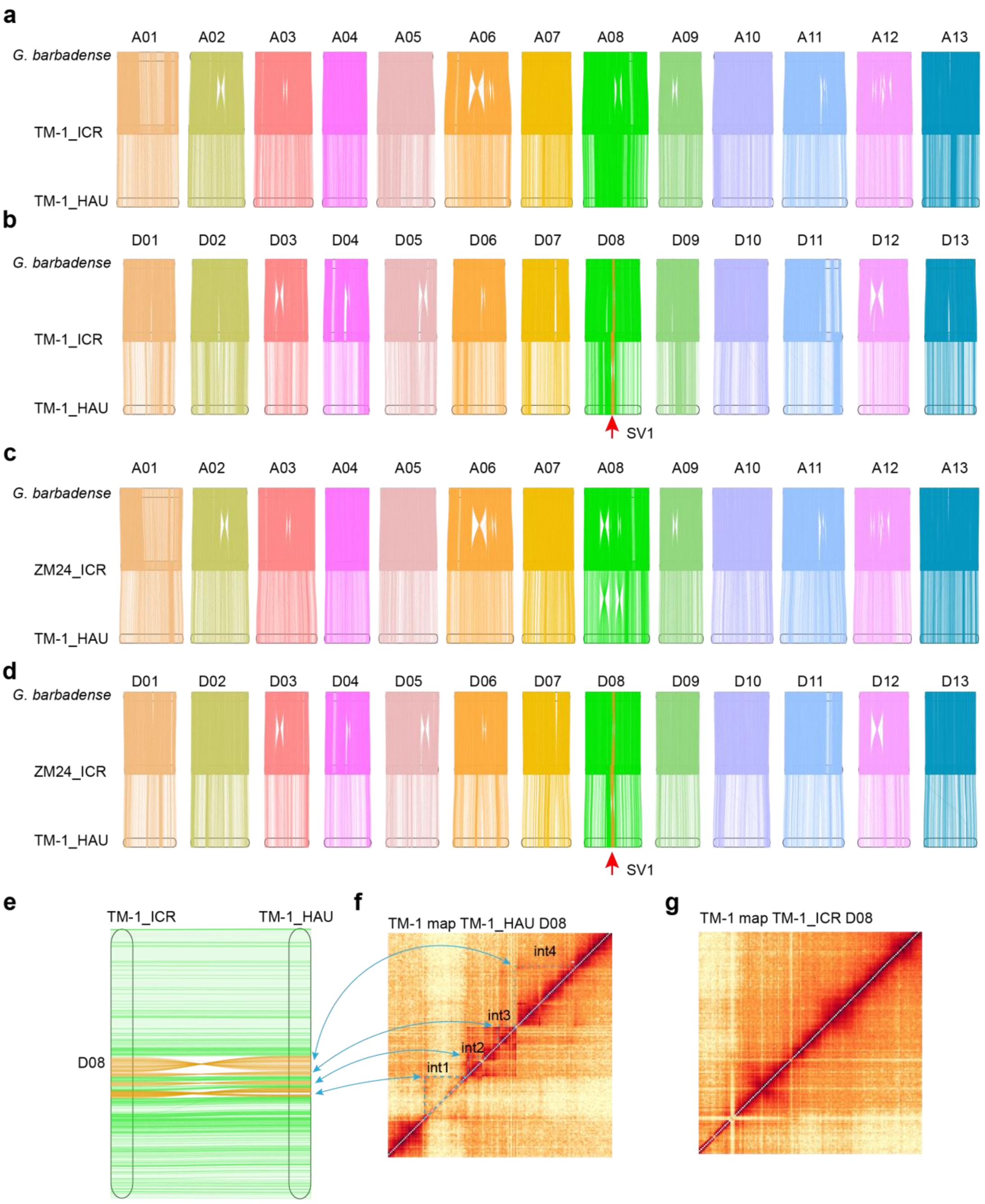

Supplementary Figure 5. Genomic comparison between the two assemblies in this study with the recently published upland cotton sequence (TM-1_HAU) and *G. barbadense*. a, Genome alignment of TM-1A_t subgenome in this study (TM-1_ICR) with the TM-1_HAU and *G. barbadense*. b, Genome alignment of TM-1_ICR D_t subgenome with the TM-1_HUA D_t subgenome and *G. barbadense*. The red arrow indicates the inverted region (SV1) between TM-1_ICR D08 and TM-1_HAU_D08. c, Genome alignment of the ZM24 A_t subgenome with the TM-1_HAU and *G. barbadense* A_t subgenomes. d, Genome alignment of the ZM24 D_t subgenome with the TM-1_HAU and *G. barbadense* D_t subgenomes. The red arrow indicates the inverted region (SV1) between the ZM24_ICR D08 and TM-1_HAU_D08. e, Chromosome alignment of TM-1_ICR D08 and TM-1_HAU_D08. f,

Heatmap of TM-1 Hi-C contact data alignment against TM-1_HAU D08. The signal in the diagonal region was discontinuous, suggesting that the sequences of TM-1_HAU D08 were not properly anchored and oriented. The regions marked with triangles formed of dashed lines are misassembled regions (incorrectly oriented). g, Heatmap of TM-1 Hi-C alignment against TM-1_ICR D08. The signal in the diagonal region is continuous, indicating that our sequence is properly assembled.

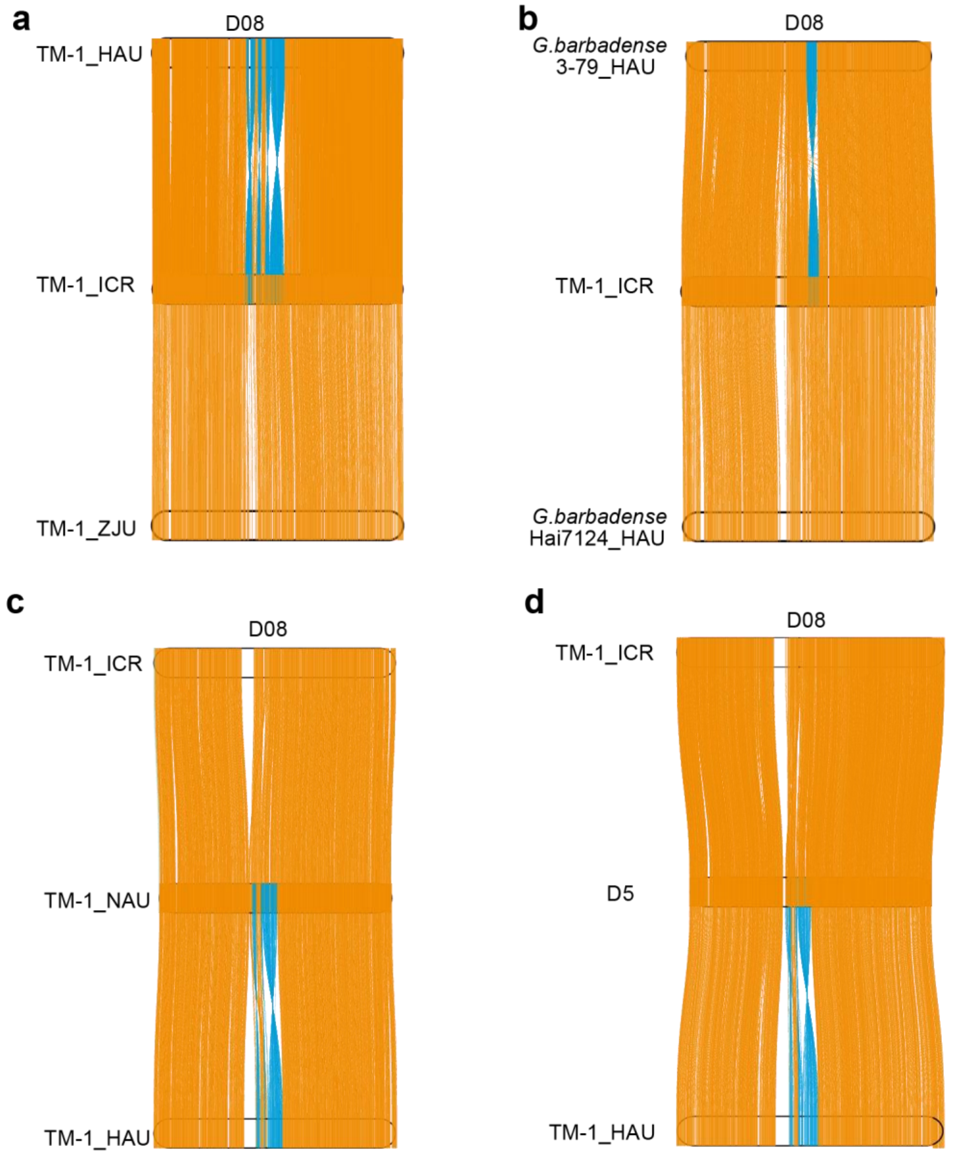

Supplementary Figure 6. Genomic comparison between TM-1_ICR D08 with D08 from previously published cotton species and upland cotton culitvars (collinearity as assessed using the nucumer function in Mummer). a, Genomic comparison of TM-1_ICR D08 with the TM-1_HAU D08 and with TM-1_ZJU. b, Genomic comparison of TM-1_ICR D08 with the D08 of *G. barbadense* acc. 3-79 (HAU) and Hai7124. c, Genomic comparison of TM-1_NAU D08 with the D08 of TM-1_ICR and TM-1_HAU. d, Genomic comparison of D5 Chr04 (D08) with the D08 of TM-1_ICR and TM-1_HAU. The blue line indicates the inverted regions between D08 chromosomes from two different assemblies. Those regions which are not covered by colored lines in (c) and (d) indicate the new assemblies in the novel genomes produced by PacBio Sequencing.

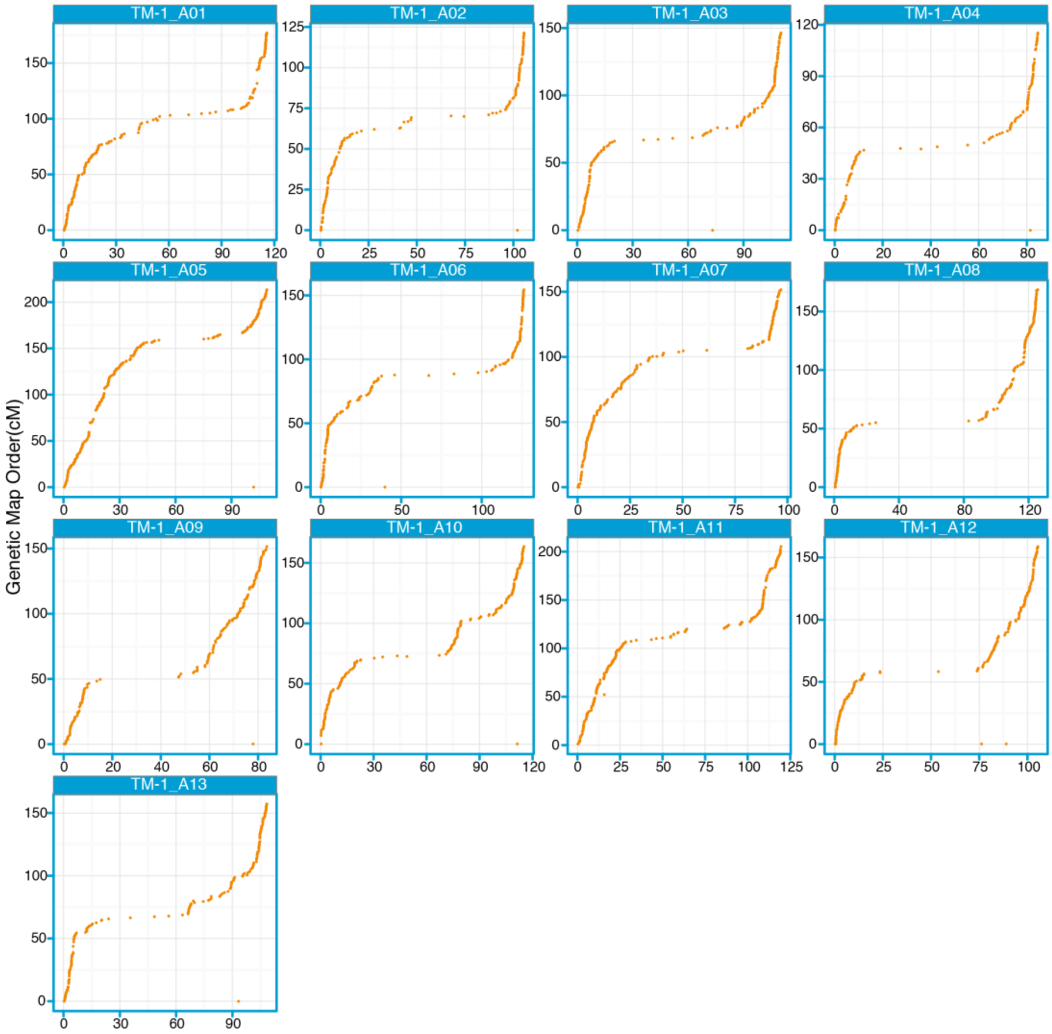

Supplementary Figure 7. Evaluation of Hi-C directed chromosome assembly with a genetic map for the A_t subgenome in TM-1. The assembled chromosomes were compared with the previously published[2] genetic map between *G. hirsutum* and *G. barbadense*. The x-axes represent the physical positions of sequences in the assembled chromosomes (Mb) and the y-axes represent the positions of sequences on the genetic map (cM).

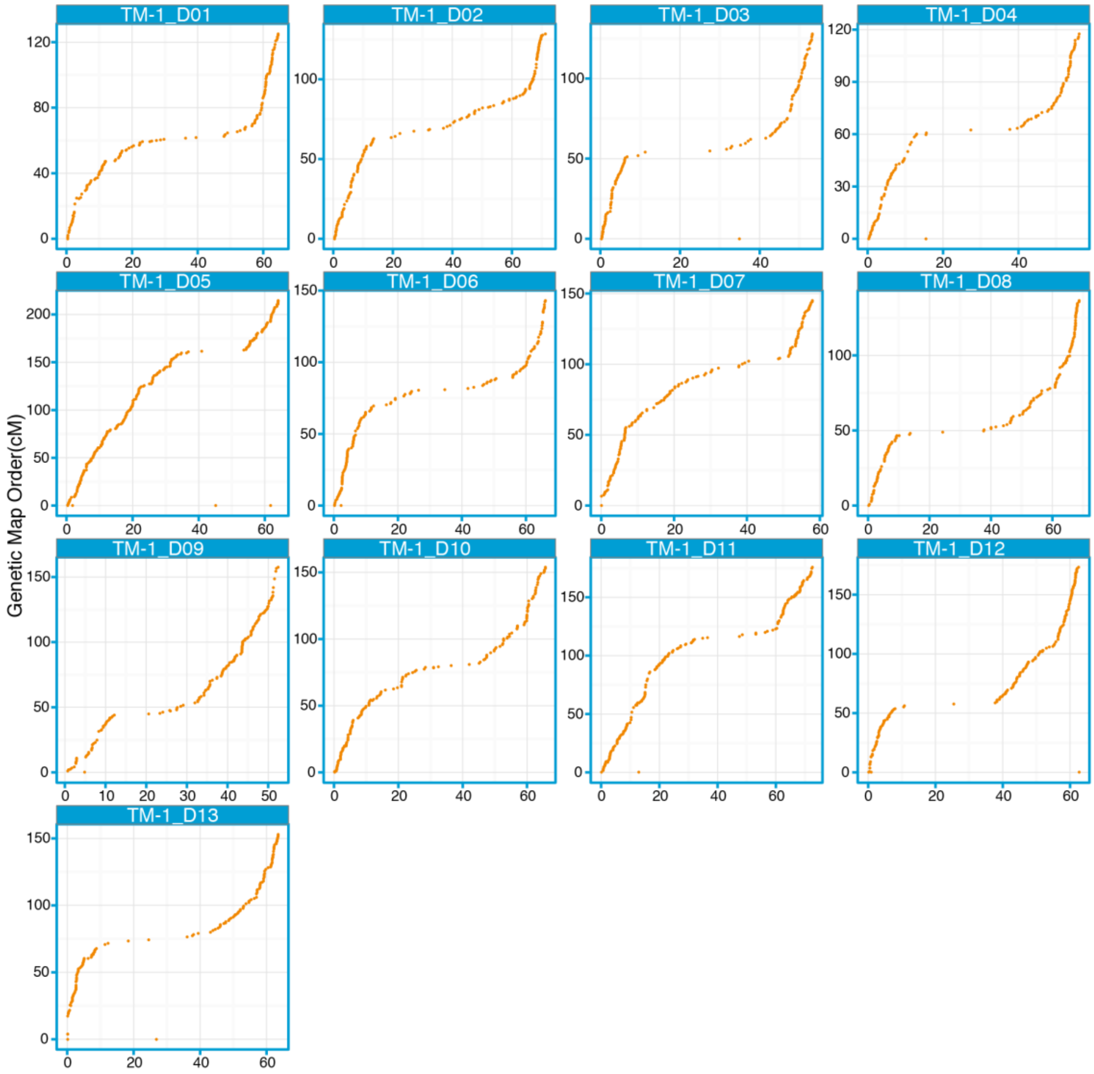

Supplementary Figure 8. Evaluation of Hi-C directed chromosome assembly with genetic map for the D_t subgenome in TM-1. The assembled chromosomes were compared with the previously published[2] genetic map between *G. hirsutum* and *G. barbadense*. The x-axes represent the physical positions of sequences in assembled chromosomes (Mb) and the y-axes represent the positions of sequences on the genetic map (cM).

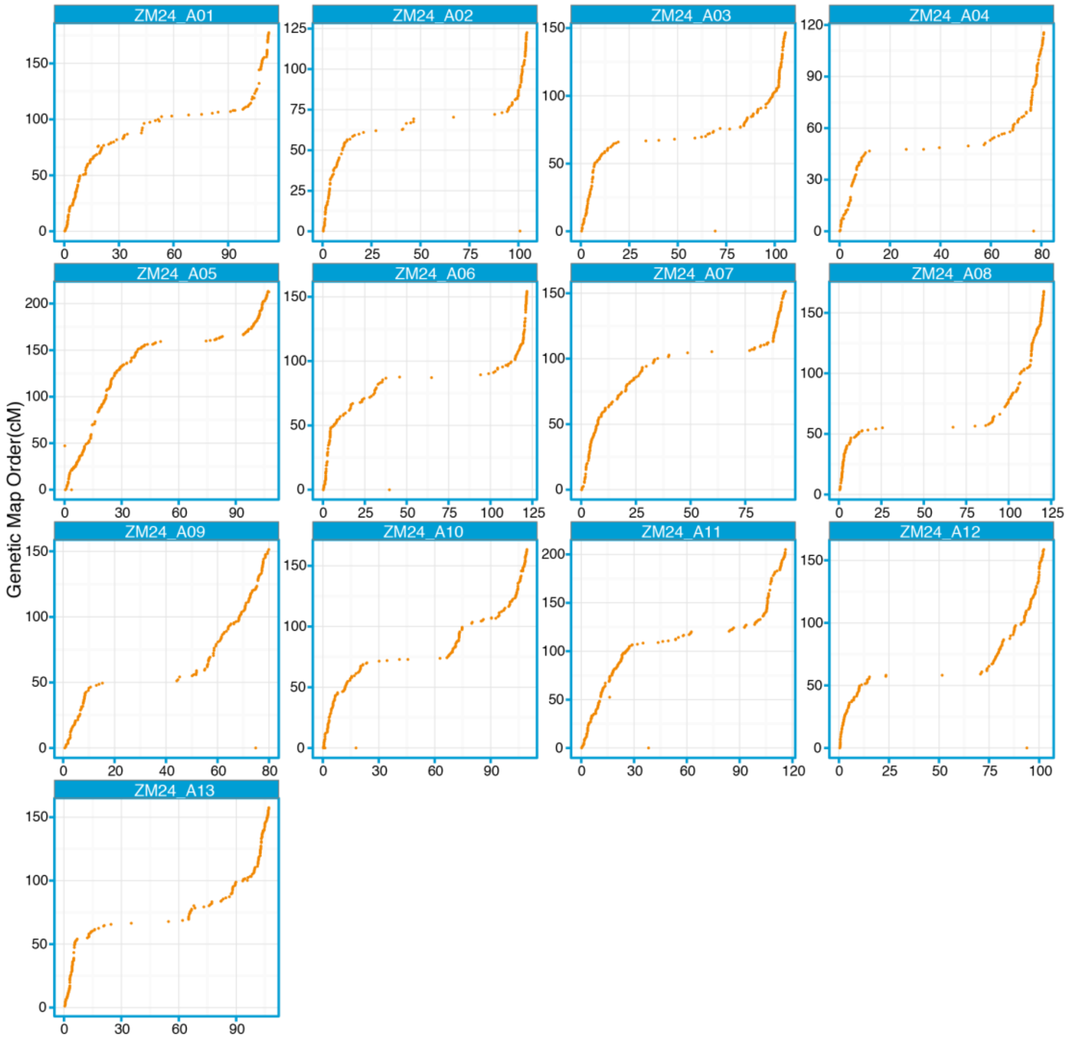

Supplementary Figure 9. Evaluation of Hi-C directed chromosome assembly with genetic map for the A_t subgenome in ZM24. The assembled chromosomes were compared with the previously published[2] genetic map between *G. hirsutum* and *G. barbadense*. The x-axes represent physical position of sequences in assembled chromosomes (Mb) and the y-axes represent the position of sequences on the genetic map (cM).

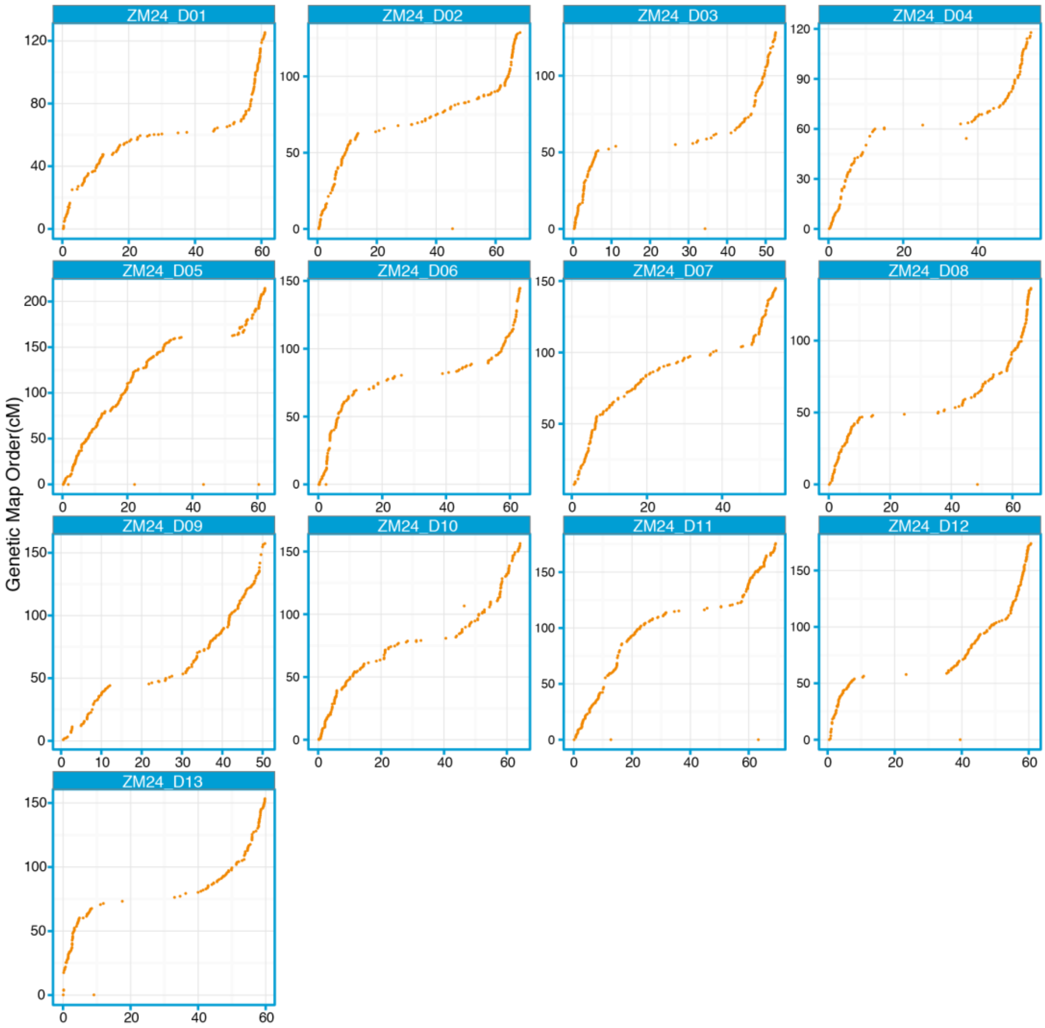

Supplementary Figure 10. Evaluation of Hi-C directed chromosome assembly with the genetic map for the D_t subgenome in ZM24. The assembled chromosomes were compared with the previously published[2] genetic map between *G. hirsutum* and *G. barbadense*. The x-axes represent the physical positions of the sequences in assembled chromosomes (Mb) and the y-axes represent the positions of the sequences on the genetic map (cM).

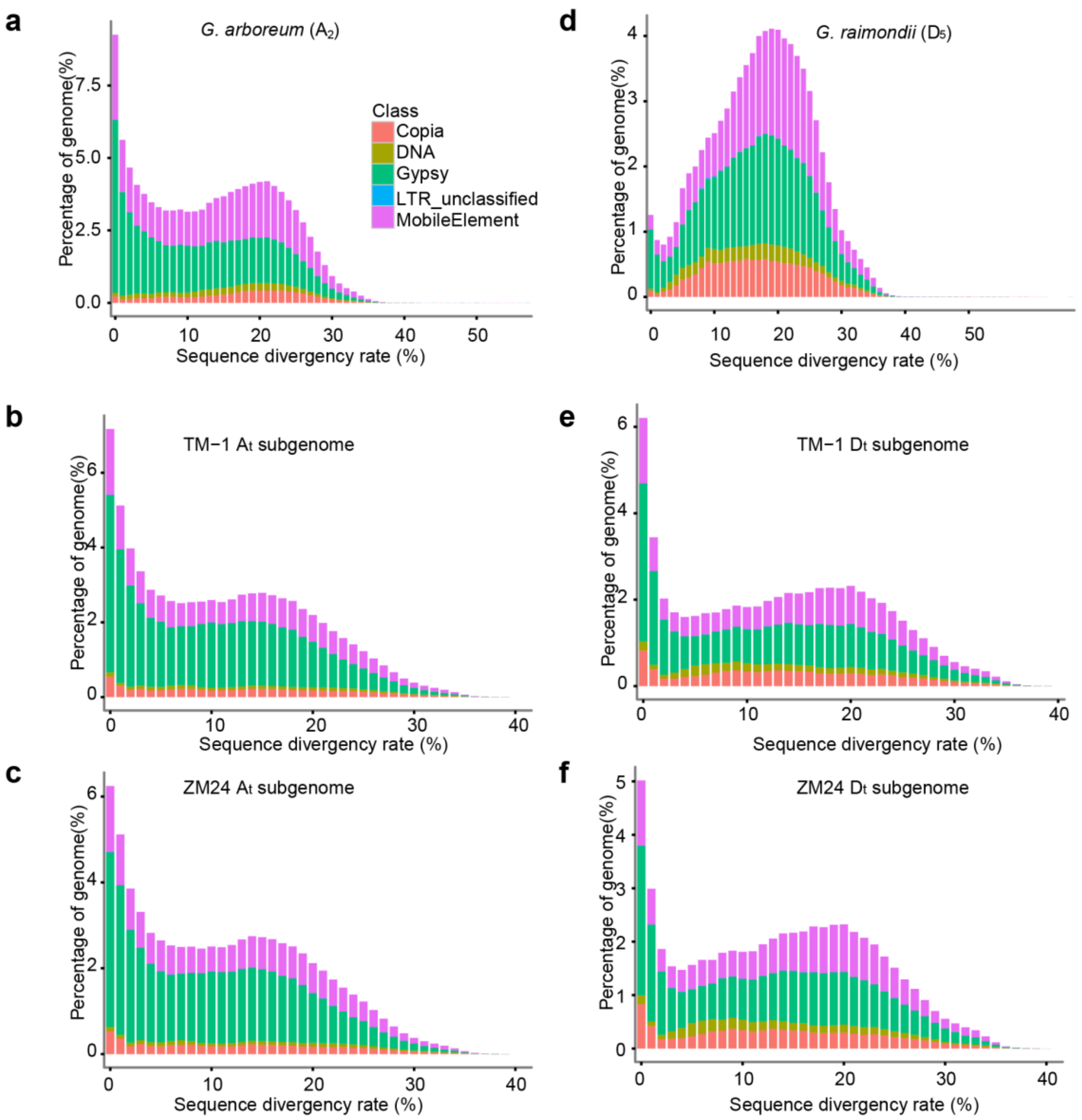

Supplementary Figure 11. The distribution of repetitive elements in *G. arboreum* (a), TM-1 A_t subgenome (b), ZM24 A_t subgenome (c), *G. raimondii* (d), TM-1 D_t subgenome (e), and ZM24 D_t subgenome (f). The x-axis represents the sequence divergence rate of repetitive elements and the y-axis represents the proportion of different types TEs in the genomes. Source data are provided in Source Data file 1.

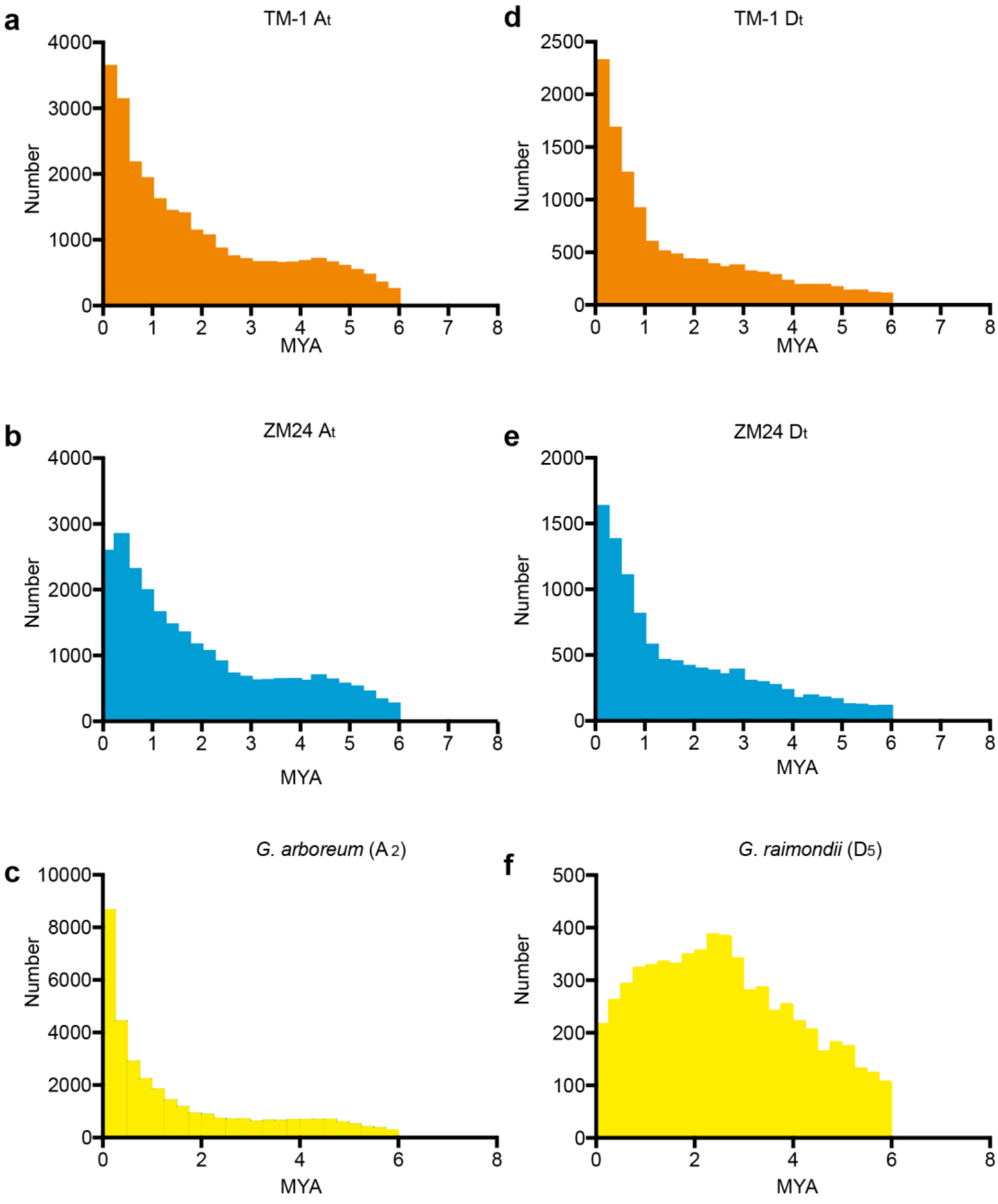

Supplementary Figure 12. The distribution of insertion ages of high-confidence full-length LTR retrotransposons in TM-1 (a, d), ZM24 (b, e), *G. arboreum* (c) and *G. raimondii* (f) genomes. Source data are provided in Source Data file 1.

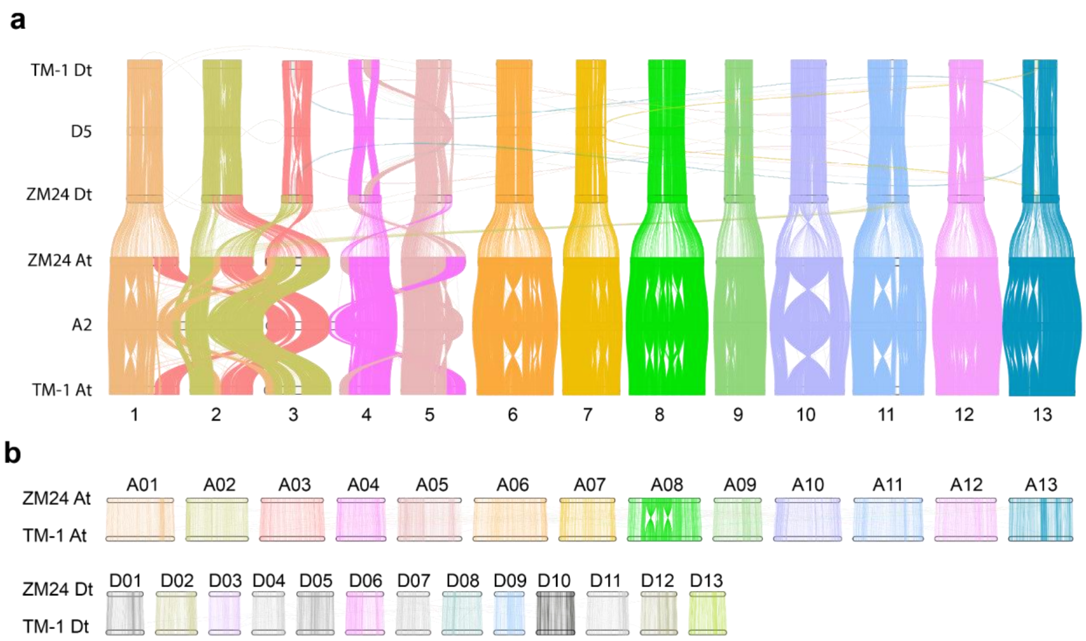

Supplementary Figure 13. Genomic comparison among the TM-1 A_t subgenome, the TM-1 D_t subgenome, the ZM24-A_t subgenome, the ZM24-D_t subgenome, D_5 (*G. raimondii*, JGI) and the A_2 genome (*G. arboreum*, ICR of CAAS) (a) and between TM-1 and ZM24 (b).

.

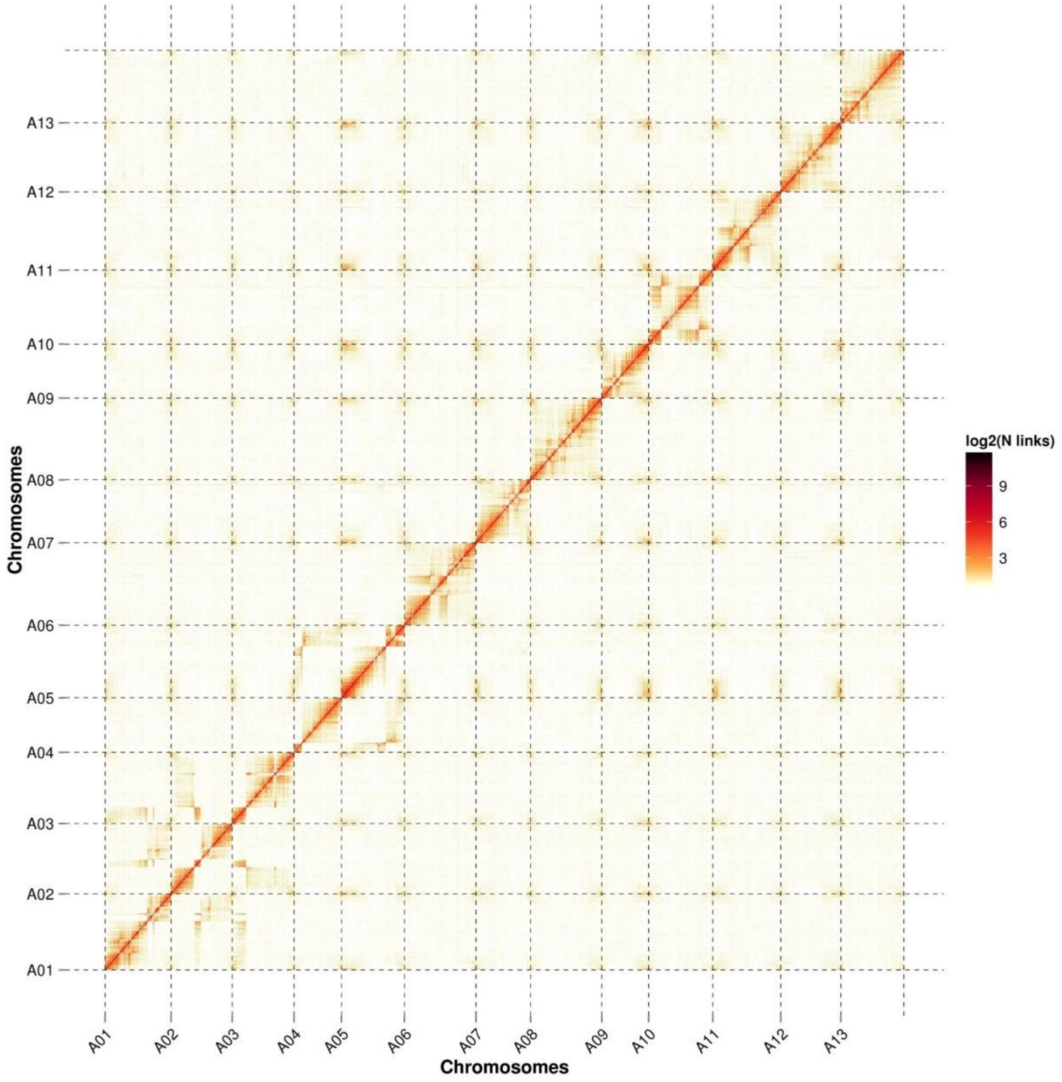

Supplementary Figure 14. The Hi-C of ZM24 contact data mapped to *G. arboreum* (A_2). The discrete interactions indicated regions likely to have chromosomal rearrangements. Strong interactions were observed between A02 and A01, between A02 and A03, and between A04 and A05.

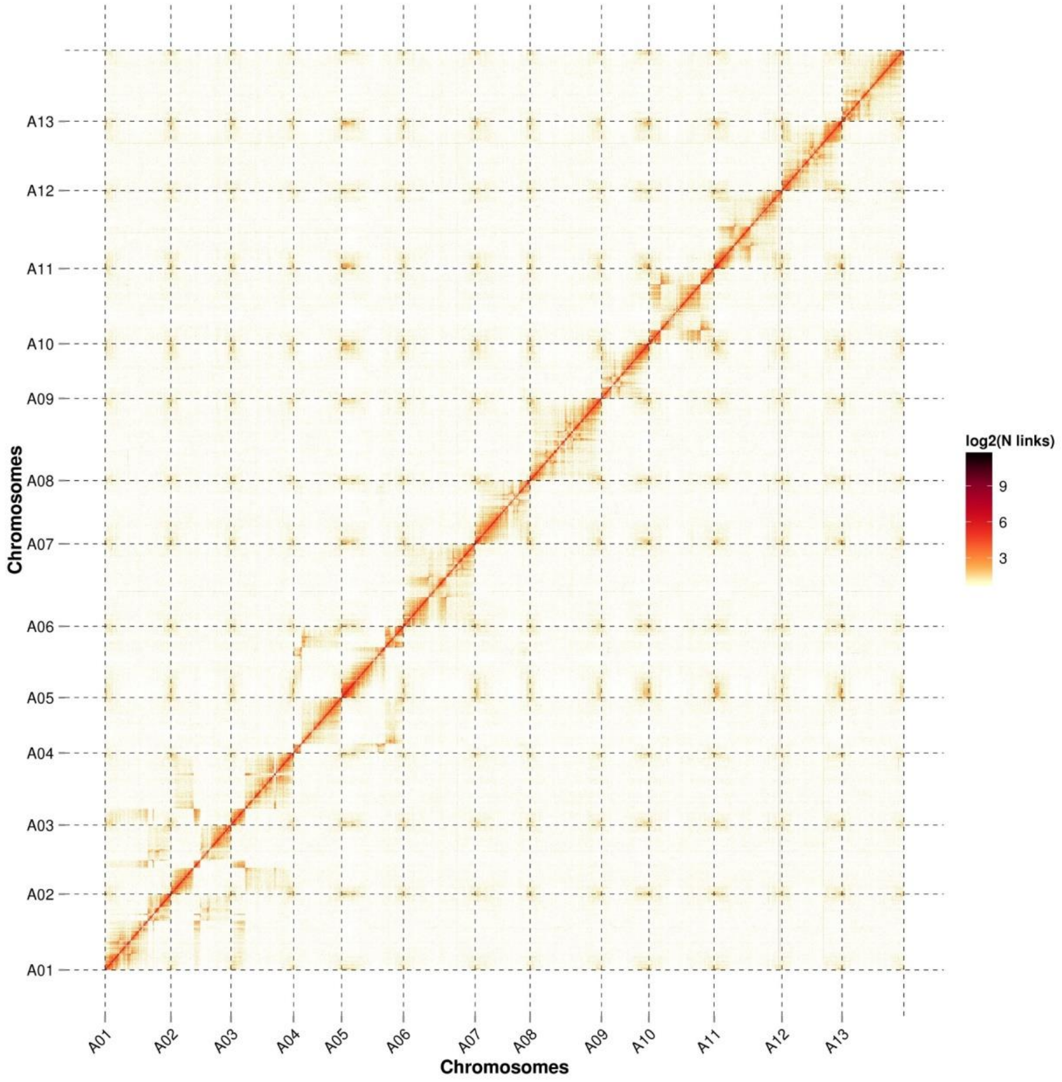

Supplementary Figure 15. The Hi-C of TM-1 contact data mapped to *G. arboreum* (A_2). The discrete interactions indicated regions likely to have chromosomal rearrangements. Strong interactions were observed between A02 and A01, between A02 and A03, and between A04 and A05.

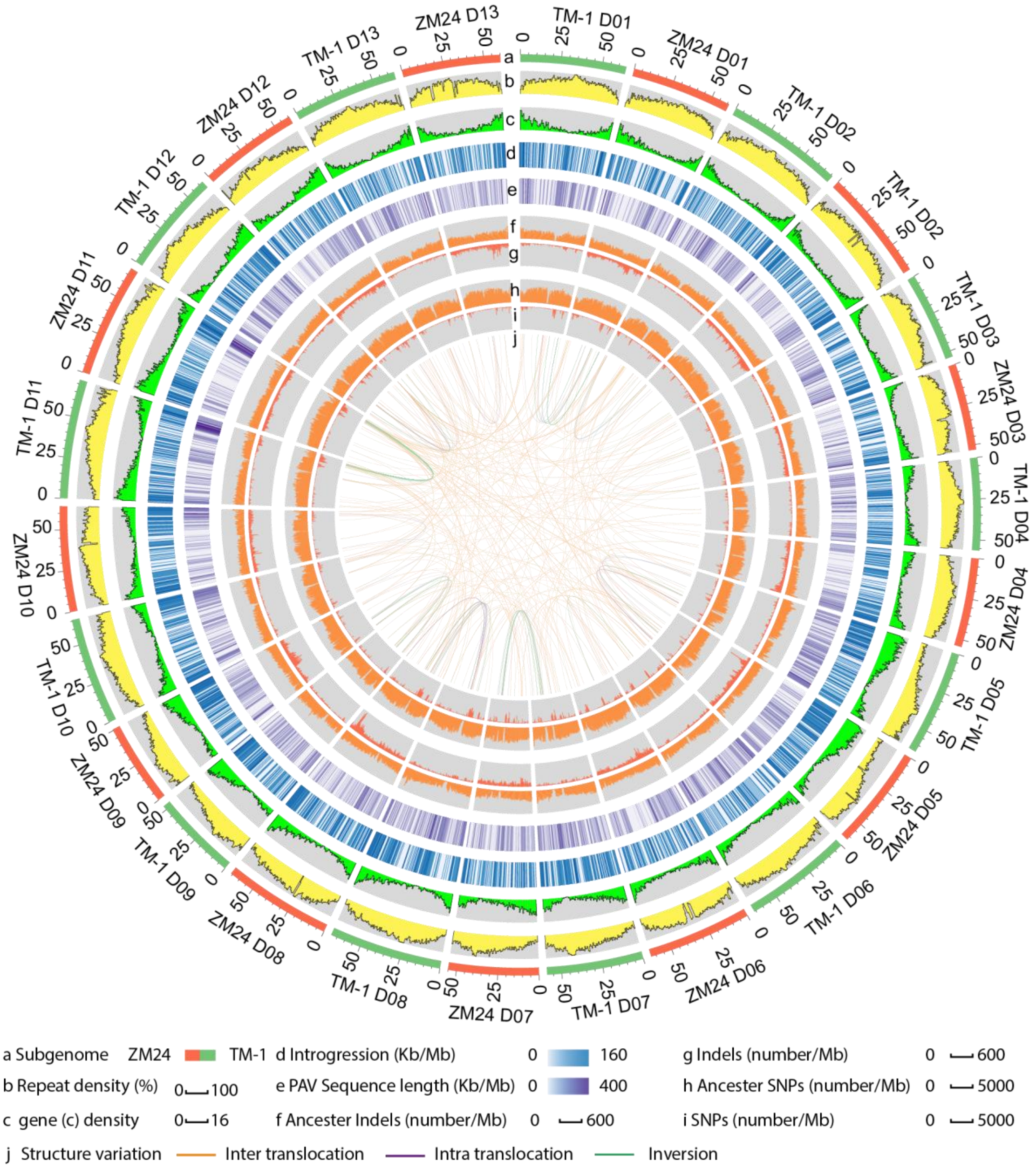

Supplementary Figure 16. Genomic landscape between TM-1 D_t subgenome and ZM24 D_t subgenome. a, Subgenome of TM-1 (green) and ZM24 (orange). b, c, Transposable elements (b) and gene density (c) in 500 Kb sliding windows. d, Introgression from chromosomes of the A_t subgenome to its counterpart chromosomes in D_t subgenome in a 500 Kb sliding windows. e, Distribution of PAV sequences in sliding windows of 500 Kb. f, g, InDels between the TM-1 and ZM24 D_t subgenomes and *G. raimondii* (D_5) (f) or between TM-1 and ZM24 D_t subgenomes (g) in 500 Kb sliding windows. h, i, SNPs between D_t subgenomes and D_5 (h) or between TM-1 and ZM24 D_t subgenomes (i) in 500 Kb sliding windows. j, large-scale variations between TM-1 and ZM24 subgenomes.

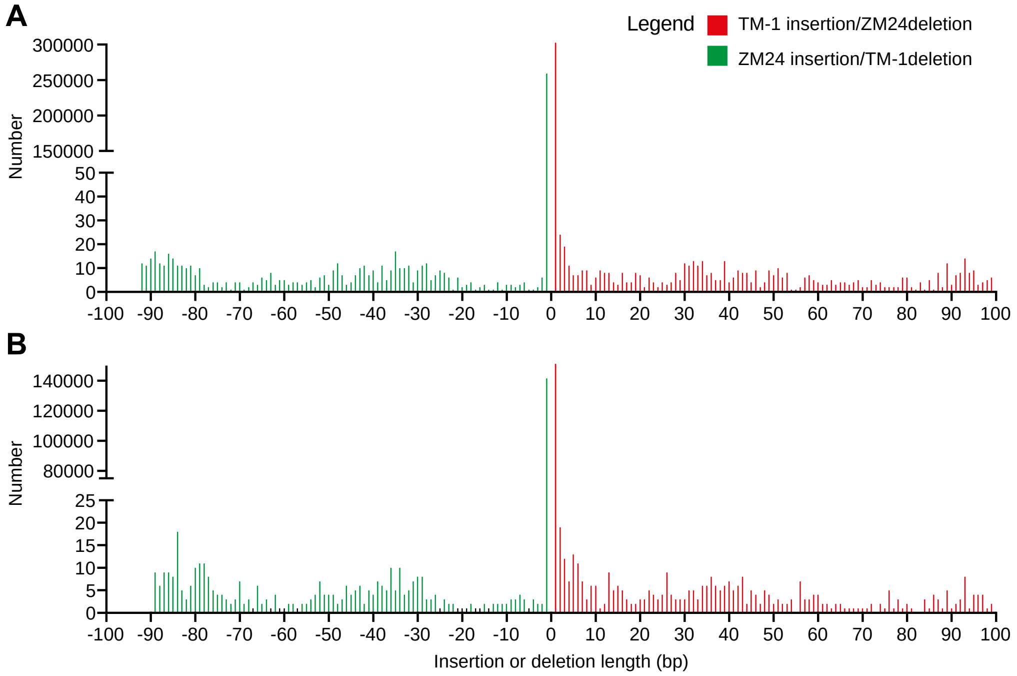

Supplementry Figure 17. Distribution of InDels at the whole-genome level (a) and on chromosome A08 (b).

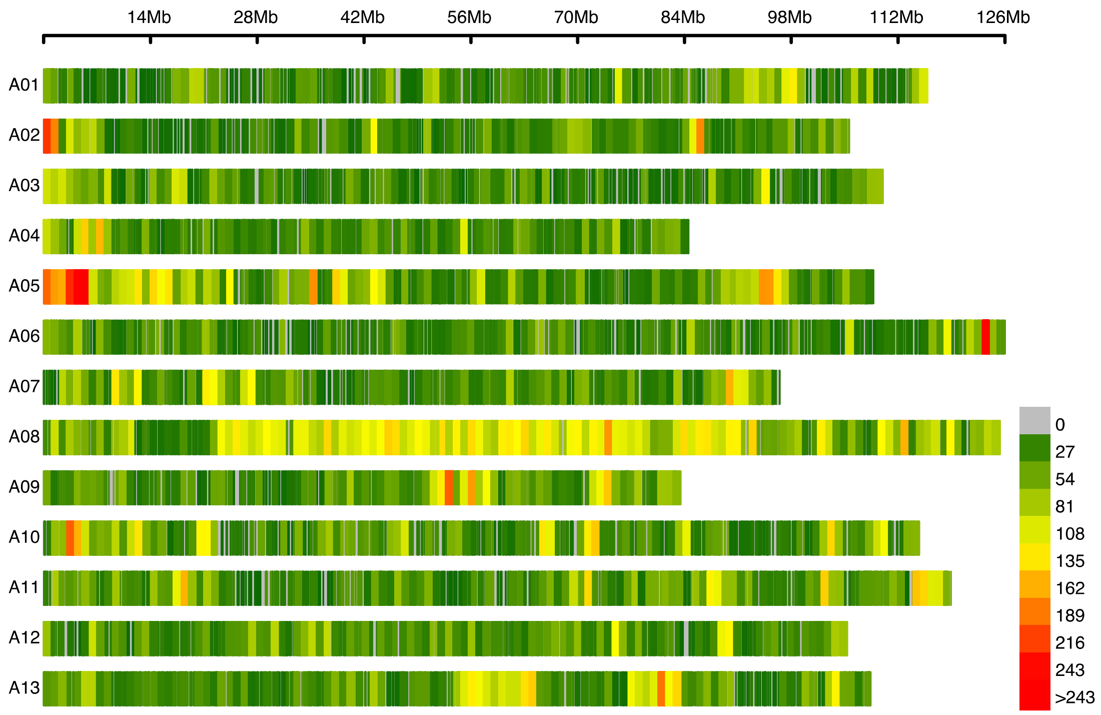

Supplementary Figure 18. InDels distribution on the A_t subgenome of TM-1 in a 1M sliding window. An InDel cluster was found on A05 (0-7 Mb) and A10 (5-6 Mb). The InDel density was relatively higher on A08 compared with the other chromosomes.

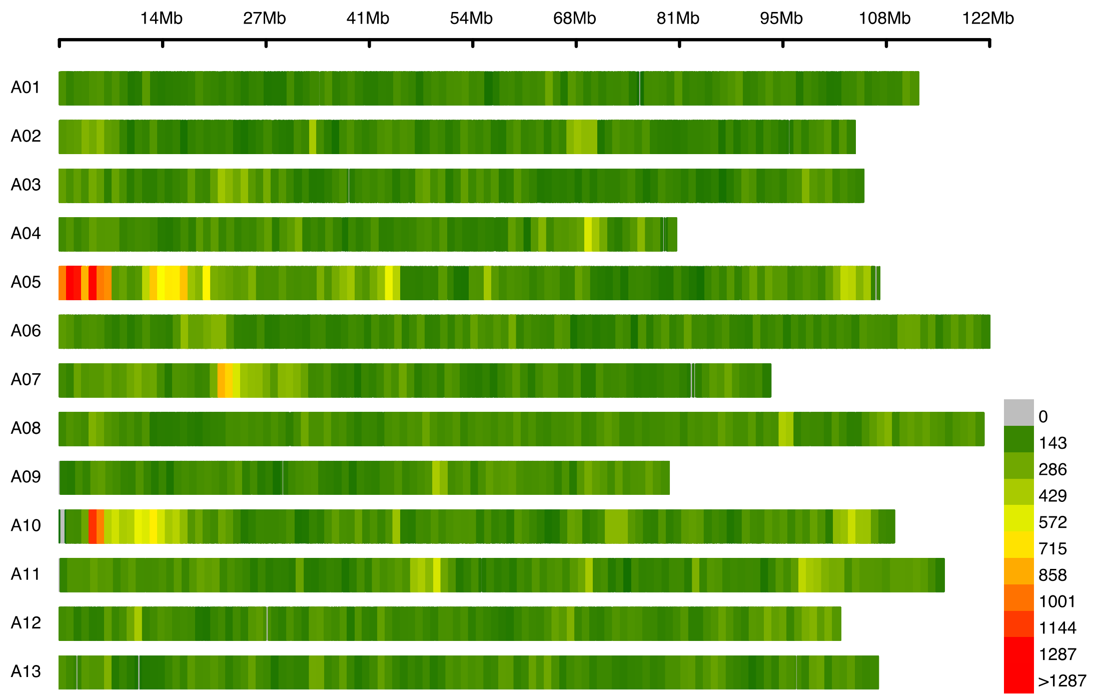

Supplementary Figure 19. InDels distribution on the A_t subgenome of ZM24 in a 1M sliding window. An InDel cluster was found on A05 (0-7 Mb) and A10 (5-6 Mb).

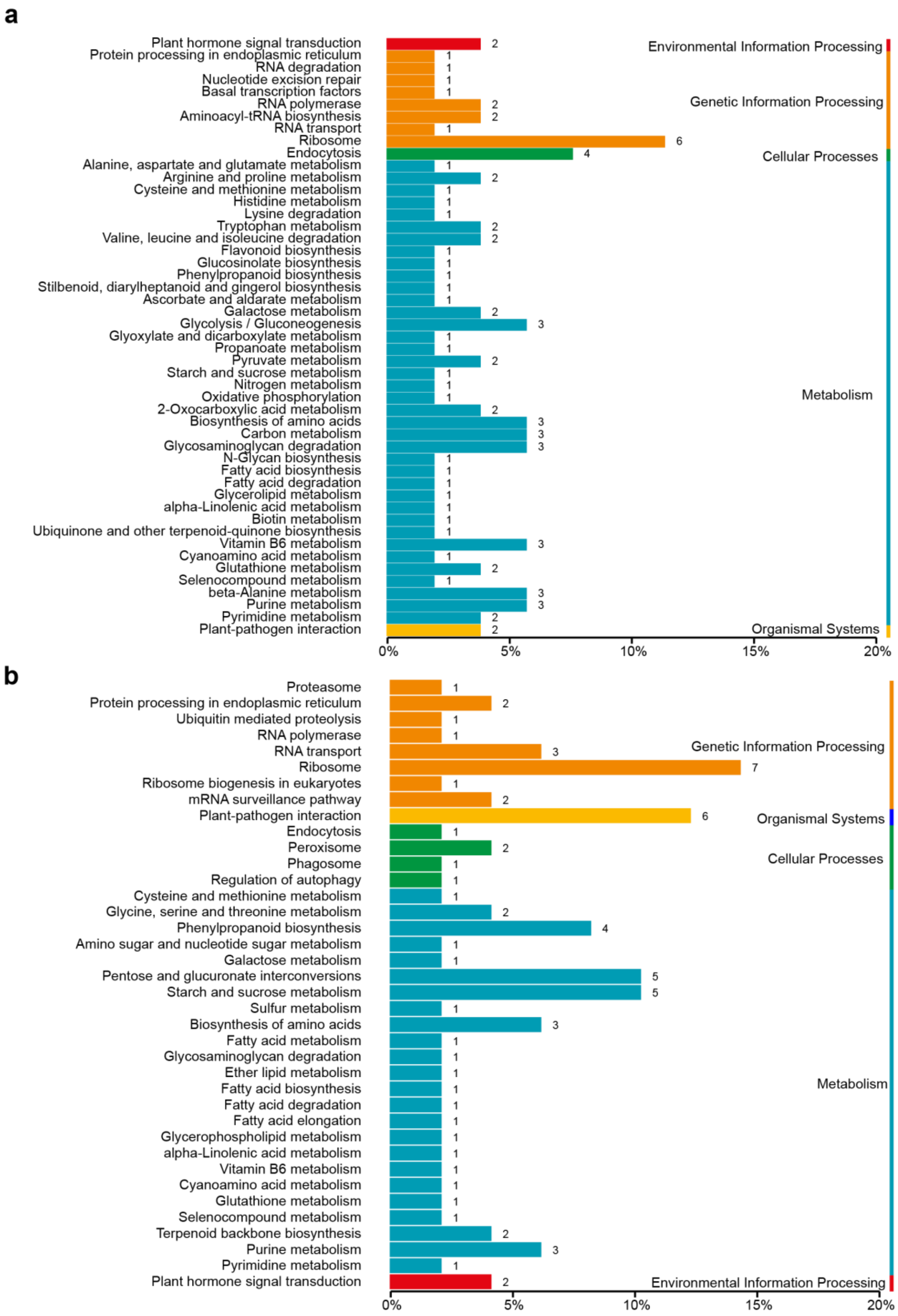

Supplementary Figure 20. KEGG annotations of the genes located in nonsyntenic regions from the TM-1 A_t subgenome (a) and the D_t subgenome (b). Source data are provided in Source Data file 1.

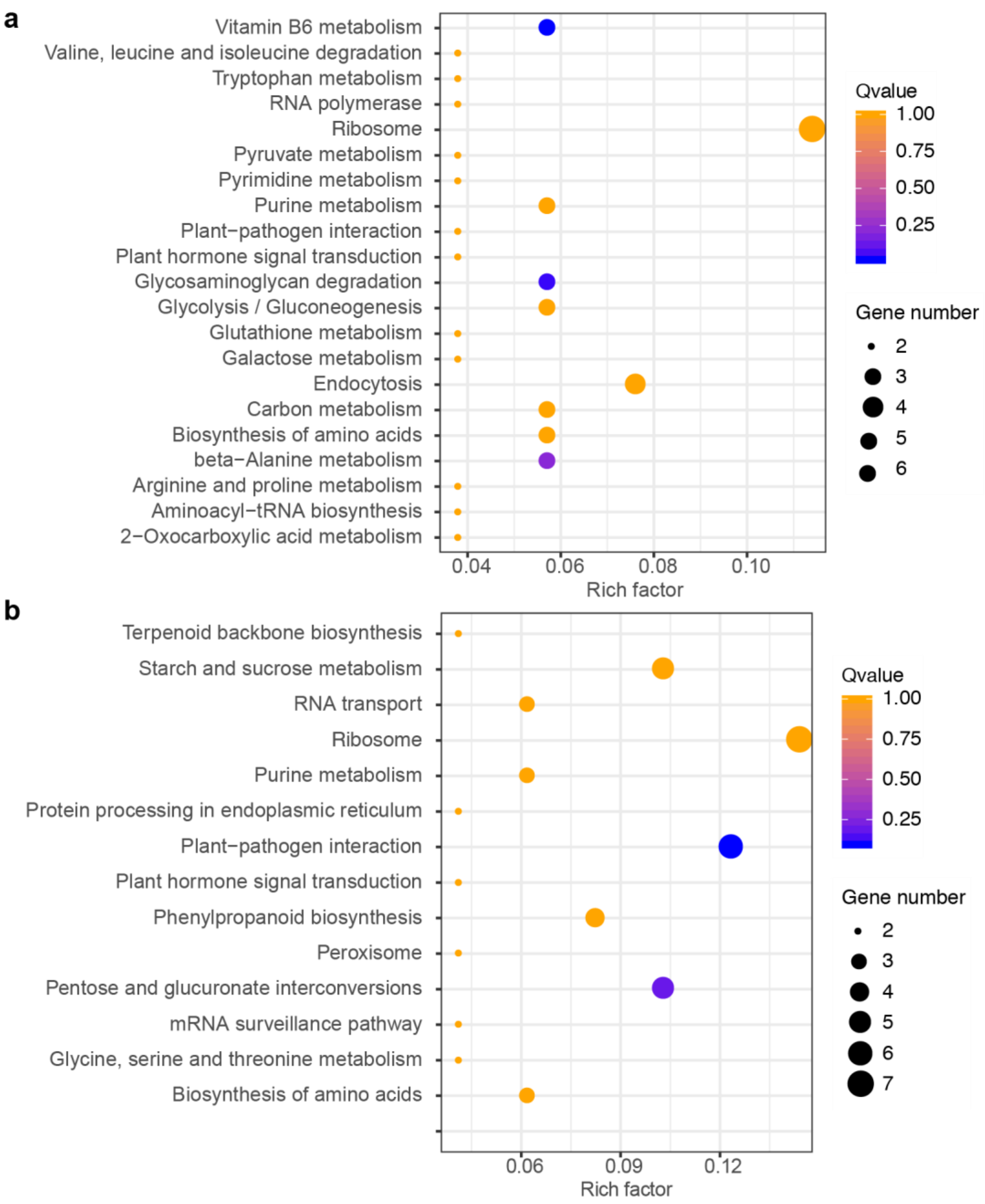

Supplementary Figure 21. KEGG enrichment of genes located in nonsyntenic regions from the TM-1 A_t subgenome (a) and the D_t subgenome (b). Source data are provided in Source Data file 1.

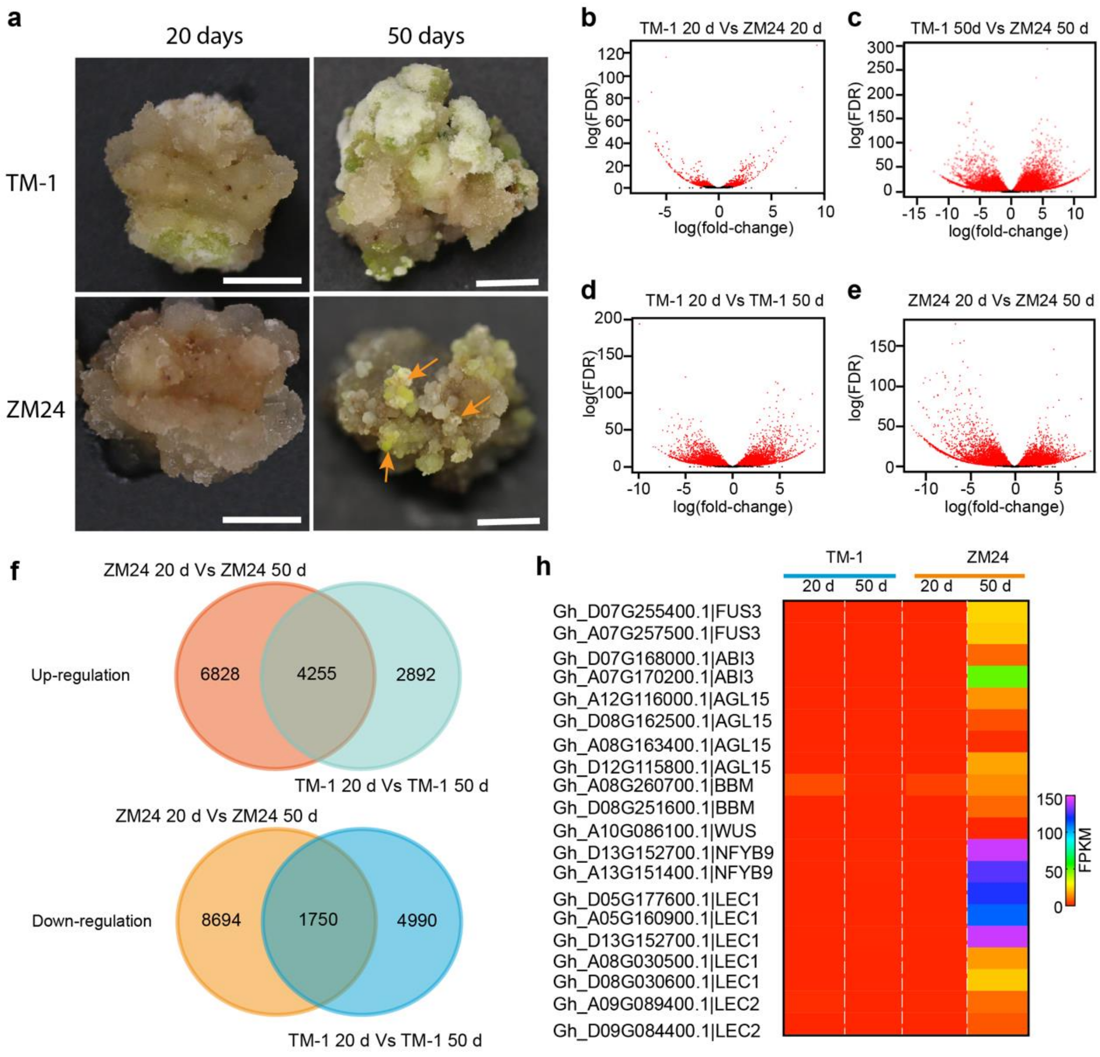

Supplementary Figure 22. mRNA-Seq sequencing and analysis of callus (Day 20 of culture) and embryogenic callus (Day 50 of culture) from TM-1 and ZM24. a，Phenotype of TM-1 and ZM24 on callus induction medium (20 days) and embryogenic callus induction medium (50 days). At 20 days, the main color of TM-1 callus of was brown, and some callus was green, whereas the ZM24 callus color was brown. At 50 days, the ZM24 callus has been transformed into embryogenic callus as orange arrows indicating in (a); however, TM-1 remained in the callus stage. The bar represents 5 mm. b-e, Volcano plot of differentially expressed genes (DEGs). The red dots were genes with a FDR less than 0.05 and an absolute value fold change larger than 1.5; such genes were defined as differentially expressed genes. f，Venn diagrams of the up-regulated DEGs and down-regulated DEGs. ZM24 20d, ZM24 50d, TM-1 20d, and TM-1 50d. h，Examples of previously reported embryogenic-callus-developoment-related genes that were among the DEGs detected in our TM-1 vs. ZM24 comparison of Day 50 calli. Source data are provided in Source Data file 1.

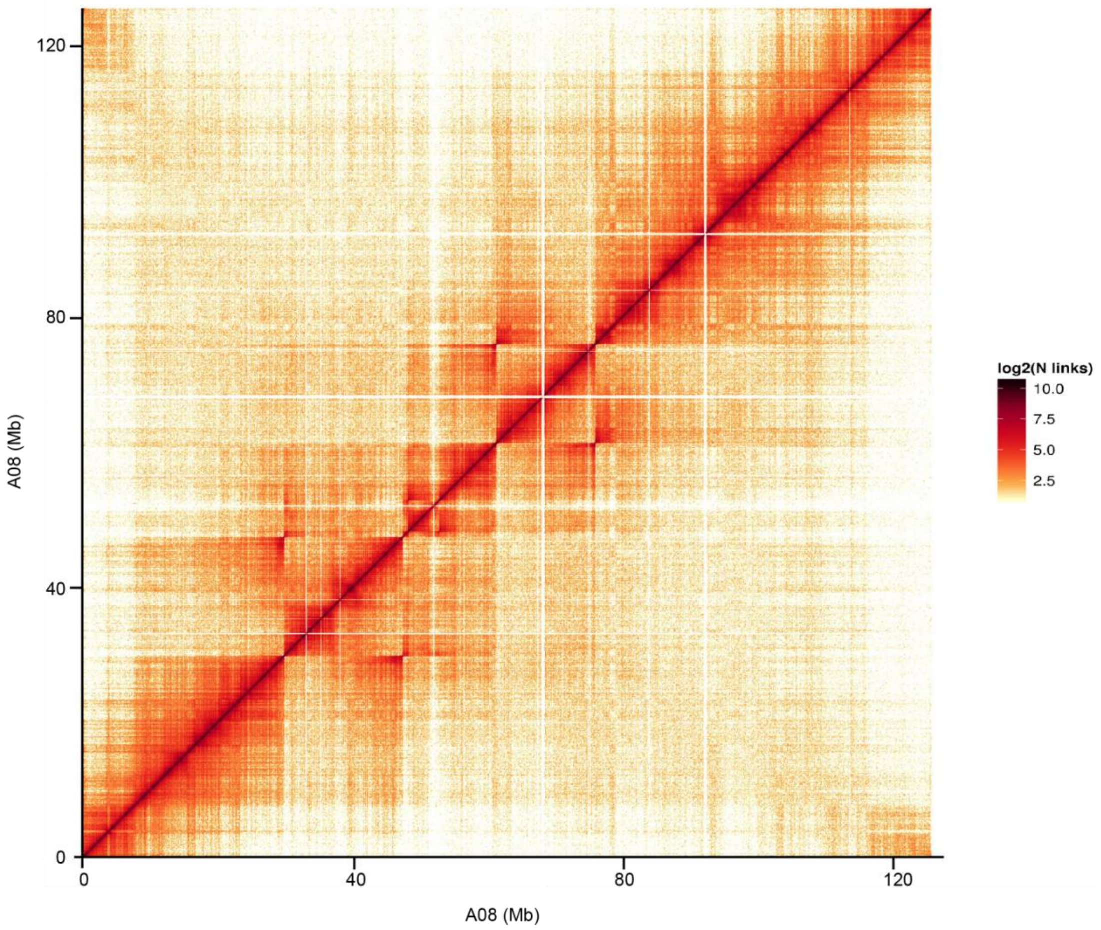

Supplementary Figure 23. The TM-1 Hi-C contact mapping against ZM24 A08 chromosome, which supports inversions between TM-1 and ZM24.

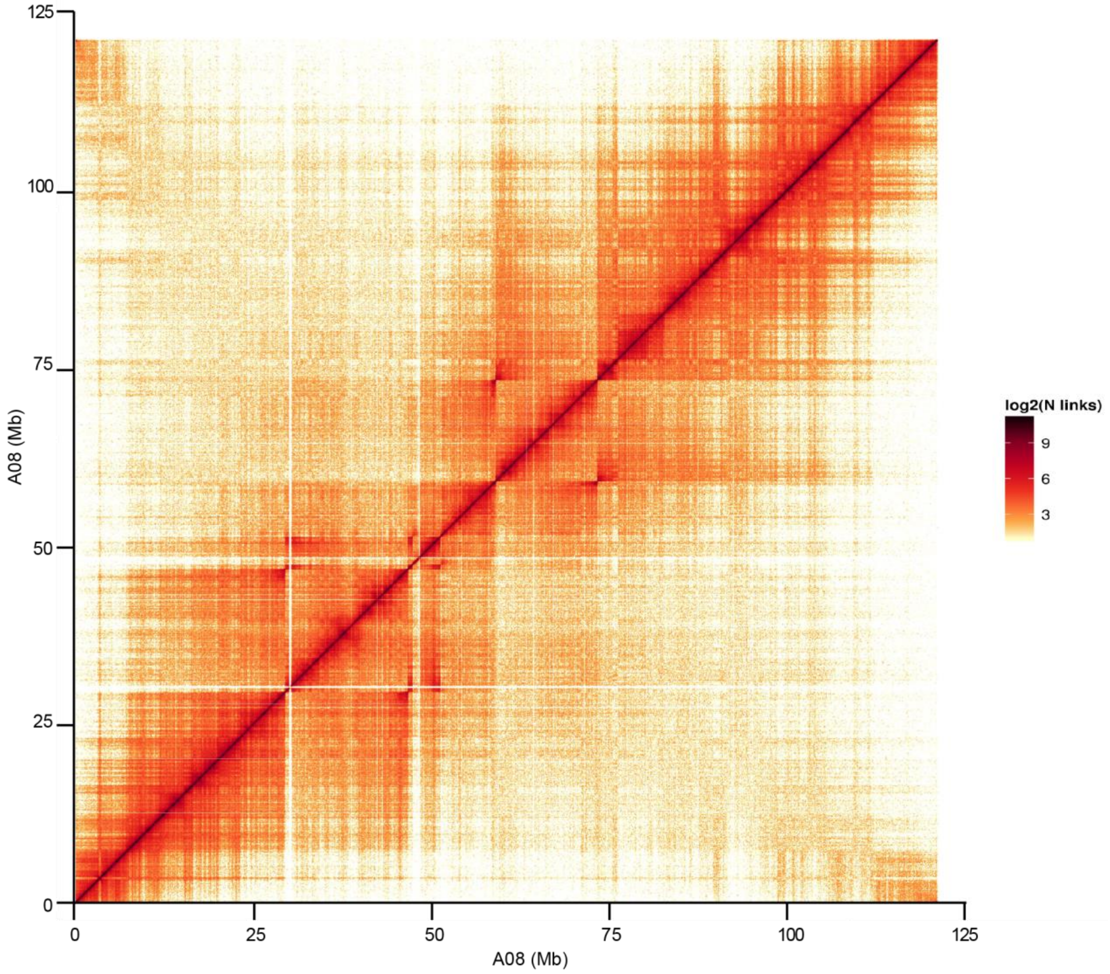

Supplementary Figure 24. The ZM24 Hi-C contact mapping against TM-1 A08 chromosome, which supports inversions between ZM24 and TM-1.

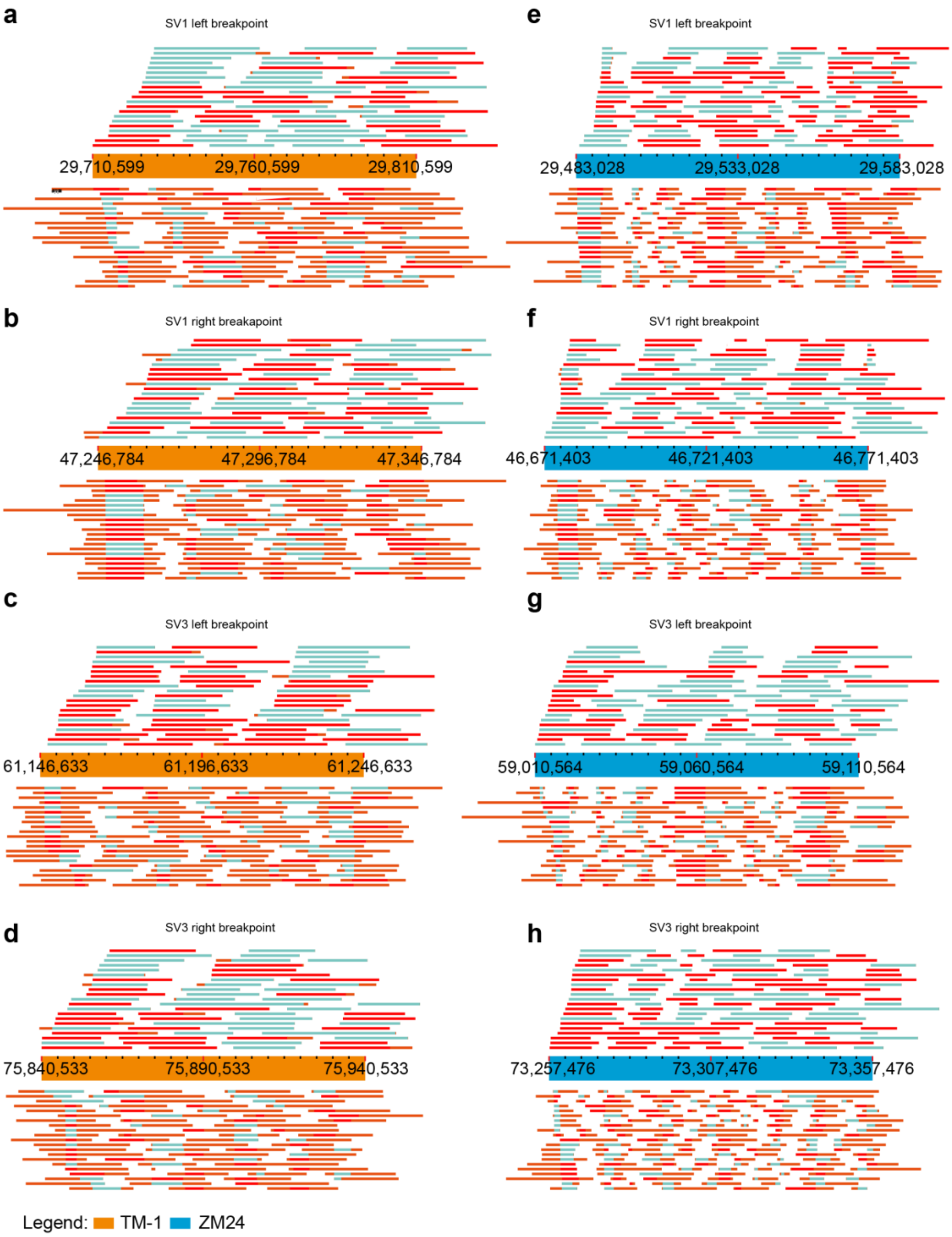

Supplementary Figure 25. PacBio long reads were mapped to the TM-1 and ZM24 genomes around the left and right breakpoints of SV1 and SV3. The 50 Kb upstream and downstream sequences of each breakpoint are displayed. The upper part of the karyotype band were the reads perfectly mapped and that under the karyotype band were well match reads. Orange color reads represent those mapped to the forward strand of the reference; the cyan reads represent those mapped to the reverse strand of the reference.

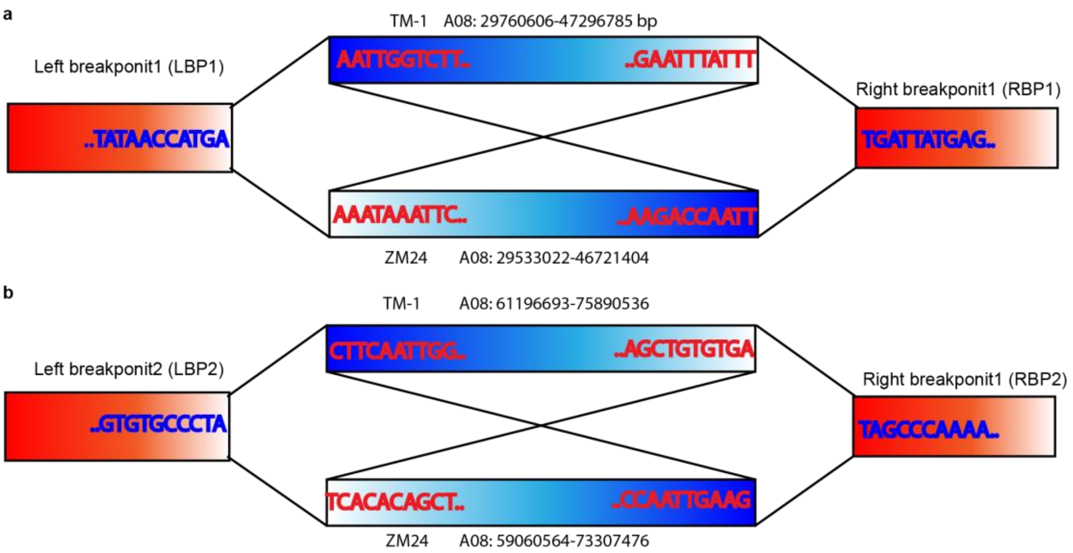

c

SV1	Left breakpoint	common fwd	GAACAGATTGATATTTTAGCTCGGG
	Left breakpoint	TM-1 rev	CCATGCAATAATACGAAATCCACAT
	Left breakpoint	ZM24 rev	CACCCAATTTGAGCAGTAGTGTTTT
	Right breakpoint	TM-1 fwd	GTTGTTTCCCCAAGTCTAGGTATTAC
	Right breakpoint	ZM24 fwd	GTTGTTTCCCCAAGTCTAGGTATTAC
	Right breakpoint	common rev	CCTGGATCAACACATTCGTATA
SV3	Left breakpoint	common fwd	GTATGATTGAATGGTGATTATCACAT
	Left breakpoint	TM-1 rev2	AAGTCGAGGTGGCGGTCTAAAA
	Left breakpoint	ZM24 rev2	CTCAATAGAATACCAATCAATTTACC
	Right breakpoint	TM-1 fwd2	GGATTATAAGTTTTTGAAGTCTCCCC
	Right breakpoint	ZM24 fwd2	TGTTGTGATTGATCGGTAATGCCTC
	Right breakpoint	common rev	TAGATAACTTTTGGAGTCAAAAGTAG

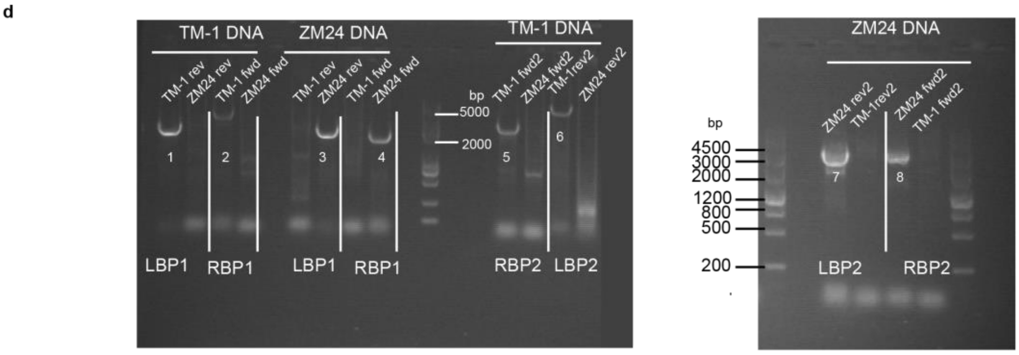

Supplementary Figure 26. PCR sequencing of the A08 inversions breakpoints in the TM-1 and ZM24 genomes. The primer sequences used to validate the breakpoints in TM-1 and ZM24 are shown in the figure. a, b, Schematic diagram for SV1 and SV3.The positions of left and right breakpoint for SV1 and SV3 in TM-1 and ZM24 are displayed near the gradient blue boxes. c, Primers were designed to span the breakpoints for more than 1,500 bp on each side. d, PCR results for SV1 and SV3. PCR products for band 1-8 were confirmed by Sanger-sequencing. Source data are provided in Source Data file 1.

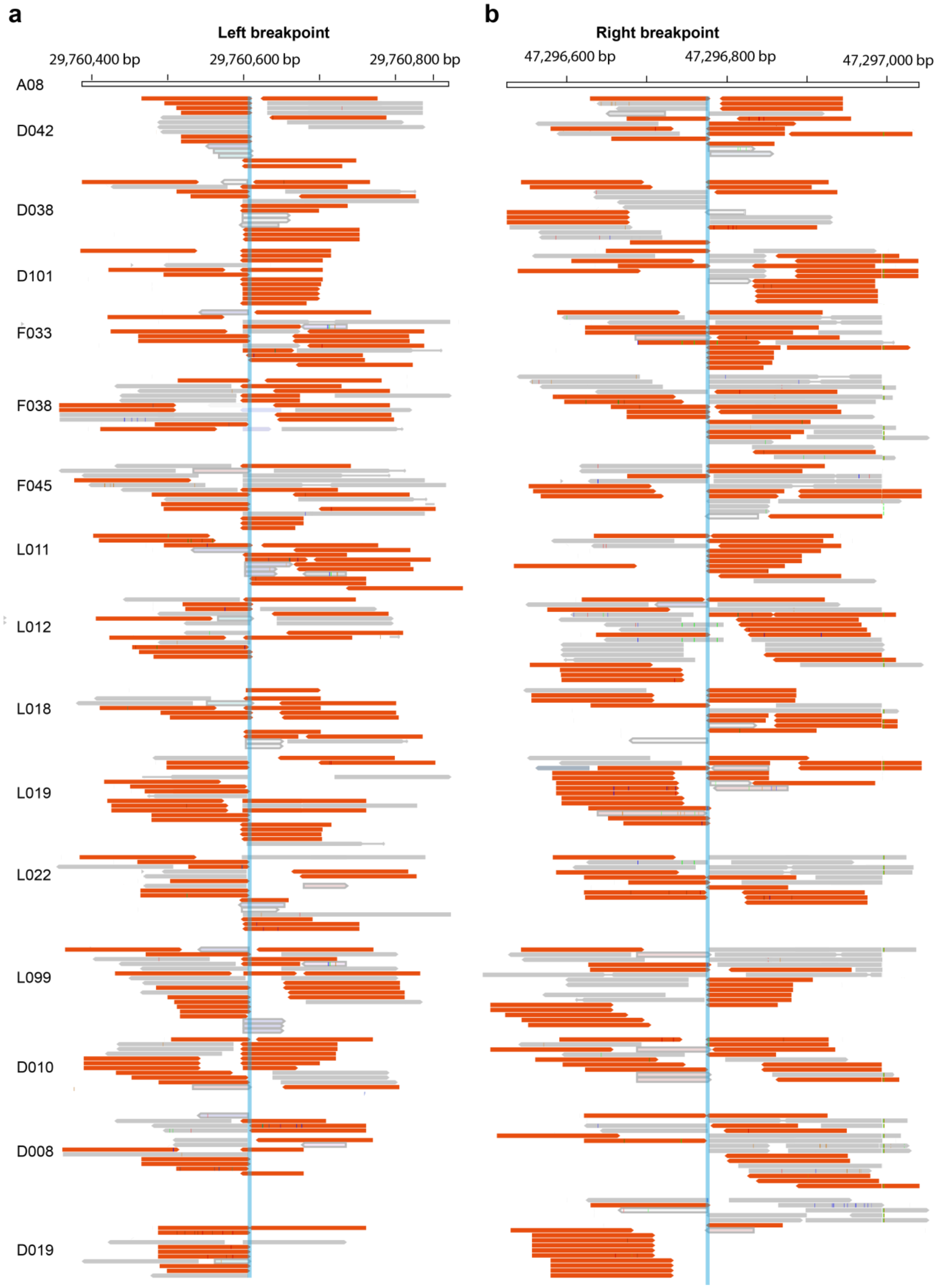

Supplementary Figure 27. Aligning the short-reads of different accessions from the ZM24-like group against the TM-1 genome using BWA software. The blue boxes indicate the left and right breakpoint of SV1. No reads or mate pair reads spanned the breakpoints in (a) or (b). The orange-red colored reads had an abnormal insert-size with their mate-pair reads. The grey color reads had normal insert-size with

their mate-pair reads. The mate-pair reads of the orange-red reads in (a) were mapped to the breakpoint in (b). Similarly, the mate-pair reads of orange-red reads in (b) were mapped to the breakpoint in (a).

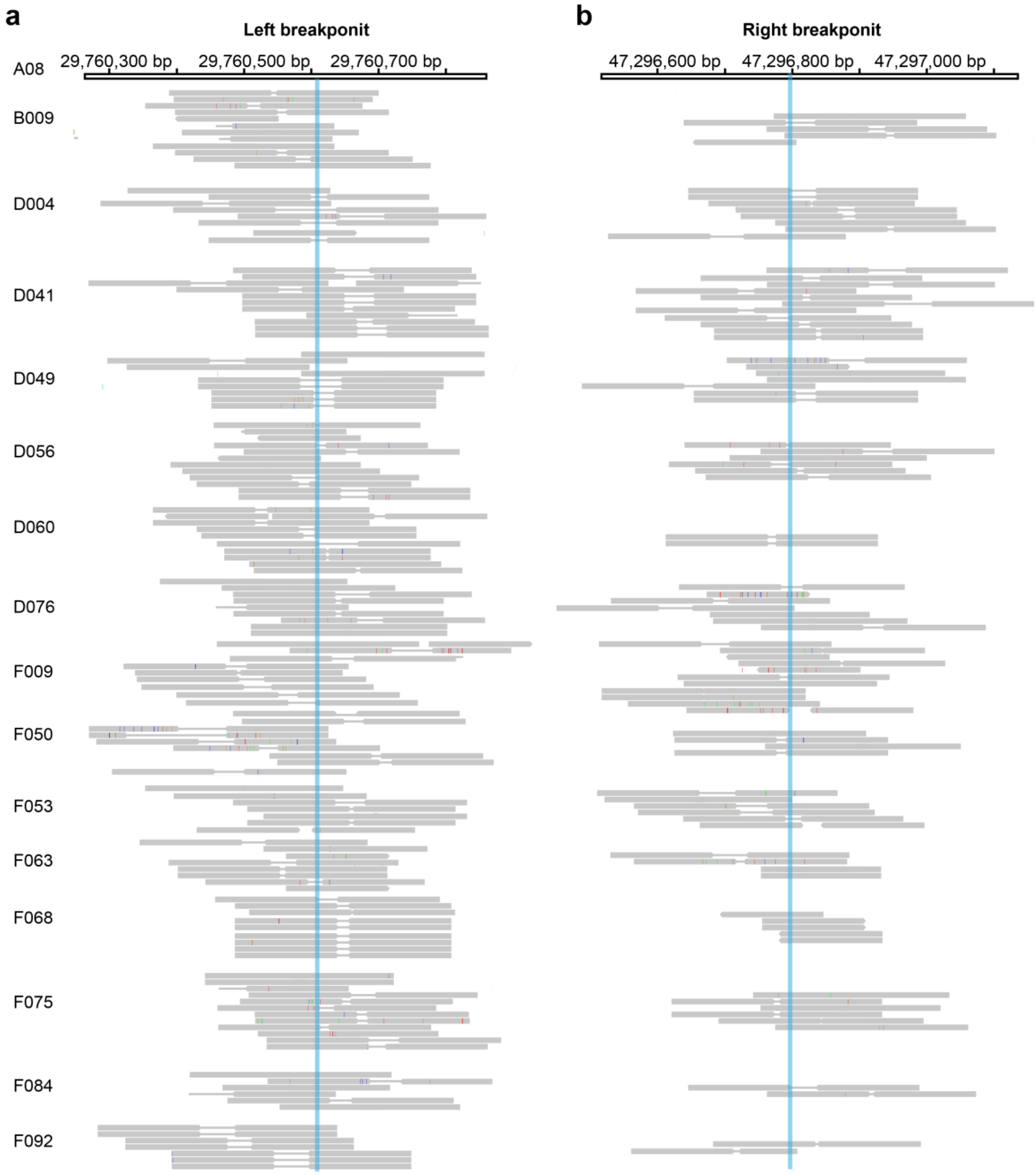

Supplementary Figure 28. Aligning the short-reads of different accessions from the TM-1-like group against the TM-1 genome using BWA software. The blue boxes indicate the left and right breakpoints of SV1. Short-reads or mate pair reads were found spanning the breakpoints in (a) and (b).

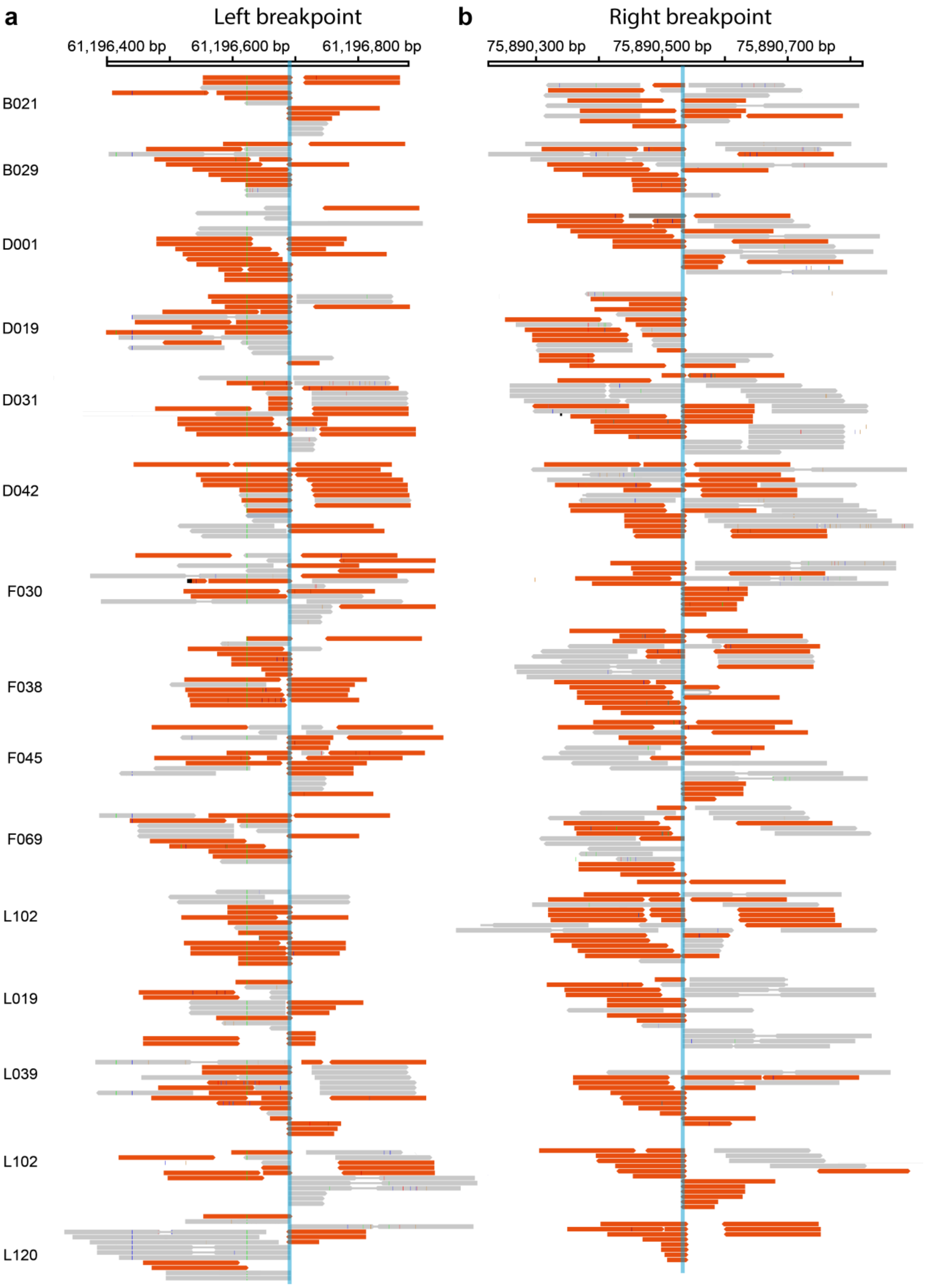

Supplementary Figure 29. Aligning the short-reads of different accessions from the ZM24-like group to the TM-1 genome using BWA software. The blue boxes indicate the left and right breakpoints of SV3. No reads or mate pair reads were spanning the breakpoints in (a) or (b).The orange-red colors reads had an abnormal insert-size with their mate-pair reads. The grey color reads had normal insert-size with their mate-pair reads. The mate-pair reads of the orange-red reads in (a) were mapped

to the breakpoint in (b). Similarly, the mate-pair reads of orange-red reads in (b) were mapped to the breakpoint in (a).

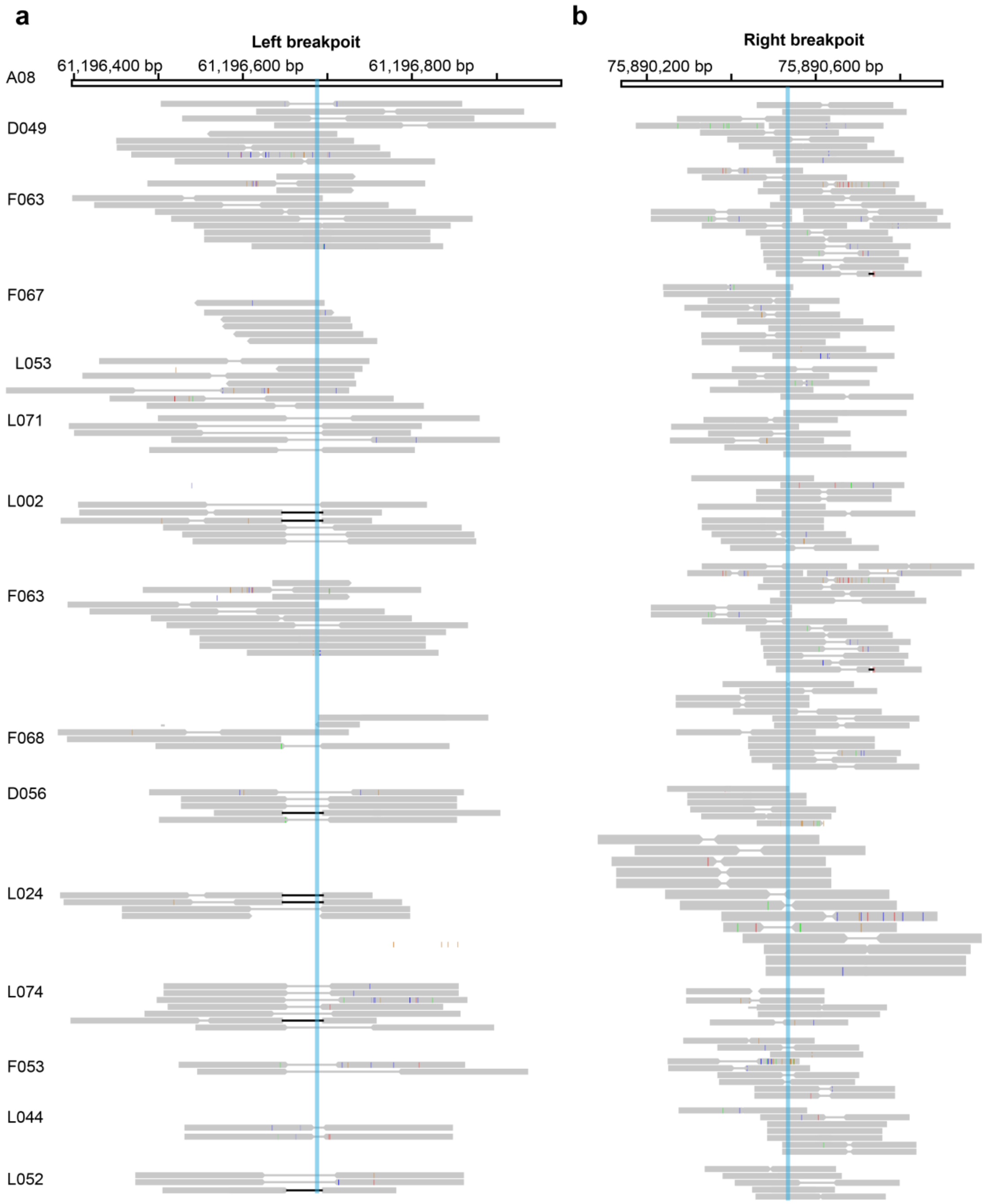

Supplementary Figure 30. Aligning the short-reads of different accessions from the TM-1-like group to the TM-1 genome using BWA software. The blue boxes indicate the left and right breakpoints of SV3. Short reads or mate read pairs were found spanning the breakpoints in (a) and (b).

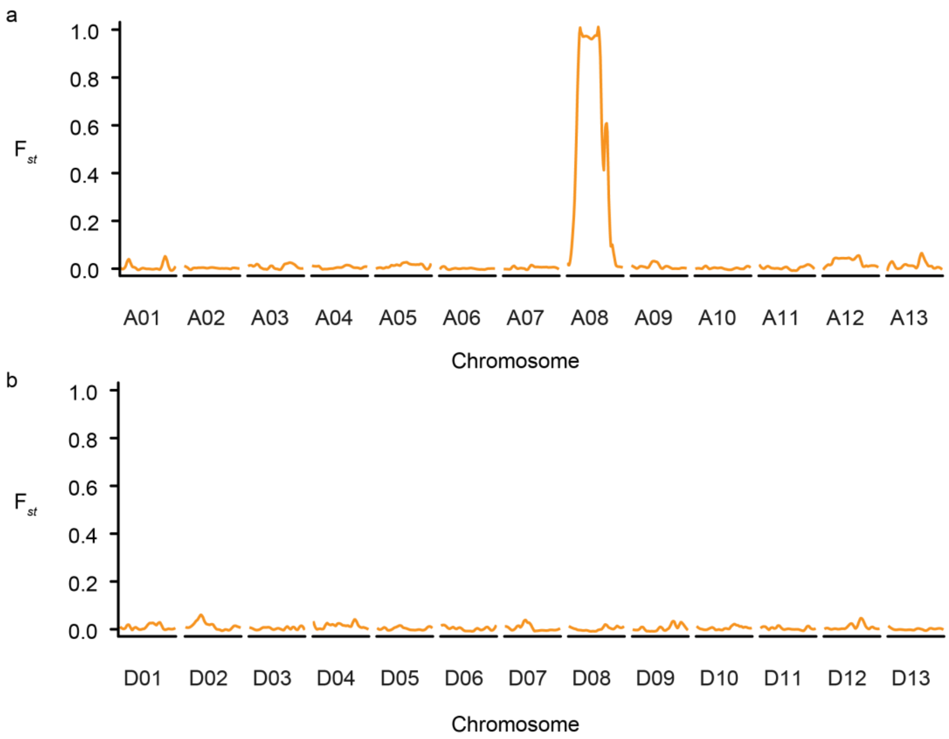

Supplementary Figure 31. Genome-wide population differentiation (F_{ST}) values between the two groups of accessions with either a TM-1-like or a ZM24-like genotype. Source data are provided in Source Data file 1.

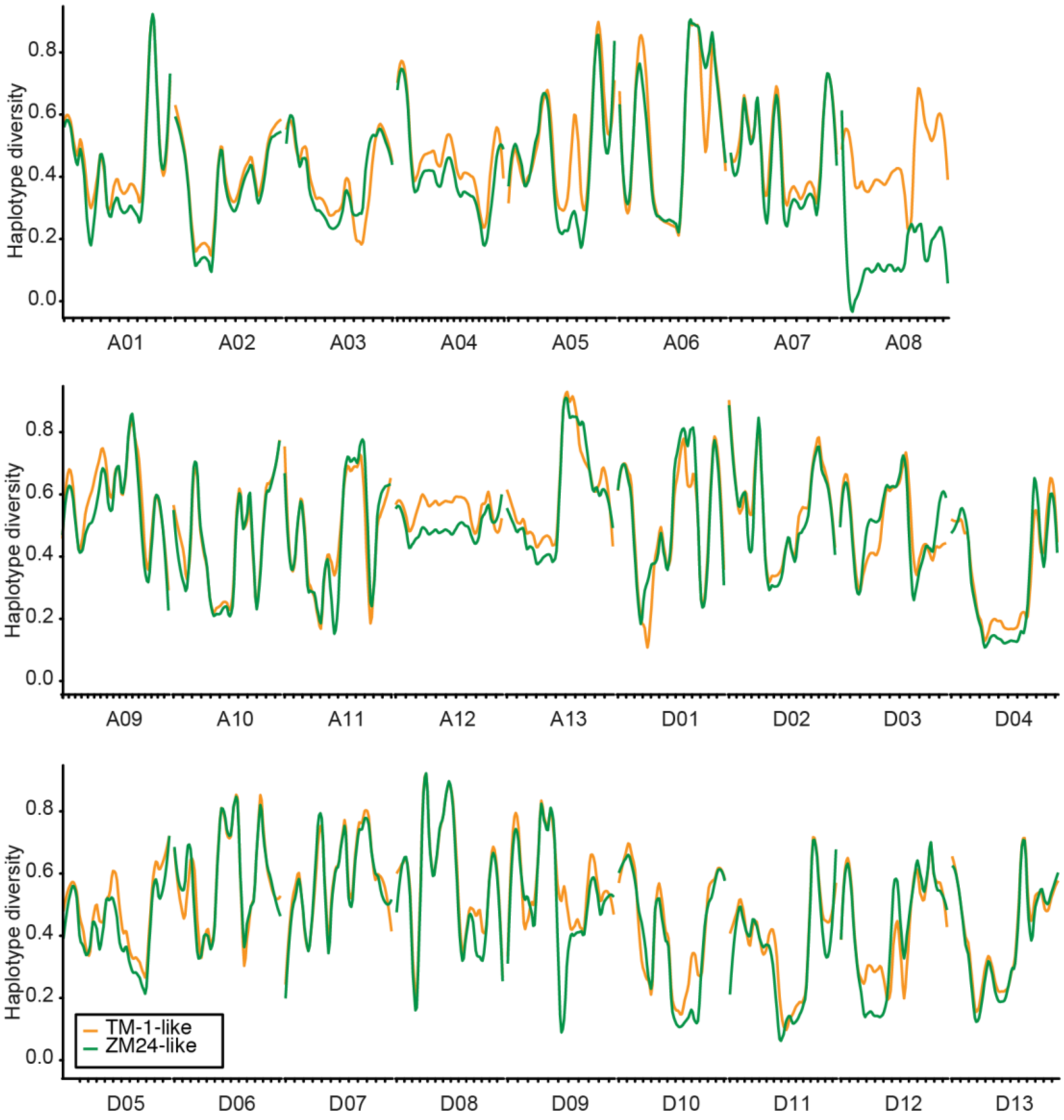

Supplementary Figure 32. Genome-wide haplotype diversity between the two groups of accessions carrying either a TM-1-like or a ZM24-like chromosome-A08 inversion allele. Source data are provided in Source Data file 1.

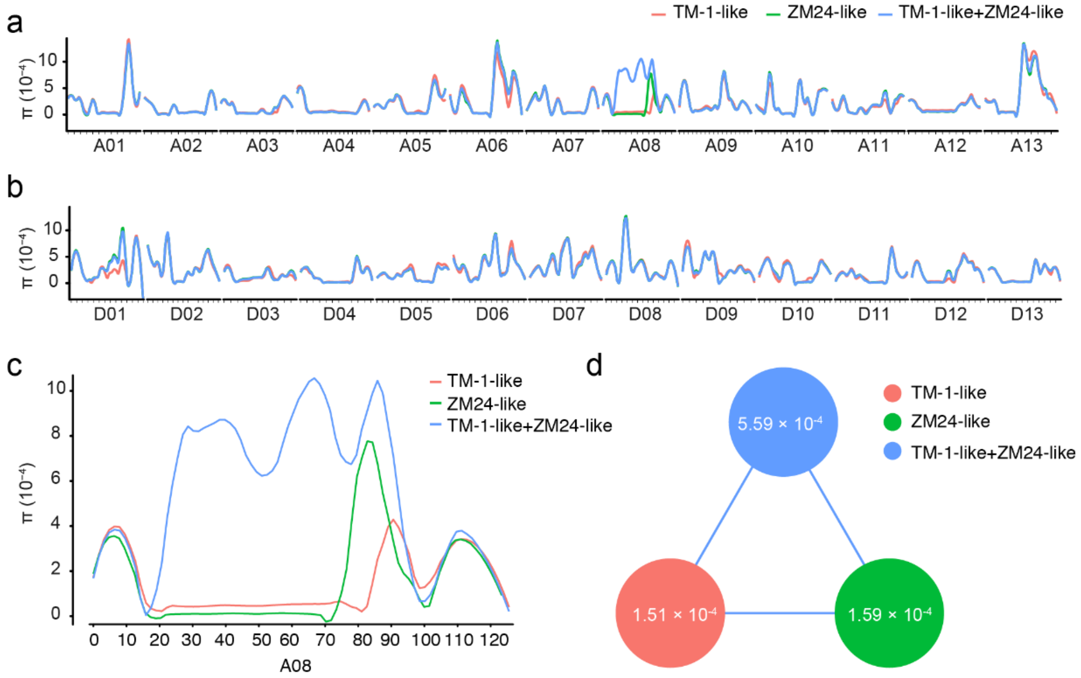

Supplementary Figure 33. Genome-wide genetic diversity analysis. a, Genetic diversity in 13 chromosomes in the A_t subgenome. b, Genetic diversity in 13 chromosomes in the D_t subgenome. c, Significantly decreased genetic diversity in TM-1-like and ZM24-like genotypes groups. d, Average genetic diversity among the TM-like group, the ZM24-like group, and the 419 accessions (TM-1-like+ZM24-like) of the germplasm diversity panel. Source data are provided in Source Data file 1.

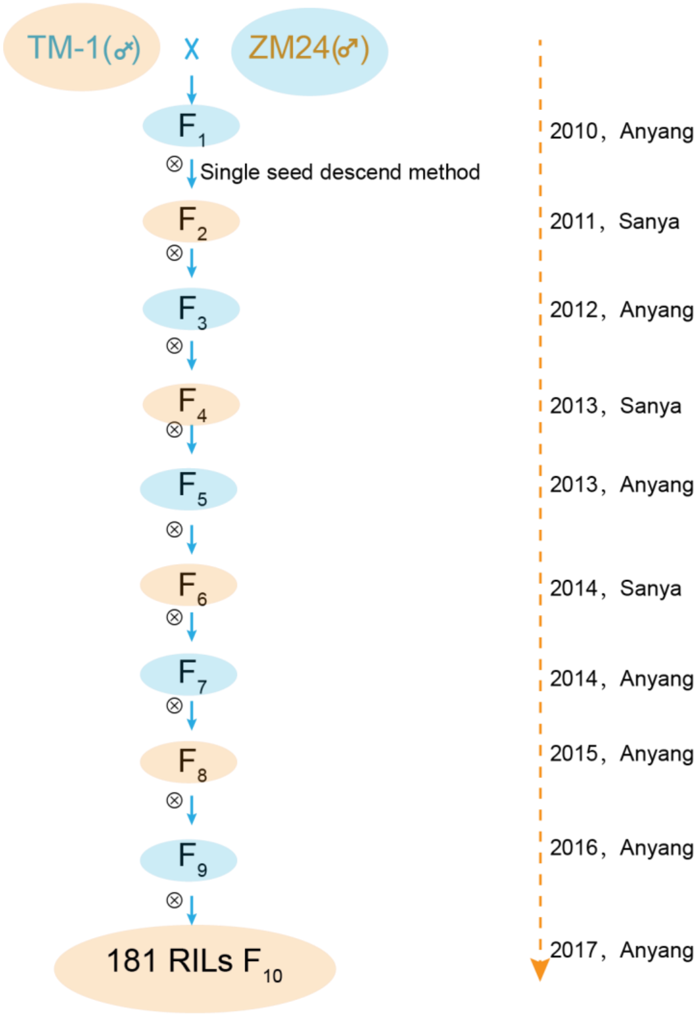

Supplementary Figure 34. Flowchart illustrating the approach for constructing the RILs population. The *G. hirsutum* TM-1 was used as the maternal parent and ZM24 was used as the paternal parent. This RILs population was constructed over eight years (from 2010 to 2017). The final population has 181 sister lines.

Supplementary Table 1. Details of TM-1 and ZM24 genome assemblies

	TM-1		ZM24	
	**Size (Mb)**	**Number**	**Size (Mb)**	**Number**
N10	15.00	13	7.06	26
N20	10.03	32	4.73	66
N30	6.91	60	3.34	124
N40	5.68	97	2.55	204
**N50**	**4.76**	**141**	**1.98**	**308**
N60	3.85	195	1.52	440
N70	3.02	261	1.13	615
N80	2.04	352	0.76	862
**N90**	**1.29**	**490**	**0.42**	**1,263**
Total number	-	1,283	-	3,718
**Anchored and oriented contigs**		**699**		**1,497**
**Total Size (Mb)**	**2,286**		**2,309**	

Supplementary Table 2. Summary of Hi-C data mapping to TM-1 and ZM24 contigs

	Type	ZM24		TM-1	
		Number	Ratio (%)	Number	Ratio (%)
Hi-C	Unique Paired Alignments	548,718,535	100	357,352,320	100
	**Valid Interaction Pairs**	**476,345,174**	**86.81**	**324,195,247**	**90.72**
	Dangling End Pairs	43,901,784	8.00	11,867,422	3.32
	Re-ligation Pairs	6,669,018	1.21	4,066,419	1.14
	Self-cycle Pairs	7,127,016	1.30	10,340,655	2.89
	Dumped Pairs	11,592,643	2.11	6,882,577	1.93

Supplementary Table 3. Comparison of genome assemblies with recently published cotton sequences. This table shows a summary of sequencing platform, assembly strategy and stats for the newly published tetraploid genomes and sequences in this study.

	G. hirsutum TM-1 (ICR)	*G. hirsutum* ZM24 (ICR)	*G.hirsutum* TM-1 (HAU)	*G.barbadense* 3-79 (HAU)
Sequence platform	PacBio RSII	PacBio RSII	PacBio RSII	PacBio RSII
Assembly strategy	PacBio+Hi-C	PacBio+Hi-C	PacBio+Hi-C+BioNano	PacBio+Hi-C+BioNano
Total Scaffold assembly size, Mb	NA	NA	2,347	2,266
**Total Contig assembly size, Mb**	**2,286**	**2,309**	**2,282**	**2,223**
Total scaffold number	NA	NA	2,190	3,032
**Contig N50, Kb**	**4,760**	**1,976**	**1,891**	**2,151**
Scaffold L50, Mb	NA	NA	97	92
Anchored and oriented scaffolds, Mb	NA	NA	2,233	2,133
Anchored and oriented contigs, Mb	2,226	2,150	2,172	2,090
GAPs in pseudochromosomes	673	1,471	2,564	1,900
Number of genes	73,624	73,707	70,199	71,297
Repeat sequence	73.68%	72.14%	69.86%	69.83%
Percentage of anchoring，bp	99.87%	99.41%	98.94%	97.68
**Percentage of anchoring and ordering**	**97.50%**	**93.70%**	**96.16%**	**96.35%**

Supplementary Table 4. Details of TM-1 and ZM24 assembled based on Hi-C sequences

Group	TM-1		ZM24	
	Contig	Sequence Length	Contig Number	Sequence Length
A01	91	119,978,243	168	119,243,513
A02	50	107,919,092	125	108,431,665
A03	70	113,666,453	171	113,743,290
A04	63	88,846,017	130	88,922,991
A05	59	112,633,482	138	113,380,895
A06	59	127,856,816	161	128,025,920
A07	42	98,680,982	145	99,339,544
A08	52	126,428,155	160	126,458,453
A09	40	84,574,461	111	85,613,634
A10	53	117,852,100	158	118,540,607
A11	74	123,804,389	171	124,446,870
A12	79	109,617,758	163	109,608,230
A13	48	111,652,831	124	112,062,747
D01	27	65,754,851	84	66,484,502
D02	36	73,137,787	96	73,942,559
D03	28	54,968,606	58	55,168,097
D04	46	59,212,131	80	59,146,670
D05	28	66,275,588	97	66,583,820
D06	23	66,767,104	60	66,554,709
D07	36	60,304,445	95	60,629,887
D08	34	70,233,655	93	70,715,563
D09	31	54,428,497	72	55,078,203
D10	53	69,211,660	85	69,710,995
D11	29	73,197,459	80	73,978,447
D12	30	63,597,248	73	64,046,377
D13	28	65,310,175	84	65,565,975
**Clustered**	**1,209 (94.23)**	**2,285,909,985**	**2,982 (80.2)**	**2,295,424,163**
**Ordered and Oriented**	**699**	**2,226,441,035 (97.5)**	**1,497**	**2,150,008,364 (93.7)**

Supplementary Table 5. Evaluation of the TM-1 and ZM24 genome assemblies using the BUSCO database.

Taxa	Complete BUSCOs	Single-copy BUSCOs	Duplicated BUSCOs	Fragmented BUSCOs	Missing BUSCOs	Total Lineage BUSCOs
**TM-1 (ICR)**	1,420 (98.61%)	147 (10.21%)	1,273 (88.40%)	5 (0.35%)	15 (1.04%)	1,440
**ZM24 (ICR)**	1,419 (98.54%)	131 (9.10%)	1,288 (89.44%)	7 (0.49%)	14 (0.97%)	1,440
**TM-1 (HAU)**	1415(98.26%)	NA	NA	9(0.62%)	16(1.11%)	1440
**3-79 (HAU)**	1420 (98.61%)	NA	NA	5(0.25%)	15(1.04%)	1440

Supplementary Table 6. Comparison of repetitive elements between TM-1 and ZM24

Type	TM-1			ZM24		
	**Number**	**Length (bp)**	**Percentage of assembly (%)**	**Number**	**Length (bp)**	**Percentage of assembly (%)**
ClassI/DIRS	93,245	159,050,493	6.95	91,627	158,707,484	6.87
ClassI/LINE	9,423	14,173,853	0.62	9,309	13,904,947	0.6
ClassI/LTR	1,736	2,390,711	0.1	1,702	2,351,904	0.1
**ClassI/LTR/Copia**	**128,536**	**170,034,658**	**7.43**	**126,458**	**168,533,745**	**7.3**
**ClassI/LTR/Gypsy**	**464,127**	**952,699,101**	**41.62**	**455,862**	**938,584,840**	**40.65**
ClassI/PLE|LARD	81,355	135,137,172	5.9	79,920	133,315,221	5.77
ClassI/SINE	7,301	1,358,230	0.06	7,171	1,338,227	0.06
ClassI/TRIM	4,438	2,689,791	0.12	4,475	2,715,087	0.12
ClassI/Unknown	1,699	368,450	0.02	1,628	350,329	0.02
ClassII/Crypton	268	141,872	0.01	272	158,170	0.01
ClassII/Helitron	57,673	14,118,378	0.62	57,189	14,102,181	0.61
ClassII/MITE	6,778	1,444,338	0.06	6,654	1,423,071	0.06
ClassII/Maverick	2,279	563,722	0.02	2,270	564,089	0.02
ClassII/TIR	100,890	58,542,077	2.56	99,381	57,820,698	2.5
ClassII/Unknown	3,583	726,122	0.03	3,606	710,376	0.03
Potential Host Gene	50,584	11,435,713	0.5	50,107	11,343,825	0.49
SSR	15,436	2,445,333	0.11	15,268	2,423,592	0.1
Unknown	497,188	159,165,545	6.95	489,675	157,405,326	6.82
**Total without overlap:**	**1,526,539**	**1,686,485,559**	**73.68**	**1,502,574**	**1,665,753,112**	**72.14**

Supplementary Table 7. Summary of one-to-one blocks between *G. arboreum* (A_2) and the A_t subgenomes or between *G. raimondii* (D_5) and the D_t subgenomes

Item	I		II		III		IV	
	D_5	ZM24 D_t	TM-1 D_t	D_5	A_2	ZM24 A_t	TM-1 A_t	A_2
one-to-one	82,879	82,879	84,915	84,915	104,811	104,811	107,185	107,185
Total Length	632,398,520	632,884,704	641,360,965	641,032,907	1,040,580,391	1,039,948,763	1,058,398,495	1,059,324,525
Ratio	84.4%	80.3%	78.1%	85.6%	70.8%	76.3%	75.3%	72.1%
Avg. Length	7,630.38	7,636.25	75,52.98	7,549.11	9,928.16	9,922.13	10,451.74	10,461.42
Avg. Identity (%)	96.48	96.48	96.46	96.46	97.65	97.65	97.48	97.48

Supplementary Table 8. Summary of aligned sequences between TM-1 and ZM24

	**A_t subgenome**		**D_t subgenome**	
	**TM-1 A_t**	**ZM24 A_t**	**TM-1 D_t**	**ZM24 D_t**
one-to-one syntenic blocks	10,274	10,274	6,296	6,296
Total Length	1,338,685,265	1,339,189,017	773,554,992	773,850,293
Ratio	95.2%	98.3%	94.3%	98.1%
Avg. Length	130,298.35	130,347.38	122,864.52	122,911.42
Avg. Identity	99.83	99.83	99.83	99.83

Supplementary Table 9. Summary of SNP, InDels, and structural variations between TM-1and ZM24

Subgenome	Item	TM-1		ZM24	
		**Allelic**	**Non-allelic**	**Allelic**	**Non-allelic**
A_t	Length	**1,293,390,138**	43,397,184	**1,295,173,765**	41,360,332
	SNP	583,801	137,011	583,807	136,846
	InDel	62,479	5,549	251,463	8,194
	Inversion (Num/Len)	-	60 (38,569,856)	-	60 (37,168,270)
	Intra_translocation (Num/Len)	-	148 (1,761,955)	-	148 (1,778,547)
	Inter_translocation (Num/Len)	-	1166 (3,065,373)	-	1166 (3,024,078)
D_t	Length	**761,820,175**	7,857,147	**762,365,914**	7,602,673
	SNP (Num)	323,881	12,559	323,884	11,942
	InDel (Num)	36,850	1,207	139,302	2,307
	Inversion (Num/Len)	-	67 (4,687,926)	-	67 (4,492,054)
	Intra_translocation (Num/Len)	-	86 (1,423,648)	-	86 (1,433,882)
	Inter_translocation (Num/Len)	-	727 (1,728,915)	-	727 (1,696,059)

Supplementary Table 10. Comparison of TEs between structural variation regions and TM-1 A_t subgenome.

Type	1 Mb region			5 Mb region			10 Mb region			TM-1 A_t subgenome		
	Number	Length	Ratio (%)	Number	Length	Ratio	Number	Length	Ratio	Number	Length	Ratio
ClassI/DIRS	11,447	20,620,250	9.62	33,168	59,244,266	9.24	43,456	77,357,739	9.04	64,227	113,968,173	7.89
ClassI/LINE	537	808,287	0.38	1,793	2,609,533	0.41	2,687	4,138,113	0.48	4,961	7,619,501	0.53
ClassI/LTR/Copia	8,566	10,849,529	5.06	27,485	34,792,132	5.43	37,651	48,019,692	5.61	67,832	90,708,336	6.28
ClassI/LTR/Gypsy	54,256	121,089,848	56.48	159,069	352,027,558	54.89	209,530	463,093,319	54.13	316,717	694,250,433	48.09
ClassI/LTR/Unknown	134	138,737	0.06	365	412,423	0.06	473	542,868	0.06	838	1,091,257	0.08
ClassI/PLE|LARD	8,802	15,750,179	7.35	25,620	45,158,536	7.04	33,840	59,880,284	7	51,885	90,345,508	6.26
ClassI/SINE	359	68,849	0.03	1,226	234,687	0.04	1,696	322,818	0.04	3,513	648,769	0.04
ClassI/TRIM	253	130,111	0.06	848	406,733	0.06	1,163	600,030	0.07	2,227	1,113,046	0.08
ClassI/Unknown	81	13,565	0.01	292	61,581	0.01	398	87,469	0.01	878	182,731	0.01
ClassII/Crypton	4	252	0	23	4,594	0	44	8,259	0	130	56,361	0
ClassII/Helitron	2,222	504,246	0.24	7,732	1,813,170	0.28	10,828	2,532,270	0.3	28,893	6,985,173	0.48
ClassII/MITE	277	60,398	0.03	927	186,958	0.03	1,314	272,388	0.03	3,334	711,818	0.05
ClassII/Maverick	111	26,710	0.01	417	104,076	0.02	557	140,841	0.02	1,089	244,804	0.02
ClassII/TIR	5,215	3,327,002	1.55	17,105	10,620,397	1.66	23,569	14,504,055	1.7	49,250	27,897,386	1.93
ClassII/Unknown	162	37,933	0.02	591	125,488	0.02	826	180,206	0.02	1,829	402,504	0.03
Potential Host Gene	2,144	495,026	0.23	7,073	1,659,612	0.26	9,690	2,250,141	0.26	24,938	5,574,463	0.39
SSR	660	102,009	0.05	2,250	347,286	0.05	3,214	501,093	0.06	7,778	1,209,347	0.08
Unknown	31,855	13,416,609	6.26	102,446	41,204,012	6.43	139,051	55,075,787	6.44	261,964	92,203,270	6.39
Total without overlap:	95,230	187,439,213	87.42	285,984	551,012,020	85.92	380,936	729,506,027	85.27	630,319	1,135,210,776	78.64

Supplementary Table 11. Comparison of TEs between structural variation regions and TM-1 D_t subgenome.

Type	1 Mb region			5 Mb region			10 Mb region			TM-1 D_t subgenome		
	Number	Length	Ratio (%)	Number	Length	Ratio	Number	Length	Ratio	Number	Length	Ratio
ClassI/DIRS	4,509	7,358,875	7.58	10,459	16,650,590	6.88	14,741	23,183,811	6.52	28,968	44,963,635	5.34
ClassI/LINE	436	566,437	0.58	1,195	1,654,591	0.68	1,972	2,780,769	0.78	4,425	6,483,251	0.77
ClassI/LTR/Copia	7,045	8,652,342	8.91	18,470	23,023,018	9.51	27,233	33,971,664	9.55	60,642	79,240,147	9.41
ClassI/LTR/Gypsy	22,289	40,497,615	41.7	51,937	92,493,513	38.19	73,352	129,797,206	36.5	147,192	257,505,804	30.58
ClassI/LTR/Unknown	100	133,446	0.14	236	331,803	0.14	380	525,519	0.15	898	1,299,462	0.15
ClassI/PLE|LARD	3,955	6,157,045	6.34	9,426	14,247,663	5.88	13,580	20,042,754	5.64	29,308	43,800,669	5.2
ClassI/SINE	342	67,552	0.07	1,027	204,426	0.08	1,560	306,489	0.09	3,786	708,926	0.08
ClassI/TRIM	200	94,325	0.1	571	262,594	0.11	866	354,186	0.1	2,165	1,458,921	0.17
ClassI/Unknown	63	12,829	0.01	190	36,527	0.02	305	56,446	0.02	821	185,724	0.02
ClassII/Crypton	7	10,218	0.01	40	21,913	0.01	49	29,219	0.01	137	85,251	0.01
ClassII/Helitron	1,957	494,434	0.51	5,859	1,457,057	0.6	9,213	2,145,366	0.6	28,768	7,130,987	0.85
ClassII/MITE	266	55,645	0.06	739	154,401	0.06	1,178	254,247	0.07	3,443	732,239	0.09
ClassII/Maverick	95	24,032	0.02	277	66,935	0.03	432	106,311	0.03	1,190	318,924	0.04
ClassII/TIR	4,432	2,929,653	3.02	12,666	8,148,874	3.36	19,941	12,921,928	3.63	51,620	30,641,864	3.64
ClassII/Unknown	134	25,887	0.03	350	68,010	0.03	596	114,182	0.03	1,750	323,209	0.04
Potential Host Gene	1,807	405,793	0.42	5,427	1,216,103	0.5	8,574	2,131,651	0.6	25,618	5,854,941	0.7
SSR	594	93,853	0.1	1,706	263,467	0.11	2,663	418,961	0.12	7,652	1,235,144	0.15
Unknown	24,510	7,713,989	7.94	65,533	20,036,087	8.27	98,357	29,570,871	8.31	234,841	66,616,828	7.91
Total without overlap:	48,231	75,293,832	77.52	120,575	180,337,180	74.46	176,635	258,711,015	72.74	398,383	548,584,660	65.14

Supplementary Table 12. Comparison of PAVs on pseudochromosomes between TM-1 and ZM24

Chromosomes	ZM24		TM-1	
	Number	Length	Number	Length
A01	265	45,712	72	1,160,508
A02	307	109,334	74	1,033,131
A03	188	26,327	48	527,194
A04	321	77,872	69	1,041,371
A05	511	65,395	78	1,422,303
A06	225	117,865	74	676,200
A07	272	240,925	115	1,019,576
**A08**	**1847**	**1,158,915**	**465**	**7,868,829**
A09	318	116,135	81	1,143,741
A10	514	2,032,119	419	1,513,722
A11	389	2,684,120	503	1,296,776
A12	353	2,392,639	440	1,193,979
A13	563	3,222,697	640	2,117,471
D01	433	1,747,899	454	1,274,822
D02	443	2,219,857	466	1,425,340
D03	244	1,275,645	293	709,739
D04	194	1,062,140	218	538,942
D05	264	1,827,401	400	884,454
D06	382	2,015,195	462	1,106,089
D07	263	1,473,455	298	895,298
D08	264	1,786,325	352	1,046,463
D09	267	917,565	204	765,833
D10	342	1,434,162	276	1,170,367
D11	741	2,975,653	845	2,275,916
D12	227	1,605,922	321	616,920
D13	213	1,379,089	286	663,486
Contigs	2,810			32,788,393
**Total**	**13,160**	**34,010,363**	**7,953**	**68,176,863**

Supplementary Table 13. Orthologous pairs comparison between TM-1 and ZM24. The A^+ and D^+ represent the gene present in the A_t subgenome and D_t subgenome, respectively. A^- and D^- represent the gene loss from the A_t subgenome and D_t subgenome, respectively.

	**Item**	**ZM24**			**Total number**
		**A^+D^+**	**A^+D^-**	**A^-D^+**	
**TM-1**	A^+D^+	58,913	513	458	59,884
	A^+D^-	433	5,570	30	6,033
	A^-D^+	589	68	5,400	6,057
	**Total number**	**59,935**	**6,151**	**5,888**	**71,974**

Supplementary Table 14. Summary of gene duplication type information for the TM-1 and ZM24 genomes. WGD represents whole genome duplication. SD represents segmental duplication.

Duplication type	TM-1				ZM24			
	A_t	Percentage (%)	D_t	Percentage (%)	A_t	Percentage (%)	D_t	Percentage (%)
Singleton	3,228	9.01	3,304	9.1	3,443	10.26	3,495	9.85
Dispersed	4,365	12.18	5,275	14.53	4,665	13.9	5,537	15.61
Proximal	726	2.03	870	2.4	803	2.39	803	2.26
Tandem	2,376	6.63	2,745	7.56	2,442	7.28	2,442	6.88
WGD or SD	25,140	70.15	24,098	66.4	22,193	66.15	23,193	65
**Total**	**35,835**	**100**	**36,292**	**100**	**33,546**	**100**	**35,470**	**100**

Supplementary Table 15. Comparison of the SNPs between TM-1-like and ZM24-like genotype groups on A08 using the accessions noted in Figure 3D.

**Chr**	**Start**	**End**	**Common SNP**	**TM-1-like Private SNP**	**ZM24-like Private SNP**	**SNPs in 421 accessions panel**	
A08	1	29,760,605	13,053	6,474	2,306	43,163	
**A08**	**29,760,606**	**47,296,785**	**330**	**3,165**	**1,023**	**58,967**	**SV1**
A08	47,296,786	48,145,612	11	161	42	2,349	
**A08**	**48,145,613**	**52,751,556**	**41**	**880**	**185**	**10,431**	**SV2**
A08	52,751,557	61,196,692	291	1,706	619	26,827	
**A08**	**61,196,693**	**75,890,534**	**322**	**3,171**	**809**	**58,110**	**SV3**
A08	75,890,535	125,568,199	27,437	15,440	21,035	92,927	
Total SNPs			41,485	30,977	26,019	292,774	

Supplementary Table 16. Comparison of the SNPs between TM-1-like groups and equal number of random selected accessions from ZM24-like groups on A08. The results were similar with those in Supplementary Table 23, indicating that the population size effect on SNPs numbers can be ignored in our study.

Chr	Start	End	Common SNP	TM-1-like Private SNP	ZM24-like Private SNP	
A08	1	29,760,605	12,754	6,773	2,672	
**A08**	**29,760,606**	**47,296,785**	**349**	**3,146**	**1,195**	**SV1**
A08	47,296,786	48,145,612	14	158	52	
**A08**	**48,145,613**	**52,751,556**	**35**	**886**	**193**	**SV2**
A08	52,751,557	61,196,692	271	1,726	750	
**A08**	**61,196,693**	**75,890,534**	**271**	**3,222**	**903**	**SV3**
A08	75,890,535	125,568,199	27,397	15,480	21,749	

Supplementary Table 17. Primers were used to validate the breakpoints for SV1 and SV3 in TM-1 and ZM24.

Breakpoint validation	Primer name	Primer sequences (5’ -3’)
Left breakpoint in SV1	common fwd	GAACAGATTGATATTTTAGCTCGGG
	TM-1 rev	CCATGCAATAATACGAAATCCACAT
	ZM24 rev	CACCCAATTTGAGCAGTAGTGTTTT
Right breakpoint in SV1	TM-1 fwd	GTTGTTTCCCCAAGTCTAGGTATTAC
	ZM24 fwd	GTTGTTTCCCCAAGTCTAGGTATTAC
	common rev	CCTGGATCAACACATTCGTATA
Left breakpoint in SV3	common fwd	GTATGATTGAATGGTGATTATCACAT
	TM-1 rev2	AAGTCGAGGTGGCGGTCTAAAA
	ZM24 rev2	CTCAATAGAATACCAATCAATTTACC
Right breakpoint in SV3	TM-1 fwd2	GGATTATAAGTTTTTGAAGTCTCCCC
	ZM24 fwd2	TGTTGTGATTGATCGGTAATGCCTC
	common rev	TAGATAACTTTTGGAGTCAAAAGTAG

Supplementary References

1. Zhang, T. et al. Sequencing of allotetraploid cotton (*Gossypium hirsutum* L. acc. TM-1) provides a resource for fiber improvement. Nat. Biotechnol. 33, 531–537 (2015).

2. Wang, S. et al. Sequence-based ultra-dense genetic and physical maps reveal structural variations of allopolyploid cotton genomes. Genome Biol. 16, 108 (2015).
