# Supplementary material for: Extensive intraspecific gene order and gene structural variations in upland cotton cultivars
Source: Nat Commun. 2019 Jul 5;10:2989. doi: 10.1038/s41467-019-10820-x (PMC6611876; doi:10.1038/s41467-019-10820-x)
Supplement: Supplementary file 4 — Description of Additional Supplementary Files [file 41467_2019_10820_MOESM4_ESM.docx]

Description of Additional Supplementary Files

File Name: Supplementary Data 1
Description: Alignments of BAC sequences against TM-1 genome. These BAC sequences were downloaded from NCBI (<https://www.ncbi.nlm.nih.gov/nuccore/>).

File Name: Supplementary Data 2
Description: Alignments of BAC sequences against the ZM24 genome assembly. These BAC sequences were downloaded from NCBI (<https://www.ncbi.nlm.nih.gov/nuccore/>).

File Name: Supplementary Data3
Description: Genomic rearrangement details between the diploid A2 genome and TM-1 At subgenome.

File Name: Supplementary Data 4
Description: Genomic rearrangement details between D5 and the TM-1 Dt subgenome.

File Name: Supplementary Data 5
Description: ZM24 PAV genes and their likely orthologs in ancestor diploid cotton. The prefix Ga represents genes from G. arboreum and Gorai represents G. raimondii. NA，not applicable.

File Name: Supplementary Data 6
Description: TM-1 PAV genes and their likely orthologs in ancestor diploid cotton. The prefix Ga represents genes from G. arboreum and Gorai represents G. raimondii. NA，not applicable.

File Name: Supplementary Data 7
Description: KEGG analysis of TM-1 genes amongst orthologous pairs located in nonsyntenic regions.

File Name: Supplementary Data 8

Description: Summary of the genes located within the TM-1 A08 inversion regions.

File Name: Supplementary Data 9

Description: Summary of the genes located within the ZM24 A08 inversion regions.
