# Supplementary material for: Extensive intraspecific gene order and gene structural variations in upland cotton cultivars
Source: Nat Commun. 2019 Jul 5;10:2989. doi: 10.1038/s41467-019-10820-x (PMC6611876; doi:10.1038/s41467-019-10820-x)
Supplement: Supplementary file 2 — Peer Review [file 41467_2019_10820_MOESM2_ESM.pdf]

Reviewers' comments:

Reviewer #1 (Remarks to the Author):

In the present MS, they assembled the genomes of G. hirsutum acc. TM-1 and cv. zhongmiansuo24 (ZM24) using single-molecule long read and Hi-C sequencing technologies. A total of 127 inversions and 2,127 translocations were identified between TM-1 and ZM24. A large-scale inversion on chromosome A08 is identified. This inversion is widely distributed through a panel of germplasm haplotype analyses and has over time mediated the reduction of meiotic recombination. These new upland cotton genome assemblies are likely to become the new benchmark for cotton functional genomics research. However, I think the present version cannot be accepted due to following my concern.

1. There are too many grammatical mistakes in English writing (e.g. verbs used as nouns, mix up of singular/plural forms) that need to be corrected. For example, there are three word spelling mistakes in one table title "Supplemetary Table 5. Comparsion of repetitive elments between TM-1 and ZM24". I cannot understand why such primary draft has submitted for reviewing.

2. No evaluation of the assembly quality by alternative methods. All the different assembly methodologies (both single-molecule long read and Hi-C sequencing technologies) were used to produce a high quality genome assembly but none of them were used to evaluate how good the assembly quality was. From their poor quality assemble of genomes of diploid A and D species and tetraploid cotton, I really want to know how they evaluate their genome assemble quality and present their comparison results with previous TM-1 genome including their own genome.

3. Yes, it is important ZM24 is widely used in transgenic crop improvement and research programs in this lab. Due to genotype dependency, only a few lines of accessions (Coker 312 or its derivative) are suitable for transformation as an explant in cotton. The cotton transgenic operation is still time consuming. Since you sequenced ZM24 (I think that is why you sequence this cultivar) and developed (TM-1 ZM24)RILs, we hope to know why there is no transgenic barrier in ZM24, or that probably some functional validation of the candidate genes is behind this transforming traits, otherwise no useful to sequence this genome.

4. Transcriptomic experiments looks like are not supported by biological replicates. It's certainly highly valuable dataset to get a gene set that is as complete as possible.

5. The two references are estimated to cover more than 99% of the upland cotton genome? With the aid of Hi-C data, we anchored and oriented ~2.23 Gb of the assembly onto 26 pseudochromosomes of TM-1 (Supplementary Fig. 1), which represented 97.4 % of the total assembly. 2.15 Gb (93.7%) of the assemblies from ZM24 were anchored and oriented onto 26 pseudochromosomes (Supplementary Fig. 2 and Supplementary Table 3). I do not know how you measure the genome size. The genome size should be measured also using flow cytometry.

6. The expansion of LTR retrotransposons in cotton occurred mainly within the past 2 million years (MYA) in both the TM-1 and ZM24 At subgenomes (Supplementary Fig. 4), and that in TM-1 and ZM24 Dt subgenome occurred mainly within the past 1 MYA, in agreement with our LTR divergency results. We know Tetraploid cotton originated in the New World from the trans-oceanic hybridization of an A-genome-like ancestral African species, G. herbaceum or G. arboreum, with a native D-genome-like species, G. raimondii (D5) ~1-1.5 million years ago (MYA). So, do you find the expansion of LTR retrotransposons in A and D diploid cotton species?

7. Many genome or subgenome and species-specific genes have been identified, however, no further function analysis is provided. For example, 251 and 149 gene pairs from At- or Dt subgenome were identified in the nonsyntenic regions; these accounted for ~0.7 % and ~0.5% of the total analyzed gene pairs for the At- and Dt- subgenomes, respectively. We hope to know what their functions are through KEGG analysis.

8. Many mistakes still in Reference: what is the following? Nature Publishing Group 1–18 412 (2018). doi:10.1038/s41588-018-0119-7

9. To explore these ideas experimentally, they generated a population of 181 RILs by crossing TM-1 and ZM24, which were used to calculate recombination frequencies. The authors should describe in detail how develop and use the genetic map in genome assembly.

10. The authors should summarize how many genes are there in the inversions on A08?

11. The authors did not characterized possible gene conversions and homoelogs exchanges between the A and D subgenomes in the tetraploid cotton. So, we do not know if their identified introgressions come from HE or introgression.

12. Data availability. Not only TM-1 and ZM24 assembly and annotation data, but also how other data make publically available.

Reviewer #2 (Remarks to the Author):

The ms by Yang et al on 'extensive intraspcific gene order and gene structural variation in upland cotton' describes genome comparisons between two cotton varieties and in relation to progenitor species based on new genome assemblies. They find large-scale inversions, translocations, SNPs, InDels and presence-absence variation.

After their previous publications on the genome of Gossypium (Du et al., 2018, Wang et al., 2012 and Li et al., 2014) this group (Institute of Cotton Research, Anyang) has again made progress in getting more detailed analysis of the genome of cotton. However, the question arises how much more relevant information is generated in this study. The authors present a large amount of genomic differences between the cotton accessions TM-1 and ZM24, but the conclusions are meager. This is the major problem of this manuscript.

In the abstract the authors state "….. the scientific insights from our comparative analyses contributed substantially to our basic understanding of how genomic inversions influence meiotic recombination and thus lower genetic diversity in plant populations" (L28 - L31). What exactly is this 'basic understanding' that this work provides? Is that novel information? Actually, this paper does not prove or show the relation between inversion and genetic diversity and/or does not explain the correlation. Such a claim requires more extensive research with e.g. GWAS studies. The authors could discuss which of the cotton accessions have a different Lint percentage in relation to the A08 inversion region? This is a missed opportunity. This should also be discussed in relation to the presence of the Lint QTLs on D08.

In the first four results paragraphs (L90 - L282) the text is extremely descriptive with many percentages and an impressive number of figures and tables demonstrating the genetic variation. This

part is of interest only for specialized readers. The last paragraph of the Results (L284 - L338) provides more phylogenetic and biological arguments. The authors conclude that the inversion on chromosome A08 of the tetraploid cotton can discriminate two major haplotypes in 421 accessions. Furthermore, they claim that the ZM24-like group of accessions has an ecological advantage based on the presence of the A08 inversion. These issues could and should be much more elaborated.

other issues:
- Considering the large size (megabases) of the inversion regions of A08, it is surprising that there are few crossovers (right flank of SV3 in Fig 3h). This could be explained. Also, the size of the region with suppressed recombination (~ 10Mb) flanking the left breakpoint of SV1 (Fig. 3h) needs explanation.
- L102: Ref. 22 did not use Hi-C
- L129-130: It is not clear of which fraction/group the presented percentages are taken.
- L141: What do these numbers mean? The relevance should be clearer.
- L301: What is meant by 'closely linked' in this sentence?
- L348-355: This sentence is too long and incomprehensible.
- L867: 'd,' should be removed.

Reviewer #3 (Remarks to the Author):

In the manuscript by Yang et al, the authors generate long-read assemblies of two upland cotton cultivars and use these data for comparative analyses with the parental diploids to draw conclusions regarding genome evolution after whole genome duplication. The authors conclude that major genomic rearrangement has led to reproductive isolation and divergence of the two cultivars and, through phylogenetic analysis of these regions in the diversity panel, show that this rearrangement corresponds to the evolutionary relationships of included taxa. I believe the molecular work and bioinformatic analyses (with minor exceptions) to be robust, although not necessarily presented as well as it could be. Specific comments as they appear in the manuscript:

142-146: The transposable element analysis appears to be thrown in for no reason. Perhaps to demonstrate the quality of the assembly by comparison to other analyses of TE composition in cotton? Additionally, I question the methods used to estimate insertional timing. The authors state that the D subgenome amplification occurred within the last 1my and the A subgenome within the last 2my; however, these subgenomes co-exist within the same nucleus, so one should expect insertions to occur randomly throughout the entire genome.

148: "random changes" - do we really know that these rearrangements are random?

pgs 10-11: I really like figure 1. I was disappointed that there was no discussion of the evolutionary implications of the rearrangements depicted here. Since ZM24 and TM-1 contain the same rearrangements (mostly), and this is a very young polyploid, it is interesting to note that most of these probably occurred very quickly after the genome doubling event, pre-divergence of the two cultivars. Theories regarding structural rearrangement during polyploid stabilization could have been discussed in depth in the discussion section. Like the TE analysis, this was an interesting topic that was mostly just skipped over.

222-226: Was there any verification performed for a subset of these variants? One bp indels could very well be sequencing and assembly errors. The authors did not use an extremely robust trim and filter protocol for the short reads, and PacBio is relatively error prone.

232: The title for this section seems like a stretch. I would not call these finding for gene order "extensive" by any means. In addition, the results for mutations and other types of variants are included in this section. It was hard to follow exactly what "gene pairs" and "genes" the authors were

discussing. This section was a bit confusing.

269-271. "...these variations likely represent a substantial portion of the phenotypic differences between these two accessions". This is a stretch and completely ignores the major effects of expression variation and epigenetic modification on phenotypic differentiation. The authors do not present any evidence to support this statement.

Discussion section: This entire section was disappointing. In addition to the writing, which was difficult to decipher, the authors mostly discuss the quality of the assemblies and how they will be a valuable resource to the community. It contains relatively few biological insights.

Figures: The figures are great. My only comment is for figure 2 (the circos plot). I can barely see the structural variants. The inversion is very clear, but I can barely see the intra translocation and cannot tell if there is an inter translocation (although I am assuming there is since it is in the legend).

There are numerous typos and grammatical issues throughout.

NARRATIVE SUMMARY and HIGHLIGHTS

We would first like to thank the Editor and the Referees for their helpful and constructive input about our manuscript. As we trust you'll agree, both our study and the manuscript have been significantly improved in the course of our revision process. We believe that the current version should now be suitable for publication in Nature Communications. Again, many thanks for your ongoing work on our behalf.

Before getting into the full point-by-point response (below), we would like to very briefly summarize the key aspects of our new analyses and our revision of the manuscript:

—We have used both genetic maps and the publicly available completely sequenced bacterial artificial chromosome sequences to evaluate both genomes, and thusly bolstered our confidence in the quality of the new assemblies

—We extensively compared our new genome assemblies with the previously published TM-1 reference sequence

—We conducted KEGG analysis (annotation and enrichment) of the genes in nonsyntenic regions

—We extensively explored the TE content around the breakpoints of the chromosomal rearrangements that we discovered, and investigated TEs expansion in two diploid cottons (A and D).

POINT-BY-POINT RESPONSES

Reviewers' comments:

Reviewer #1 (Remarks to the Author):

In the present MS, they assembled the genomes of G. hirsutum acc. TM-1 and cv. zhongmiansuo24 (ZM24) using single-molecule long read and Hi-C sequencing technologies. A total of 127 inversions and 2,127 translocations were identified between TM-1 and ZM24. A large-scale inversion on chromosome A08 is identified. This inversion is widely distributed through a panel of germplasm haplotype analyses and has over time mediated the reduction of meiotic recombination. These new upland cotton genome assemblies are likely to become the new benchmark for cotton functional genomics research. However, I think the present version cannot be accepted due to following my concern.

1. There are too many grammatical mistakes in English writing (e.g. verbs used as nouns, mix up of singular/plural forms) that need to be corrected. For example, there are three word spelling mistakes in one table title “Supplemetary Table 5. Comparsion of repetitive elments between TM-1 and ZM24”. I cannot understand why such primary draft has submitted for reviewing.

Answer: Thanks very much. We have invited a native English-speaking American with a Ph.D degree in Plant Biology to read and revise the whole manuscript for us. All his corrections about grammar and usage are tracked in the text.

2. No evaluation of the assembly quality by alternative methods. All the different assembly methodologies (both single-molecule long read and Hi-C sequencing technologies) were used to produce a high quality genome assembly but none of them were used to evaluate how good the assembly quality was. From their poor quality assemble of genomes of diploid A and D species and tetraploid cotton, I really want to know how they evaluate their genome assemble quality and present their comparison results with previous TM-1 genome including their own genome.

Answer: Thank you for this comment, which prompted us to undertake a number of evaluations. Specifically, we used the previously published genetic map, and completely sequenced bacterial artificial chromosome sequences to evaluate our assemblies. Both genomes were compared with the genetic maps, and the results indicate high consistency for each chromosome (**Supplementary Figs. 5–8; Page 7 line 117-119**). The perfect matches of 58 completely sequenced bacterial artificial chromosome sequences against both genomes further underscore the accuracy and completeness of both assemblies (**Supplementary Tables 4 and 5; page 7 line 120-122**). We also performed a comparison between TM-1 in this study and previously a published genome (NAU) using mapping Hi-C data to NAU-TM-1 and made direct alignments between the two genome sequences (**Supplementary Figs. 3 and 4; Page7, line 114**).

3. Yes, it is important ZM24 is widely used in transgenic crop improvement and research programs in this lab. Due to genotype dependency, only a few lines of accessions (Coker 312 or its derivative) are suitable for transformation as an explant in cotton. The cotton transgenic operation is still time consuming. Since you sequenced ZM24 (I think that is why you sequence this cultivar) and developed (TM-1 ZM24)RILs, we hope to know why there is no transgenic barrier in ZM24, or that probably some functional validation of the candidate genes is behind this transforming traits, otherwise no useful to sequence this genome.

Answer: We appreciate the point the reviewer is making here. The process through which a single somatic cell becomes a whole plant is a significantly complex question in plant science research. It will be fascinating if we are eventually able to demonstrate how ZM24 differs from other lines in terms of transgenic barriers, yet at present this remains an unsolved issue. In the community of cotton research, only the TM-1 reference is currently available to the public. Thus, our work with a

different line opens a whole new era for comparative cotton genomics. We trust that the reviewer appreciates how the availability of diverse genome sequences is foundational for many of the standard techniques underlying modern life science research in the genomics era.

4. Transcriptomic experiments looks like are not supported by biological replicates. It's certainly highly valuable dataset to get a gene set that is as complete as possible.

Answer : To clarify, the transcriptomic experiments (Iso-seqs) that we conducted were used for gene prediction, not for a typical functional-genomics type biological study. Given our goal with these experiments, we used different tissues and organs to represent as many transcripts as possible; the idea of biological replicates is not germane to Iso-seq approaches.

5. The two references are estimated to cover more than 99% of the upland cotton genome? With the aid of Hi-C data, we anchored and oriented ~2.23 Gb of the assembly onto 26 pseudo-chromosomes of TM-1 (Supplementary Fig. 1), which represented 97.4 % of the total assembly. 2.15 Gb (93.7%) of the assemblies from ZM24 were anchored and oriented onto 26 pseudo-chromosomes (Supplementary Fig. 2 and Supplementary Table 3). I do not know how you measure the genome size. The genome size should be measured also using flow cytometry.

Answer: Thanks very much for this comment; we agree that the statement "99% of the upland cotton" is not accurate, as you point out. We had been using the percentages relative to the length of the total contig assembly. Guided by your comment, we have now revised this to read as "The anchored ~2,286 Mb and ~2,295 Mb of the assemblies onto 26 pseudochromosomes of TM-1 and ZM24 are estimated to cover more than 99% of the total sequences in the assemblies." (**Page 5, line 76-80**).

6. The expansion of LTR retrotransposons in cotton occurred mainly within the past 2 million years (MYA) in both the TM-1 and ZM24 At subgenomes (Supplementary Fig. 4), and that in TM-1 and ZM24 Dt subgenome occurred mainly within the past 1 MYA, in agreement with our LTR divergency results. We know Tetraploid cotton originated in the New World from the trans-oceanic hybridization of an A-genome-like ancestral African species, G. herbaceum or G. arboreum, with a native D-genome-like species, G. raimondii (D5) ~1-1.5 million years ago (MYA). So, do you find the expansion of LTR retrotransposons in A and D diploid cotton species?

Answer: We identified 33,908 and 6,933 high-confidence full-length LTRs from the A and D diploid cotton progenitor species; these were used to evaluate the LTR insertion time using the EMBOSS software package. Like the A_t subgenomes, we found that A genome also experienced expansion within the past 2 million years (MYA). Compared with the D_t subgenome, a relative lower percentage of young LTRs were identified in the D genome. We noticed that the full-length

LTRs in the D genome were smaller than those in the D_t subgenome, which likely results from the collapsed LTRs in the assembly of *G. raimondii* (because it was assembled based on short-reads). Liu *et al.* (2001) concluded that allopolyploid formation has not been associated with sudden and large increases in retroelement activity or with changes in retroelement methylation status[44]. So, the LTR insertion into the diploid D genome should be similar with the LTR insertion into the D_t subgenomes. We also found that the young LTR insertion time pre- (A genome) and after polyploidization (A_t subgenomes) exhibited no difference, indicating that most LTRs expanded in the progenitor genomes before polyploidization and were retained after allopolyploid formation (**Page 9, line150-152, line 155-163**).

7. Many genome or subgenome and species-specific genes have been identified, however, no further function analysis is provided. For example, 251 and 149 gene pairs from At- or Dt subgenome were identified in the nonsyntenic regions; these accounted for ~0.7 % and ~0.5% of the total analyzed gene pairs for the At- and Dt- subgenomes, respectively. We hope to know what their functions are through KEGG analysis.

Answer: We have added the requested newly performed KEGG analysis in Supplementary Figs. 18 and 19 and Supplementary Table 19. We found that these genes were enriched in several metabolism pathways including Vitamin B6 metabolism and glycosaminoglycan degradation (**Page 15, line 282 to 284**).

8. Many mistakes still in Reference: what is the following? Nature Publishing Group 1–18 412 (2018). doi:10.1038/s41588-018-0119-7

Answer: Thanks very much. We have adjusted the reference according to the style for *Nature Communications*. See Reference section (**Line 823-1023**).

9. To explore these ideas experimentally, they generated a population of 181 RILs by crossing TM-1 and ZM24, which were used to calculate recombination frequencies. The authors should describe in detail how develop and use the genetic map in genome assembly.

Answer: Thanks very much. We have added a flowchart to show how we generated the RILs population in supplementary Fig. 31 (**Page 43, line 558**). The genetic map was only used to detect the genetic exchange in the inversion regions on A08.

10. The authors should summarize how many genes are there in the inversions on A08?

Answer: Thanks. Addressing this point, the gene lists within TM-1 and ZM24 A08 inversions are summarized in Supplementary Tables 15 and 16. Specifically, we identified 226 and 248 genes from ZM24 and TM-1, respectively (**Page 19, line 350 to line 352**).

11. The authors did not characterize possible gene conversions and homoelogs exchanges between the A and D subgenomes in the tetraploid cotton. So, we do not know if their identified introgressions come from HE or introgression.

Answer: We identified the syntenic blocks using MCScan among *G. hirsutum*, *G. arboreum* and *G. raimondii* (with default parameters). We then identified the orthologous pairs between each of the sub-genome of *G. hirsutum* (with each of the two ancestor genomes). Ks values for each pair were calculated using yn00 in PAML. We here defined HE based on the method from Li *et al.*[12]. We finally identified 983 possible HE regions, of which 546 were D_t to A_t and 437 were A_t to D_t. Further analysis of possible HEs indicated that 575 genes were transferred from D_t to A_t , whereas 469 genes were transferred from A_t to D_t.

12. Data availability. Not only TM-1 and ZM24 assembly and annotation data, but also how other data make publically available.

Answer: The genome assemblies, gene annotations, RNA-seq data, and other supporting data are accessible through ftp://bioinfo.ayit.edu.cn/downloads/. All raw sequencing data for TM-1 and ZM24 have been deposited in the NCBI BioProject database under accession number PRJNA503326 (**Page 43, line 817-820**).

Again, we would like to thank Reviewer #1 for their constructive comments.

Reviewer #2 (Remarks to the Author):

The ms by Yang et al on ‘extensive intraspcific gene order and gene structural variation in upland cotton’ describes genome comparisons between two cotton varieties and in relation to progenitor species based on new genome assemblies. They find large-scale inversions, translocations, SNPs, InDels and presence-absence variation.

After their previous publications on the genome of Gossypium (Du et al., 2018, Wang et al., 2012 and Li et al., 2014) this group (Institute of Cotton Research, Anyang) has again made progress in getting more detailed analysis of the genome of cotton. However, the question arises how much more relevant information is generated in this study. The authors present a large amount of genomic differences between the cotton accessions TM-1 and ZM24, but the conclusions are meager. This is the major problem of this manuscript.

In the abstract the authors state "….. the scientific insights from our comparative analyses contributed substantially to our basic understanding of how genomic inversions influence meiotic recombination and thus lower genetic diversity in plant populations" (L28 - L31). What exactly is this 'basic understanding' that this work provides? Is that novel information? Actually, this paper does not prove or show the relation between inversion and genetic diversity and/or does not explain the correlation. Such a claim requires more extensive research with e.g. GWAS studies. The authors could discuss which of the cotton accessions have a different Lint percentage in relation to the A08 inversion region? This is a missed opportunity. This should also be discussed in relation to the presence of the Lint QTLs on D08.

Answer: Thanks very much bringing this deficiency to our attention. We have now added analysis of the "common" and "private" SNPs between TM-1-like and ZM24-like genotypes groups. This exciting analysis revealed that a large amount of private SNPs are harbored by both the TM-1-like and ZM24-like genotypes, indicating that the recombination is restricted in the inverted regions and this has resulted in decreased genetic diversity (**Page 21, line 382-390**). Further, genetic diversity analysis showed that TM-1-like genotypes and ZM24-like genotypes showed significantly reduced genetic diversity within the inversion regions, again supporting that the inversions reduced genetic diversity (**Page 21, line 391-396**). In the substantially reworked discussion section, we now mention the QTLs in relation to fiber lint percentage for A08 (**Page 26, line 479-486**). Ma et al[14] published a core collection of upland cotton in 2018, but they did not offer the phenotype data for lint percentage in the supplementary materials.

In the first four results paragraphs (L90 - L282) the text is extremely descriptive with many percentages and an impressive number of figures and tables demonstrating the genetic variation. This part is of interest only for specialized readers. The last paragraph of the Results (L284 - L338) provides more phylogenetic and biological arguments. The authors conclude that the inversion on chromosome A08 of the tetraploid cotton can discriminate two major haplotypes in 421 accessions. Furthermore, they claim that the ZM24-like group of accessions has an ecological advantage based on the presence of the A08 inversion. These issues could and should be much more elaborated.

Answer: Thanks very much. We added the references about the relationship between inversion and local adaptation in the discussion section, and discussed the possible effect of inversion from A08 on ecological adaptation (**Page 25 line 456 to 467**).
other issues:

- Considering the large size (megabases) of the inversion regions of A08, it is surprising that there are few crossovers (right flank of SV3 in Fig 3h). This could be explained. Also, the size of the

region with suppressed recombination (~ 10Mb) flanking the left breakpoint of SV1 (Fig. 3h) needs explanation.

Answer: Thanks very much. A previous study[42] showed that recombination suppression of inversions extends beyond breakpoints: markers outside inverted regions tend to have heightened divergence, and this phenomenon was observed in our study as shown in Fig. 3f (**Page 20-21, line 378 to 381**).

- L102: Ref. 22 did not use Hi-C

Answer: Thanks for correcting the error; we have removed this reference (**Page 4, line 62).**

- L129-130: It is not clear of which fraction/group the presented percentages are taken.

Answer: Thanks very much for this comment. All of the percentages given are relative to the total amount of sequence in the assembly; we have clarified this in the main text. For example, the relevant sentence now reads "Among these, retrotransposons represented 62.8% and 61.5% of all sequences in the TM-1 and ZM24 assemblies. Long terminal repeats (LTR) represented ~49.0% of all sequences of the TM-1 assembly, including Gypsy-type transposons (41.7%) and Copia-type transposons (7.4%), and the LTR composition of the ZM24 assembly was similar to TM-1 (**Page 8, line 137 to 141**)".

- L141: What do these numbers mean? The relevance should be clearer.

Answer: The digits here refer to the numbers of the high-confidence full-length LTRs in each genome assembly. Now the sentence reads "Furthermore, the LTRs insertion times were estimated using the EMBOSS package, which respectively identified 33,908, 6,933, 41,085, and 38,206 high-confidence full-length LTRs in the *G. arboreum* (A_2), *G. raimondii* (D_5), TM-1, and ZM24 genomes" (**Page 9, line 149-152**).

- L301: What is meant by 'closely linked' in this sentence?

Answer: We were here trying to indicate that these are closely genetically linked. We have now revised this to read " SV1 is closely genetically linked with SV3" (**Page 19, line 345**). Thanks!

- L348-355: This sentence is too long and incomprehensible.

Answer: Thanks! We have revised it. Now the sentence reads "Viewed together, our recombination frequency and haplotype results collectively strongly suggest the possibility that the suppression of the recombination (and thus genetic exchange) resulting from the inversion regions on A08 have,

over time, separated the upland cotton population into two distinct groups" (**Page 22, line 396 to 399**).

- L867: 'd,' should be removed.

Answer: Thanks! Done! See **page 29 line 536**.

We thank Reviewer #2 for their constructive comments.

Reviewer #3 (Remarks to the Author):

In the manuscript by Yang et al, the authors generate long-read assemblies of two upland cotton cultivars and use these data for comparative analyses with the parental diploids to draw conclusions regarding genome evolution after whole genome duplication. The authors conclude that major genomic rearrangement has led to reproductive isolation and divergence of the two cultivars and, through phylogenetic analysis of these regions in the diversity panel, show that this rearrangement corresponds to the evolutionary relationships of included taxa. I believe the molecular work and bioinformatic analyses (with minor exceptions) to be robust, although not necessarily presented as well as it could be. Specific comments as they appear in the manuscript:

142-146: The transposable element analysis appears to be thrown in for no reason. Perhaps to demonstrate the quality of the assembly by comparison to other analyses of TE composition in cotton? Additionally, I question the methods used to estimate insertional timing. The authors state that the D subgenome amplification occurred within the last 1my and the A subgenome within the last 2my; however, these subgenomes co-exist within the same nucleus, so one should expect insertions to occur randomly throughout the entire genome.

Answer: Thanks very much! We have moved the transposable element analysis to the genome sequencing, assembly, and annotation section. We have supplemented the LTR expansion of the two ancestral diploid cotton, which revealed that the LTR insertion pattern of A_t subgenomes are similar with A genome. However, because the assembly of *G. raimondii* was based on short-reads, a large number of LTRs are collapsed, resulting in an LTR insertion pattern that is problematic to interpret. Based on insertion pattern of the A_t subgenome and the A genome, we now present a case arguing that most of the LTR expansion likely occur prior to polyploidization (**Page 9, line 145 to 163)**.

148: "random changes" - do we really know that these rearrangements are random?

Answer: Thanks for bringing this to our attention. We have removed the word "random", so the sentence now reads "The combination of different genomes in allopolyploids is known to result in changes in genome organization" (**Page 10, line 169**).

pgs 10-11: I really like figure 1. I was disappointed that there was no discussion of the evolutionary implications of the rearrangements depicted here. Since ZM24 and TM-1 contain the same rearrangements (mostly), and this is a very young polyploid, it is interesting to note that most of these probably occurred very quickly after the genome doubling event, pre-divergence of the two cultivars. Theories regarding structural rearrangement during polyploid stabilization could have been discussed in depth in the discussion section. Like the TE analysis, this was an interesting topic that was mostly just skipped over.

Answer: Thanks very much for this encouragement. We have supplemented the content about the evolutionary implications of the rearrangements in the revised discussion section (**Page 11, line 193 to 197**). Further, we introduce the current thinking about structural rearrangements during polyploid stabilization and explore the known roles of TEs in rearrangements during the polyploidizaiton processes (**Page 22, line 416-427**).

222-226: Was there any verification performed for a subset of these variants? One bp indels could very well be sequencing and assembly errors. The authors did not use an extremely robust trim and filter protocol for the short reads, and PacBio is relatively error prone.

Answer: Thanks very much. Yes, the low-quality reads were filtered using the following conditions: 1) reads with ≥10% unidentified nucleotides (N), 2) reads with >10 nucleotides aligned to the adapter, allowing ≤10% mismatches, 3) reads with >50% bases having Phred quality <5. And the clean reads were used to correct low-quality nucleotides and insertions/deletions (InDels) from the PacBio sequencing. We added the methods of correction PacBio contigs using Illumina short reads (See method section **page 33, line 620 to 623**).

232: The title for this section seems like a stretch. I would not call these finding for gene order "extensive" by any means. In addition, the results for mutations and other types of variants are included in this section. It was hard to follow exactly what "gene pairs" and "genes" the authors were discussing. This section was a bit confusing.

Answer: Thanks very much. We now understand that this was inappropriate and have changed the tittle of this section to "Gene-order and structural variation between TM-1 and ZM24" (**Page 15, line 268**). We have also uniformly referred to the genes and gene pairs as "orthologous gene pairs" (**Page 15-17, line 271-273, 278-284, 288, 291-292, 297-298, 301-302, 305, 307-310 and 312**).

269-271. "...these variations likely represent a substantial portion of the phenotypic differences

between these two accessions". This is a stretch and completely ignores the major effects of expression variation and epigenetic modification on phenotypic differentiation. The authors do not present any evidence to support this statement.

Answer: Thanks very much. We have revised the statement, which now reads "Approximately 10% of the annotated orthologous gene pairs had amino acid variation between the TM-1 and ZM24, and these variations can likely explain some of the phenotypic differences between these two accessions (**Page 17, line 312-314**).

Discussion section: This entire section was disappointing. In addition to the writing, which was difficult to decipher, the authors mostly discuss the quality of the assemblies and how they will be a valuable resource to the community. It contains relatively few biological insights.

Answer: Thanks very much for encouraging us to increase the scope of the discussion. We have substantially rewritten the section, and now include increased emphasis on topics including polyploidy and the impact of inversions on lint percentage (**Page 23, line 416-427 and 476-484**).

Figures: The figures are great. My only comment is for figure 2 (the circos plot). I can barely see the structural variants. The inversion is very clear, but I can barely see the intra translocation and cannot tell if there is an inter translocation (although I am assuming there is since it is in the legend).

Answer: Thanks very much for this complement! We have revised Figure 2 as directed.

There are numerous typos and grammatical issues throughout.

Answer: Thanks very much. To address this, we have invited a native English-speaking American with a Ph.D degree in Plant Biology to read and revise the whole manuscript for us. All his corrections about grammar and usage are tracked in the text.

Again, we are very grateful for the constructive comments offered by Reviewer #3.

Reviewers' comments:

Reviewer #1 (Remarks to the Author):

Nature Genetics published the reference-grade genome assembly and annotation of TM-1. Authors didn't cite this new publication and no comparisons were performed in this revision. I hope you make a comparison with this the reference-grade genome assembly in your revised manuscript.
Since you sequenced ZM24 and developed (TM-1 ZM24)RILs, we hope to know why there is no transgenic barrier in ZM24, or that probably some functional validation of the candidate genes is behind this transforming traits, otherwise no useful to sequence this genome. I think it is why you sequenced this cultivar. I suggest you conduct some RNA seq data during callus production of TM-1 and ZM24 to explore why the cotton transgenic operation is no genotypic dependency in ZM24.

Reviewer #2 (Remarks to the Author):

The manuscript by Yang et al. is significantly improved. Most of my comments have been worked out. There are still two items that have been poorly dealt with. The discussion of the relation between Lint percentage and the A08 inversion region is mentioned but hardly worked out. Secondly, the extremely descriptive paragraphs have been improved, but they remain very descriptive. What is the biological meaning/significance that "more than 13% of the syntenic orthologous gene pairs had large structural variations, and 12% of the syntenic orthologous gene pairs had lost at least one exon (see L301-303)"? The Results paragraphs are full with these percentages and numbers, but the paper does explain if these figures are normal or significant or just interesting for specialized readers to mention. The significance of the percentages and numbers are not discussed in Results or Discussion. This would make the paper more accessible for a larger public. In December 2018 a paper appeared in Nature Genetics by the group of Xianlong Zhang (Wang et al. 2018, Nat. Genet. 51, 224-229) on the genome sequence of G. hirsutum and G. barbadense. This work should also be included in the discussion.

Reviewer #3 (Remarks to the Author):

This manuscript is a much improved version over the original submission. The authors have addressed my concerns, specifically regarding conveyance of the biological relevance of their work. There remains numerous typos and/or grammatical issues that will have to be addressed before the manuscript is publication-worthy. I will address these below.

Besides the specifics below, the authors appear to have pulled back to an overly cautious position in which they do not commit to their findings. This is apparent in their overuse of words such as "likely" and "possibly", especially in areas where there really is no question about the interpretation. It makes the manuscript read as if they really did not really discover anything substantial, and I found it quite distracting.

Specific comments:

37: "Old world" - capitalize World

58: "comprised" - grammatically should read "are composed of"

64: "potentially thusly" - an example of what I consider timid writing.

69: I do not know what is meant by "is now planted to transgenic varieties"

74: delete the words "reference genomes"

96 and 97: this sentence uses respectively twice - redundant

139 and 140: gypsy and copia are retrotransposons. Transposon is typically reserved for Type II DNA elements.

160: I do not know what is meant by "LTRs insertion time pattern results

162: delete "likely"

173: delete "likely"

206: delete "likely". You already used "indicate" which implies some level of uncertainty. Therefore, "likely" is unnecessary.

223: use of the work "significantly" without a statistic to support

224: change "those" to "the"

231: I do not understand the inclusion of the word "number" in the parenthases

234: change "structure" to "structural".

234: change "numbers" to "number"

235: "very similar"? What does this even mean? What does similar mean? The word "very" is quite subjective.

241: clarify what you mean by "trace these genes"

248: delete the comma

260: delete "we detected in the genome". Your readers already know this.

261: insert "in" between "base-pair" and "length"

276: delete "were on chromosomes" because where else would they be?

279: insert "the" before At

280: change "subgenome" to "subgenomes"

287: Not sure about "in CDS or CDS and intron region". This seems to be a typo including a reference to CDS twice.

292: change "32,96" to "3,296"

313 and 314: I maintain that you do not have enough information to make this claim, as mentioned in my original review. Amino acid variation does not always equal phenotypic effects, and indeed, phenotypes can be controlled at the epigenetic or expression levels much more frequently than by changes in protein coding sequence. This claim is overreaching without data to support it.

315: insert "in" after "the"

329: Delete "very". Again, this is subjective. What is very to you might not be very to me. Not measurable.

330-333: Delete most of this. It is redundant, especially this far in to the manuscript. I suggest the following: Three large-scale inversions (larger than 4Mb) were found on A08 in the tetraploid assemblies and were further confirmed by..."

334: delete "clearly". unnecessary

339 and 340: change "an structural variation region" to "a structurally variant region"

345: delete "closely". Something is linked or not.

370: change "owning" to "owing"

371: delete "former"

374: Is this really "striking"? I think you found exactly what someone should expect to find. Yeah, recombination is suppressed within inversion regions. This is not a surprise.

391: Delete "Of considerable not". unnecessary

394: Delete "Of note". unnecessary

397: "strongly suggest the possibility that". Again, hedging. Just delete "the possibility". The word suggest already implies some level of uncertainty.

407: delete "our".

410: delete "potentially". see comments for 397

416: Add a period after angiosperms and then start a new sentence with More. As it stands, it is a run-on sentence. Add the word "of" after 70%

418. Need a reference for the "70% of flowering plants" statement

431: delete "the" before "large-scale"

437: indicating and probably: again feels like overstating some uncertainty. Only one of them is needed.

456: I don't know what is meant by "such as inversions and these chromosome differences"

460: I don't know what is meant by "first time in nature".

471 and 472: indicate and possible: see comments for 437

472: delete "relatively".

Reviewers' comments:

Reviewer #1 (Remarks to the Author):

Nature Genetics published the reference-grade genome assembly and annotation of TM-1. Authors didn't cite this new publication and no comparisons were performed in this revision. I hope you make a comparison with this the reference-grade genome assembly in your revised manuscript. Since you sequenced ZM24 and developed (TM-1 ZM24) RILs, we hope to know why there is no transgenic barrier in ZM24, or that probably some functional validation of the candidate genes is behind this transforming traits, otherwise no useful to sequence this genome. I think it is why you sequenced this cultivar. I suggest you conduct some RNA seq data during callus production of TM-1 and ZM24 to explore why the cotton transgenic operation is no genotypic dependency in ZM24.

Response: Thanks for this suggestion. We have conducted a comparison of our genome sequences with the recently published TM-1 and 3-79 genomes and our manuscript now includes this and discusses differences relating to sequencing platform, assembly strategy, contig contiguity, anchored and oriented ratio; we also detail a whole genome comparison between our genomes and their genomes. Some pertinent differences we found are that, compared with Wang et al.'s TM-1 genome, our contig contiguity was improved by 2.51 folds (4,760 Kb vs 1,891 Kb), and we also found that, except for the D08 chromosome, other chromosomes kept good collinearities with each other in both studies. We examined this in greater detail and a Hi-C heatmap showed that the inversions on D08 were due to the misassemblies in Wang et al's sequence. It is also notable that we have 3,500 more gene models in our gene prediction results, and our gene models cover more than 95% (67,160 of 70,199) of the genes predicted based on Wang et al's genome (**Supplementary Fig. 5, Supplementary Table 3 and 7**; **See page 7, line 115-124; Page 8, line 136-137; Page 29-31，line 546-572**).

Further, as suggested, we have now included an RNAseq analysis of TM-1 and ZM24 in callus cultures which shows revealed many differentially expressed genes (DEGs) in the two cultivars; we have added a substantial new amount of text to the results section wherein we detail multiple genes with expression profiles characteristic of putative transgenic-barrier-related genes. Very briefly, our data strongly implicate altered hormone metabolism in calli as a major potential source of transgenic-barrier-related differences between the TM-1 and ZM24 cultivars, including ZM24-specific accumulation of regulatory and structural genes for auxin metabolism and polar transport as well as regulatory and catabolism genes of Gibberellin metabolism. Our revised manuscript includes discussion of these findings and proposes falsifiable hypotheses which could verify a transgenic-barrier-related contribution of these genes. Highlighting the utility of comparative genomics amongst multiple cultivars from a given species, it was highly notable that we detected genetic polymorphisms between the TM-1 and ZM cultivars for many of these transformation-barrier related genes from hormone metabolism: for example SNPs in the coding region of *GhAGL15* (Gh_A12G116000 and Gh_A08G163400) and SNPs or InDels in the promoters of *GhAGL15* (Gh_A12G116000 and Gh_D08G112500) and *GhLEC1* (Gh_A13G151400). We thank the reviewer for prompting us to undertake these revealing studies, as our results open the door for functional further studies that should clarify the unique characteristics which have made ZM24 an important biotech cultivar (**Supplementary Fig. 21**; **See page 18-21, line 337-388**).

Again, we would like to thank Reviewer #1 for their constructive comments and guidance.

Reviewer #2 (Remarks to the Author):

The manuscript by Yang et al. is significantly improved. Most of my comments have been worked out. There are still two items that have been poorly dealt with. The discussion of the relation between Lint percentage and the A08 inversion region is mentioned but hardly worked out. Secondly, the extremely descriptive paragraphs have been improved, but they remain very descriptive. What is the biological meaning/significance that "more than 13% of the syntenic orthologous gene pairs had large structural variations, and 12% of the syntenic orthologous gene pairs had lost at least one exon (see L301-303)"? The . The significance of the percentages and numbers are not discussed in Results or Discussion. This would make the paper more accessible for a larger public. In December 2018 a paper appeared in Nature Genetics by the group of Xianlong Zhang (Wang et al. 2018, Nat. Genet. 51, 224-229) on the genome sequence of G. hirsutum and G. barbadense. This work should also be included in the discussion.

Response: Thanks very much. Based on your guidance about the superficiality of our consideration of any relationship between Lint percentage and the A08 inversion region, and deciding to error on the side of caution, we have decided to delete this part of the Discussion. Regarding your second point about making our study more accessible to a larger public by better presenting information about biological significance in the results, we have now included new text that better contextualizes our findings into the larger research area of domesticated plant genomes (via a comparison with reports for intra-cultivar structural variations from maize) and the effects of polyploidization on gene evolution (we discuss proposals from wheat) (**See page 17, line 309-313**). As directed, we have also conducted a comparison of our genome sequences with the recently published TM-1 and 3-79 genomes and our manuscript now includes this and discusses differences relating to sequencing platform, assembly strategy, contig contiguity, anchored and oriented ratio; we also detail a whole genome comparison between our genomes and their genomes. Some pertinent differences we found are that, compared with Wang et al.'s TM-1 genome, our contig contiguity was improved by 2.51 folds (4,760 Kb vs 1,891 Kb), and we also found that, except for the D08 chromosome, other chromosomes kept good collinearities with each other in both studies. We examined this in greater detail and a Hi-C heatmap showed that the inversions on D08 were due to the misassemblies in Wang et al's sequence. It is also notable that we have 3,500 more gene models in our gene prediction results, and our gene models cover more than 95% (67,160 of 70,199) of the genes predicted based on Wang et al's genome (**Supplementary Fig. 5, Supplementary Table 3 and 7**; **See page 7, line 115-124; Page 8, line 136-137; Page 29-31 , line 546-572**).

Again, we would like to thank Reviewer #2 for their constructive comments and guidance.

Reviewer #3 (Remarks to the Author):

This manuscript is a much improved version over the original submission. The authors have addressed my concerns, specifically regarding conveyance of the biological relevance of their work. There remains numerous typos and/or grammatical issues that will have to be addressed before the manuscript is publication-worthy. I will address these

below.

Besides the specifics below, the authors appear to have pulled back to an overly cautious position in which they do not commit to their findings. This is apparent in their overuse of words such as "likely" and "possibly", especially in areas where there really is no question about the interpretation. It makes the manuscript read as if they really did not really discover anything substantial, and I found it quite distracting.

Response: Many thanks for your care in directing our prose usage in our manuscript: going through these changes under your direction has been very informative for us. In light of your guidance, we now see our error in going too far towards the 'gun shy' stance with our conclusion statements in our revised text. Your comprehensiveness in showing us this tendency has edified our understanding about how to use prose more effectively in supporting our scientific arguments based on their individual merit & rigor. Your notes also helped us to see how our attempts to add emphasis with phrases like 'on the chromosomes', as well as our abuse of modifiers like 'striking' can seem rather silly. In the current draft of the manuscript, and moving forward, we are and will be more cautious about employing timidity/hedging qualification words and phrases, and we will be more conscious of trying to let the obvious biological context— rather than superfluous words—emphasize our conclusions. We are now much better placed to fight these tendencies in our writing; so again, many thanks.

Specific comments:

37: "Old world" - capitalize World

Response: Thanks very much. Done! See page 3, line 37.

58: "comprised" - grammatically should read "are composed of"

Response: Thanks very much. Done! See page 4, line 59.

64: "potentially thusly" - an example of what I consider timid writing.

Response: Thanks very much. We deleted the word “potentially”. Now the sentence reads “Thus, seeking to identify genomic features that could facilitate increasingly high-resolution technologies for breeding efforts in cotton, we here used PacBio SMART long read technology to obtain sequence data from both the genetic standard TM-1 and the biotechnologically important ZM2420-22 cotton cultivars” (**See page 4, line 66-70**).

69: I do not know what is meant by "is now planted to transgenic varieties"

Response: Thanks very much. We revised the sentence by changing 'to' to 'with' so it now reads “A majority of the cotton acreage in many countries is now planted with transgenic varieties…” (**See page 4-5, line 70-74**).

74: delete the words "reference genomes"

Response: Thanks very much. Done! **See page 5, line 75**.

96 and 97: this sentence uses respectively twice – redundant

Response: Thanks very much. We deleted the second one. **See page 6, line 99**.

139 and 140: gypsy and copia are retrotransposons. Transposon is typically reserved for Type II DNA elements.

Response: Thanks very much bringing this issue to our attention. We have now replaced "transponson" with retrotransposons, so the sentence now reads as "Long terminal repeats (LTR) represented ~49.0% of all sequences of the TM-1 assembly, including *Gypsy*-type retrotransposons (41.7%) and *Copia*-type retrotransposons (7.4%), and the LTR composition of the ZM24 assembly was similar to TM-1" (**See page 8-9, line 146-149**) .

160: I do not know what is meant by "LTRs insertion time pattern results

Response: Thanks very much. We revised the statement as "Based on TE divergence and LTR insertion times for the A genome and A_t subgenome (Supplementary Fig. 11), we conclude that most of the TEs expanded in the progenitor genomes and were then retained after allopolyploid formation (**See page 9, line 164-167**).

162: delete "likely"

Response: Thanks very much. Done! **See page 9, line 166**.

173: delete "likely"

Response: Thanks very much. Done! **See page 10, line 179**.

206: delete "likely". You already used "indicate" which implies some level of uncertainty. Therefore, "likely" is unnecessary.

Response: Thanks very much. Done! **See page 12, line 212**.

223: use of the work "significantly" without a statistic to support

Response: Thanks very much. We deleted the word "significantly". **See page 13, line 230.**

224: change "those" to "the"

Response: Thanks very much. Done! **See page 13, line 230**.

231: I do not understand the inclusion of the word "number" in the parenthases

Response: Thanks very much. Our intent here had been that the number in the parenthases indicated the PAV number; we have now revised this so that the sentence now reads "for example ZM24 A08 (1,847 PAVs · ~7.9 Mb) had significantly more PAVs than the other chromosomes" (**See page 13, line 230**.).

234: change "structure" to "structural".

Response: Thanks very much. Done! **See page 13, line 241.**

234: change "numbers" to "number"

Response: Thanks very much. Done! **See page 13, line 241.**

235: "very similar"? What does this even mean? What does similar mean? The word "very" is quite subjective.

Response: Thanks very much. We removed the word "very". Now the sentence reads "The number of PAVs between TM-1Dt subgenome (4,639) and ZM24 Dt subgenome (4,875) were similar" (**See page 13, line 242-243**).

For context we were here seeking to highlight that comparisons of the number of PAVs was biologically informative because the number was similar for the D_t subgenome but was quite different for the A_t subgenome; a finding indicating that more extensive genetic variation A_t subgenome has occurred relative to the D_t subgenome.

241: clarify what you mean by "trace these genes"

Response: Thanks very much. We have revised the statement. Now the sentence reads “We aligned the genes located on pseudochromosomes with the G. arboreum and G. raimondii genomes and found that approximately 58% and 69% of the PAV genes from the TM-1 A_t subgenome and ZM24 A_t subgenome have likely orthologs in *G. arboreum*” (**See page 14, line 249**).

248: delete the comma

Response: Thanks very much. Done! **See page 14, line 256.**

260: delete "we detected in the genome". Your readers already know this.

Response: Thanks very much. Done! **See page 15, line 267.**

261: insert "in" between "base-pair" and "length"

Response: Thanks very much. Done! **See page 15, line 268.**

276: delete "were on chromosomes" because where else would they be?

Response: Thanks very much. We revised the statement. Now the sentence reads “A total of 34,634 (~96.1%) orthologous pairs within the A_t subgenomes and 34,518 (~96.3%) orthologous gene pairs within the D_t subgenomes were anchored in the 26 pseuduchromosomes, and these were used for our gene-order and structure comparisons ” (**See page 15, line 280-283**).

279: insert "the" before At

Response: Thanks very much. Done! **See page 16, line 286.**

280: change "subgenome" to "subgenomes"

Response: Thanks very much. Done! **See page 16, line 288.**

287: Not sure about "in CDS or CDS and intron region". This seems to be a typo including a reference to CDS twice.

Response: Thanks very much. We revised the statements as “Among the syntenic genes, 23,963 orthologous gene pairs from A_t subgenomes displayed no amino acid changes between TM-1 and ZM24, and approximately 68% and 56% showed no variation in coding sequence (CDS) or gene bodies (CDS and intron region), respectively” (**See page 16, line 291-294**).

292: change "32,96" to "3,296"

Response: Thanks very much. Done! **See page 16, line 299**.

313 and 314: I maintain that you do not have enough information to make this claim, as mentioned in my original

review. Amino acid variation does not always equal phenotypic effects, and indeed, phenotypes can be controlled at the epigenetic or expression levels much more frequently than by changes in protein coding sequence. This claim is overreaching without data to support it.

Response: Thanks very much. We have removed the claim. Now the sentence reads “Approximately 10% of the annotated orthologous gene pairs had amino acid variation between the TM-1 and ZM24; any biological significance of these variations need will require further study (**See page 16, line 323**).

315: insert "in" after "the"

Response: Thanks very much. Done! **See page 17, line 326**.

329: Delete "very". Again, this is subjective. What is very to you might not be very to me. Not measurable.

Response: Thanks very much. Done! **See page 21, line 396**.

330-333: Delete most of this. It is redundant, especially this far in to the manuscript. I suggest the following: Three large-scale inversions (larger than 4Mb) were found on A08 in the tetraploid assemblies and were further confirmed by..."

Response: Thanks very much for this suggestion. We have revised this so it now reads “Three large-scale inversions (larger than 4 Mb) were found on A08 in the tetraploid assemblies; these were confirmed by mapping the Hi-C data of one accession to the genome of the other and vice versa (**See page 21, line 396-399**)”.

334: delete "clearly". Unnecessary

Response: Thanks very much. Done! **See page 21, line 399**.

339 and 340: change "an structural variation region" to "a structurally variant region"

Response: Thanks very much. Done! **See page 22, line 405-406**.

345: delete "closely". Something is linked or not.

Response: Thanks very much. Done! **See page 22, line 411.**

370: change "owning" to "owing"

Response: Thanks very much. Done! **See page 23, line 436.**

371: delete "former"

Response: Thanks very much. Done! **See page 23, line 437.**

374: Is this really "striking"? I think you found exactly what someone should expect to find. Yeah, recombination is suppressed within inversion regions. This is not a surprise.

Response: Thanks very much. We removed the word “striking”. Now the sentence reads “When we overlapped the SV1 and SV3 regions with meiotic recombination frequency data between TM-1 and ZM24, we observed strong associations between suppressed recombination rates and the loci contained within the inversion regions” (**See page 24, line 440-443**).

391: Delete "Of considerable not". Unnecessary

Response: Thanks very much. Done! **See page 24, line 457.**

394: Delete "Of note". Unnecessary

Response: Thanks very much. Done! **See page 25, line 460.**

397: "strongly suggest the possibility that". Again, hedging. Just delete "the possibility". The word suggest already implies some level of uncertainty.

Response: Thanks very much. Done! **See page 25, line 463.**

407: delete "our".

Response: Thanks very much. Done! **See page 25, line 470.**

410: delete "potentially". see comments for 397

Response: Thanks very much. Done! **See page 25, line 473.**

416: Add a period after angiosperms and then start a new sentence with More. As it stands, it is a run-on sentence. Add the word "of" after 70%

Response: Thanks very much. Done! Now the sentence reads "Polyploidy is a widespread phenomenon in angiosperms. More than 70% of flowering plants have undergone polyploidization at some point in their evolutionary history36". (**See page 26, line 479-481**).

418. Need a reference for the "70% of flowering plants" statement

Response: Thanks very much. Done! **See page 26, line 479-481.**

431: delete "the" before "large-scale"

Response: Thanks very much. Done! **See page 26, line 495.**

437: indicating and probably: again feels like overstating some uncertainty. Only one of them is needed.

Response: Thanks very much. Done! **See page 27, line 501.**

456: I don't know what is meant by "such as inversions and these chromosome differences"

Response: Thanks very much. We revised the statements. Now the sentence reads "Chromosomal rearrangements such as inversions and translocations have long been thought to play a critical role in adaptation and speciation" (See **page 28, line 516-518).**

460: I don't know what is meant by "first time in nature".

Response: Thanks very much. We have removed this "first time in nature". Now the sentence reads "David *et al*. discovered that a chromosomal inversion polymorphism is geographically widespread, and their results demonstrated that the contribution of (i) an inversion to adaptation, (ii) an annual/perennial life-history shift, and (iii) multiple reproductive isolating barriers" (**See page 28, line 519-522)**.

471 and 472: indicate and possible: see comments for 437

Response: Thanks very much. We removed the word “possible”. **See page 28, line 532**

472: delete "relatively".

Response: Thanks very much. Done! **See page 29, line 533.**

Let us again gratefully acknowledge our appreciation for your highly informative guidance in this revision.

Reviewers' comments:

Reviewer #1 (Remarks to the Author):

Most of my comments which I raised have been revised. TM-1 genome sequence has been published in Nat Genet. So, I am interested in large-scale inversions on chromosome A08 are widely distributed and have over time mediated the reduction of meiotic recombination that has ultimately driven distinctly genetically isolated haplotypes of G. hirsutum. From this R2 revision, I found some new problems or some new suggestions.

Moreover, a total of 127 inversions and 2,127 translocations were identified between TM-1 and ZM24. I do not believe that there are so high structure variations in the intraspecific cultivars. They only detected interspecific 74 genomic rearrangements including inversion and translocation between the diploid A2 genome and TM-1 At subgenome (Suppl. Table 10), and 44 between the diploid D5 genome and TM-1 Dt subgenome (Suppl. Table 11). How to explain? It is really higher extensive intraspecific gene order and gene structural variations existed in upland cotton than interspecific species. I still suspect their assembly.

Since you conducted genomic comparison among the TM-1 At subgenome, the TM-1 Dt subgenome, the ZM24-At subgenome, the ZM24-Dt subgenome, D5 (G. raimondii, JGI) and the A2 genome (G. arboreum, ICR of CAAS) (a) and between TM-1 and ZM24 (b), what is the relationship in large-scale inversions on chromosome A08 among these species in Supplementary Figure 12. Or which is the wild type for large-scale inversions on chromosome A08, TM-1 and/or ZM24?

You think D08 may be mis-assembled in HUA/TM-1 genome. I do not think they have enough data to support their assembly of TM-1 is correct or not. I suggest you compare to D08 chromosome in diploid D genome species (G. raimondii), very well assembled. In Supplementary Figure 5, I surprisingly find the same in this region between TM-1 and ZM24 and G. barbadense, no inverted region or no marked. I cannot understand it. Furthermore, you conducted genomic comparison between the TM-1 Dt subgenome in this study with the previously published sequence (NAU), what about this region?

It is interested they make a genomic comparison between the TM-1 Dt subgenome in this study with the previously published sequence by NAU (Nat Biotechnol 33:531-537) in Supplementary Figure 4, rather than with themselves (Nat Biotechnol 33:521-530) to present what is their improvement in TM-1 assembly.

Reviewer #2 (Remarks to the Author):

The ms. by Yang et al. is further improved. They have added more text explaining biological significance. They also added a discussion on the recently published paper by Wang et al. (2018) on the G. hirsutum and G. barbadense genome sequences. The message in this discussion (L546-573), however, is not clear, because the text is presented as additional results, comparing the data sets of the two studies. This discussion should be more to the point.

The authors state that “ecological traits linked to inversions have been poorly studied” (see L518-519). They refer to papers on this topic, including the Arabidopsis inversion on chromosome 4, but they miss a publication on this inversion in Arabidopsis, in which (1) strong association is reported between the inversion and fecundity under drought conditions, and (2) linkage disequilibrium is found between the inversion and the early flowering Col-FRIGIDA allele (see Fransz et al., 2016, Plant J. 88, 159). In L519 they write “..David et al. discovered that …”. This reference is not mentioned in the reference list. The ref. nr. (48) at the end of the sentence is not correct. The same accounts for Diego et al. in L523.

Editors' comments

Editorially, we think it is critical to compare D08 chromosome with the published diploid D genome (as Reviewer #1 suggested) and the latest assemblies of the TM-1 and G. barbadense cv. Hai7124 genomes (https://www.nature.com/articles/s41588-019-0371-5) to justify your claim that the D08 chromosome of previously published TM-1 genome (https://www.nature.com/articles/s41588-018-0282-x) was mis-assembled. In addition, Reviewer #1 concerns the assembly quality as comparatively large number of intraspecific vs interspecific translocations were identified; we would like to hear your explanations on this specific issue.

Response: Thanks very much! We have supplemented our TM-1 D08 with the published diploid D genome and the latest assemblies of the TM-1 and *G. barbadense* cv. Hai7124 genomes. We found no inversions between our TM-1 D08 sequence with the diploid D genome or the new TM-1 and G. barbadense cv. Hai7124 genome assemblies. On the contrary, we found inversions between TM-1_HAU D08 with the other assemblies for TM-1 and for the other species (we elaborate on this topic in our response to Reviewer #1's penultimate comment, below; also see RESPONSE DOCUMENT TABLE #1).

Regarding the question about intraspecific vs. interspecific translocations, there was a fundamental misunderstanding about these comparisons based on our lack of clarity in the text. Specifically, we did not compare the raw output from the relevant comparisons, but rather processed the interspecific data into syntenic blocks before we considered its number of rearrangements. For clarity, our analysis proceeded in the following steps.

1) Using nucmer of Mummer with two sets of parameters settings: '-i 90 -1 -r -q' and '-i 90 -g -r -q', and the assembly sequences anchored and oriented on chromosomes as inputs, we found that there were 13,819 rearrangements (translocations+inversions) between TM-1 A_t and A_2; 7,492 rearrangements (translocations+inversions) between TM-1 D_t and D_5; and 2,254 (translocations+inversions) between TM-1 and ZM24. So, these results clearly show that the number of interspecific rearrangements is much larger than the intraspecific rearrangements.

2) In our previously submitted drafts, we conducted another data processing step that led to our reported numbers (64 translocations and 54 inversions) for the interspecific comparisons. Specifically, this data processing was undertaken based on that fact that the total length of rearrangements between TM-1 A_t/D_t and A_2/D_5 occupied ~620 Mb, which was ~ 12 fold more than the total length of rearrangements between the intraspecific comparison of TM-1 and ZM24 (occupying 51.2 Mb). To help manage this substantial difference in the magnitude of the rearranged sequences, we manually combined the small aligned blocks into larger and more continuous rearrangement regions for the interspecific comparisons—between TM-1 A_t and A_2 (*G. arboreum*) as well as between TM-1 D_t with D_5 (*G. raimondii*).

Having now clarified the impact of this additional data processing step for the interspecific comparison, it is now clear that our data show a larger number of interspecific rearrangements than intraspecific rearrangements: 2,254 detected intraspecific rearrangements vs. 7,492 (& 13,819) detected interspecific rearrangements (**See Page 10 and 11, line 181-195; Page 42, line 790-800**).

POINT-BY-POINT RESPONSE DOCUMENT

Reviewers' comments:

Reviewer #1 (Remarks to the Author):

Most of my comments which I raised have been revised. TM-1 genome sequence has been published in Nat Genet. So, I am interested in large-scale inversions on chromosome A08 are widely distributed and have over time mediated the reduction of meiotic recombination that has ultimately driven distinctly genetically isolated haplotypes of G. hirsutum. From this R2 revision, I found some new problems or some new suggestions.

Moreover, a total of 127 inversions and 2,127 translocations were identified between TM-1 and ZM24. I do not believe that there are so high structure variations in the intraspecific cultivars. They only detected interspecific 74 genomic rearrangements including inversion and translocation between the diploid A2 genome and TM-1 At subgenome (Suppl. Table 10), and 44 between the diploid D5 genome and TM-1 Dt subgenome (Suppl. Table 11). How to explain? It is really higher extensive intraspecific gene order and gene structural variations existed in upland cotton than interspecific species. I still suspect their assembly.

Response: Thanks very much, for highlighting our lack of clarity about this, which we now understand and have updated accordingly. We did not clearly note this in the previously submitted drafts of the manuscript, but kindly note that we did not compare the raw output from the relevant comparisons, but rather processed the interspecific data into syntenic blocks before we considered its number of rearrangements. For clarity, our analysis proceeded in the following steps.

1) Using nucmer of Mummer with two sets of parameters settings: '-i 90 -1 -r -q' and '-i 90 -g -r -q', and the assembly sequences anchored and oriented on chromosomes as inputs, we found that there were 13,819 rearrangements (translocations+inversions) between TM-1 A_t and A_2; 7,492 rearrangements (translocations+inversions) between TM-1 D_t and D_5; and 2,254 (translocations+inversions) between TM-1 and ZM24. So, these results clearly show that the number of interspecific rearrangements is much larger than the intraspecific rearrangements.

2) In our previously submitted drafts, we conducted another data processing step that led to our reported numbers (64 translocations and 54 inversions) for the interspecific comparisons. Specifically, this data processing was undertaken based on that fact that the total length of rearrangements between TM-1 A_t/D_t and A_2/D_5 occupied ~620 Mb, which was ~ 12 fold more than the total length of rearrangements between the intraspecific comparison of TM-1 and ZM24 (occupying 51.2 Mb). To help manage this substantial difference in the magnitude of the rearranged sequences, we manually combined the small aligned blocks into larger and more continuous rearrangement regions for the interspecific comparisons between TM-1 A_t and A_2 (*G. arboreum*) as well as between TM-1 D_t with D_5 (*G. raimondii*).

Having now clarified the impact of this additional data processing step for the interspecific comparison, it is now clear that our data show a larger number of interspecific rearrangements than intraspecific rearrangements: 2,254 detected intraspecific rearrangements vs. 7,492 (& 13,819) detected interspecific rearrangements (**See Page 10 and 11, line 181-195; Page 42, line 790-800**).

Since you conducted genomic comparison among the TM-1 At subgenome, the TM-1 Dt subgenome, the ZM24-At subgenome, the ZM24-Dt subgenome, D5 (G. raimondii, JGI) and the A2 genome (G. arboreum, ICR of CAAS) (a) and between TM-1 and ZM24 (b), what is the relationship in large-scale inversions on chromosome A08 among these species in Supplementary Figure 12. Or which is the wild type for large-scale inversions on chromosome A08, TM-1 and/or ZM24?

Response: Thanks for prompting us to consider these ideas and comparisons. Our analysis during the present revision process focused on three rearrangement regions: SV1 (our TM-1 A08 29760606-47296785 bp), SV2 (our TM-1 A08 481145613-52751556 bp), and SV3 (our TM-1 A08 61196693-75890534 bp). For example, using the SV1 region, we found intra-chromosomal translocation and inversion between our TM-1 sequence and the *G. arboreum* genome, and found different inversions between ZM24 and the *G. arboreum* genome (**see Supplementary Figure 13 and Figure 1**). For comparisons of the SV1 region with the D genome progenitor species: different types of rearrangements were observed between TM-1 and *G. raimondii* and between ZM24 and *G. raimondii* (**see Supplementary Figure 13 and Figure 1**). Thus, on account of the various types and positions of the inversions and translocations for our TM-1 SV1-SV3 regions, it is difficult to conclude more than summary statements like "the polymorphisms observed among TM-1 A08 and ZM24 A08 and *G. arboretum*, or among TM-1 A08 and ZM24 A08 and *G. raimondii*, provide strong empirical support that the A08 chromosomes have been very active." Nevertheless, seeking to gain insights within upland cotton specifically, we then used two methods to group 419 upland cotton accessions into two populations (PCA and neighbor joining phylogeny, both based on polymorphism data for the entire genome). These analyses grouped the accessions into a ZM24-like group and a TM-1-like group.

With these groupings, and guided by previous research in Arabidopsis, we calculated the haplotype diversity across the entire A08 chromosome. Specifically, the Zapata *et al.* (2016) study showed that the group of accessions derived from the inversion-bearing progenitor had lower haplotype diversity within the inversion regions as compared to the group without the original inversion. Considering our finding that the haplotype diversity of chromosome A08 was dramatically decreased in the ZM24-like group vs. the TM-1-like group, and assuming that the trend reported for Arabidopsis is relevant to the situation in the upland cotton accessions we examined, our data support that a ZM24-like progenitor carried the initial inversion and that the TM-1 progenitor could therefore be conceptualized as the "wild type" genotype regarding the large-scale inversions in question (SV1, SV2, and SV3).

You think D08 may be mis-assembled in HUA/TM-1 genome. I do not think they have enough data to support their assembly of TM-1 is correct or not. I suggest you compare to D08 chromosome in diploid D genome species (G. raimondii), very well assembled. In Supplementary Figure 5, I surprisingly find the same in this region between TM-1 and ZM24 and G. barbadense, no inverted region or no marked. I cannot understand it. Furthermore, you conducted genomic comparison between the TM-1 Dt subgenome in this study with the previously published sequence (NAU), what about this region?

Response: Thanks very much for your suggestion, and thank you for bringing our attention to our omission of relevant marks in supplemental Figure 5. We have now compared our TM-1 assembly and

the TM-1_HAU assembly with the *G. raimondii* genome. As shown in updated supplementary Fig. 6, whereas our TM-1 D08 kept strong collinearity with D08 of *G. raimondii*, there were no inversions observed between our TM-1 D08 and D08 of *G. raimondii*. Moreover, we have now clearly marked the inverted regions between our TM-1 (and ZM24) with *G. barbadense* in supplementary Fig. 5 (**Also see supplementary Fig. 6b**). Comparison of our TM-1 D08 assembly with the previously published TM-1_NAU_D08 revealed strong collinearity. Further, genomic comparisons between our TM-1 assembly with the newly published TM-1 and Hai7124 assemblies also showed strong collinearities for D08. For a summary of these comparisons, please see RESPONSE DOCUMENT TABLE #1. We have substantially toned down the claims about previous assemblies in the revised text, which now reads as follows:

" A Hi-C heatmap indicated that our TM-1 was correctly assembled in the four inverted regions on D08 (**Supplementary Fig. 5**). We also compared our TM-1 D08 sequence with *G. raimondii*_D08, TM-1_ZJU_D08, Hai7124_ZJU_D08, and TM-1_NAU_D08, and found that our TM-1 D08 sequence showed strong collinearities with each of these assemblies (**Supplementary Fig. 6**). " (**See Page 7, line 114-119, Supplementary Figs. 5 and 6**).

TM-1-ICR_D08	Region1 (int1) 24658532-26490867 bp	Region2 (int2) 27180413-28389782 bp	Region3 (int3) 29363206-30200000 bp	Region4 (in4) 30408902-34019164 bp
*G. raimondii*_D08	NA	+	+	+
TM-1_ZJU_D08	+	+	+	+
Hai7124_ZJU_D08	+	+	+	+
TM-1_NAU_D08	NA	+	+	+

RESPONSE DOCUMENT TABLE #1. "+" indicates that the orientation of the region in question is the same as in our TM-1 assembly; "-" indicates that the orientation is the opposite compared to our TM-1 assembly. NA indicates that no homology blocks were found in the D08 compared to our TM-1 assembly.

It is interested they make a genomic comparison between the TM-1 Dt subgenome in this study with the previously published sequence by NAU (Nat Biotechnol 33:531-537) in Supplementary Figure 4, rather than with themselves (Nat Biotechnol 33:521-530) to present what is their improvement in TM-1 assembly.

Response: Thanks very much. Since the publication of the TM-1 genomes, the TM-1_NAU assembly has been widely used in cotton research and many studies are based on it; this guided our choice for comparing our new TM-1 data with the TM-1_NAU assembly.

Again, we would like to thank Reviewer #1 for constructive comments and guidance.

Reviewer #2 (Remarks to the Author):

The ms. by Yang et al. is further improved. They have added more text explaining biological significance. They also added a discussion on the recently published paper by Wang et al. (2018) on the G. hirsutum and G. barbadense genome sequences. The

message in this discussion (L546-573), however, is not clear, because the text is presented as additional results, comparing the data sets of the two studies. This discussion should be more to the point.

The authors state that “ecological traits linked to inversions have been poorly studied” (see L518-519). They refer to papers on this topic, including the Arabidopsis inversion on chromosome 4, but they miss a publication on this inversion in Arabidopsis, in which (1) strong association is reported between the inversion and fecundity under drought conditions, and (2) linkage disequilibrium is found between the inversion and the early flowering Col-FRIGIDA allele (see Fransz et al., 2016, Plant J. 88, 159). In L519 they write “..David et al. discovered that …”. This reference is not mentioned in the reference list. The ref. nr. (48) at the end of the sentence is not correct. The same accounts for Diego et al. in L523.

Response: Thanks very much for your suggestion. We have supplemented the details for our comparison between our two assemblies with those of Wang *et al.* in the revised discussion section (**See Page30-31, line 561-574)**. As you suggested, we add the text about the Fransz *et al.* study in the revised discussion section (**See Page28, line 524-528**). We also revised the text regarding references 48 and 49. Now the sentence reads:

“Lowry *et al*. [49] discovered that a chromosomal inversion polymorphism is geographically widespread, and their results demonstrated the contribution of an inversion to adaptation, an annual/perennial life-history shift, and multiple reproductive isolating barriers. Ayala *et al.* [50] studied the roles of chromosome inversions in the adaptation of the four major African malaria mosquitoes to local environments in Africa and found that most inversions are actively involved in processes of local adaptation” (**Page28, line 516-524**).

Again, we would like to thank Reviewer #2 for their constructive comments and guidance.

REVIEWERS' COMMENTS:

Reviewer #1: This reviewer states in Remark to Editor section that (s)he is satisfied with revision and suggests accepting the paper.

Reviewer #3 (Remarks to the Author):

I am satisfied with the authors new comparative analyses and explanations regarding rearrangements on A08. The bioinformatic analyses are robust and the authors verified some of the breakpoints via PCR. I enjoyed this manuscript.

REVIEWERS' COMMENTS:

Reviewer #1: This reviewer states in Remark to Editor section that (s)he is satisfied with revision and suggests accepting the paper.

Response: Thanks very much!

Reviewer #3 (Remarks to the Author):

I am satisfied with the authors new comparative analyses and explanations regarding rearrangements on A08. The bioinformatic analyses are robust and the authors verified some of the breakpoints via PCR. I enjoyed this manuscript.

Response: Thanks very much!
